# Multicohort analysis unveils axon guidance pathways linking small for gestational age to spirometric restriction

**DOI:** 10.1038/s41467-026-72490-w

**Published:** 2026-05-02

**Authors:** James F. Read, Debra A. Stern, Tara F. Carr, Amber L. Spangenberg, Meiven Yang, Rosa I. Luna-Ramirez, Stefano Guerra, Wayne J. Morgan, Alex T. Binder, Jeffrey J. VanWormer, Christine M. Seroogy, Rachel L. Miller, Edward M. Zoratti, Carole Ober, Daniel J. Jackson, Sean W. Limesand, Diane R. Gold, James E. Gern, CADRE consortium, Debra A. Stern, Tara F. Carr, Amber L. Spangenberg, Alex T. Binder, Jeffrey J. VanWormer, Christine M. Seroogy, Rachel L. Miller, Edward M. Zoratti, Carole Ober, Daniel J. Jackson, Diane R. Gold, James E. Gern, Fernando D. Martinez, Anthony Bosco, Fernando D. Martinez

**Affiliations:** 1 Asthma and Airway Disease Research Center, University of Arizona, Tucson, AZ USA; 2 Department of Immunobiology, The University of Arizona College of Medicine, Tucson, AZ USA; 3 School of Animal and Comparative Biomedical Sciences, University of Arizona, Tucson, AZ USA; 4 Office of Informatics and Information Technology, University of Wisconsin School of Medicine and Public Health, Madison, WI USA; 5 Center for Clinical Epidemiology & Population Health, Marshfield Clinic Research Institute, Marshfield, WI USA; 6 Department of Pediatrics, University of Wisconsin School of Medicine and Public Health, Madison, WI USA; 7 Division of Clinical Immunology, Icahn School of Medicine at Mount Sinai, New York, NY USA; 8 Division of Allergy and Clinical Immunology, Department of Internal Medicine, Henry Ford Health, Detroit, MI USA; 9 Department of Human Genetics, University of Chicago, Chicago, IL USA; 10 Department of Environmental Health, Harvard T. H. Chan School of Public Health, Boston, MA USA; 11 Channing Division of Network Medicine, Brigham and Women’s Hospital and Harvard Medical School, Boston, MA USA; 12 Department of Pediatrics, The University of Arizona College of Medicine, Tucson, AZ USA

**Keywords:** Proteomics, Pathogenesis, Morphogenesis, Protein analysis

## Abstract

Children born small for gestational age (SGA) face elevated risks of metabolic, cardiovascular, respiratory, and neurodevelopmental disorders, as well as premature mortality, yet the underlying mechanisms remain only partly understood. We analyze blood proteomic data from multiple birth cohorts to identify molecular pathways linked to SGA and to later-life lung function. We find that approximately one-third of SGA children exhibit a distinct molecular endotype marked by dysregulation of axon-guidance proteins in cord blood. In peripheral blood collected later in life, these proteins are inversely associated with contemporaneous spirometric restriction. Using GWAS data and an experimental sheep model, we obtain convergent evidence that axon-guidance genes are associated with spirometric indices (FEV_1_/FVC) at genome-wide significance and are broadly expressed during fetal development across multiple organs. These findings offer new insight into the developmental origins of chronic disease and highlight axon-guidance pathways as promising targets for investigating multiorgan morbidity.

## Abstract (web summary)

This multi-cohort study finds a third of Small for Gestational Age births show dysregulated axon guidance proteins in cord blood, linked to lower birth weight and reduced later lung function. The findings are strengthened with GWAS and sheep model data.

## Introduction

Newborns born small for gestational age (SGA), most often defined as birthweight lower than the 10th centile for their gestational age using a reference population^1,2^, are at increased risk for adverse health outcomes, including all-cause mortality^3^, learning difficulties, and chronic non-communicable diseases^1,4^. There is now substantial evidence indicating that SGA birth is associated with subsequent multiorgan morbidity, including increased risk for metabolic^5^, cardiovascular^6^, and neurodevelopmental diseases^7^. In support of this contention, we recently used lung function data from three birth cohorts and showed that SGA status was associated with a 3.3-fold increased risk of subsequent spirometric restriction, i.e., reduced forced vital capacity (FVC) with preserved forced expiratory volume in 1 s (FEV_1_)/FVC ratio up to adult life^8^. Spirometric restriction is an established risk factor for all-cause mortality and is specifically associated with a doubling of cardiovascular mortality risk^9–11^, suggesting that it may serve as a marker of multisystem involvement in individuals born SGA.

A study involving low- and middle-income countries estimated that, in 2010, almost 60% of all SGA infants were born at term, and reported that the incidence of term SGA reached up to 41% of all newborns in South Asia^12^. Sixteen million term children were born SGA in India, Pakistan, Nigeria, and Bangladesh alone^12^. This highlights the substantial global potential disease burden of SGA-related impaired lung function and all-cause mortality. Despite its potential massive effect on public health, the biological mechanisms that determine the association between SGA status and subsequent morbidity and mortality remain only partly understood. Most children born SGA have intrauterine growth restriction (IUGR), the pathogenesis of which is thought to be primarily mediated by impaired blood flow due to placental dysfunction. This results in the lack of delivery of oxygen and nutrients to the fetus, and associated changes in the expression of proteins required for fetal growth, such as insulin-like growth factors (IGF), transforming growth factor beta, leptin, and mTOR^13,14^. However, there is scant information on the molecular mechanisms that underlie these changes.

Here, we leveraged the Children’s Allergy and Asthma Data Repository (CADRE) network of birth cohort studies together with the SomaScan proteomics platform to elucidate the molecular mechanisms that are related to SGA status in cord blood and to subsequent lung function outcomes in later life, as a quantitative marker of multiorgan impairment. Our findings identify a molecular endotype of SGA that is found in multiple cohorts recruited from diverse environmental locations and genetic backgrounds.

## Results

The study was based on a two-stage design, nested within the CADRE consortium^15^ of birth cohorts collected from diverse US-based locations across several decades, including the Columbia Center for Children’s Environmental Health (CCCEH), Childhood Origins of Asthma (COAST), Infant Immune Study (IIS), Tucson Children’s Respiratory Study (TCRS), Wayne County Health, Environment, Allergy, and Asthma Longitudinal Study (WHEALS), and Wisconsin Infant Study Cohort (WISC) cohorts (Supplementary Table 1). The first stage entailed a case/control comparison of cord blood protein expression levels in SGA subjects versus Appropriate for Gestational Age (AGA) controls. The second stage focused on relating the SGA-associated proteins identified in cord blood with expression levels in peripheral blood and lung function outcomes. For the cord blood study, 99/207 (48%) of the subjects were SGA at birth, and 106/207 (51%) were assigned female at birth (Table 1), and this was representative of each cohort used in the study (Supplementary Table 2). As expected, individuals born SGA had lower weight, length, and head circumference at birth, and tended to have a shorter gestational age (Table 1).

**Table 1:** Relevant birth metrics of individuals with CBMC samples stratified by SGA status at birth

	Total (*n* = 207)	Those born AGA (*n* = 108)	Those born SGA (*n* = 99)	*p*-value^a^
**Sex (assigned female at birth) (%)**	106/207 (51.21%)	54/108 (50%)	52/99 (52.52%)	0.781
**Birth weight (grams)**^**b**^ **mean (95% CI); [min–max]**	3067.48 (2999.11–3135.85) [1146]	3445.35 (3377.89–3512.8) [2146]	2662.89 (2613.27–2,712.51) [1929–3062]	2.29 × 10^−31^
**birth length (cm)**^**c**^ **mean (95% CI); [min–max]**	49.31 (48.96–49.66) [43.18–55.37]	50.53 (50.1–50.96) [45.72–55.37]	47.84 (47.45–48.24) [43.18–53]	7.31 × 10^−14^
**Head circumference at birth (cm)**^**d**^ **mean (95% CI); [min–max]**	33.70 (33.46–33.93) [30.0–38.0]	34.53 (34.26–34.81) [31.5–38.0]	32.72 (32.47–32.97) [30.0–35.0]	2.83 × 10^−15^
**Gestational age (weeks) mean (95% CI); [min–max]**	39.29 (39.12–39.47) [35.0–42.0]	39.44 (39.21–39.67) [35–41.5]	39.13 (38.87–39.39) [35.5–42]	0.031

*SGA* Small for Gestational Age, *CI* Confidence Interval, *min* minimum, *max* maximum, *cm* centimeter.

^a^*p*-value calculated for difference in the variable between those individuals born SGA versus AGA. Fisher’s test *p*-value (two-sided) shown for categorical variables, and Mann–Whitney test *p*-value (two-sided) shown for continuous variables.

^b^Two individuals from the IIS cohort did not record this variable.

^c^Four individuals from the CCCEH, one sample from the IIS, and 17 samples from the TCRS cohorts did not record this variable.

^d^Nine individuals from CCCEH, 1 individual from IIS, 17 individuals from TCRS, and all 26 individuals from WISC did not record this variable.

### Identification of SGA-associated subgroups in cord blood

We generated proteomic profiles from 207 cord blood samples collected from five cohorts to investigate protein abundance patterns in relation to SGA. Following preprocessing and quality control, 7214 aptamers corresponding to 6536 unique proteins were available for analysis. Principal component analysis (PCA) of the cord blood proteomic profiles showed that the cohort of origin was the major source of variation among the first 10 components, which together accounted for 72% of the total variation (Supplementary Fig. 1).

We applied Limma^16^, in combination with Surrogate Variate Analysis (SVA)^17^ to correct latent sources of unwanted variation, to identify dysregulated proteins between SGA and AGA subjects in each individual cohort. The differential expression pattern was highly variable from cohort-to-cohort with respect to the signal magnitude, aptamer overlap, and enriched biological pathways (Supplementary Fig. 2). For example, in the TCRS and COAST cohorts, SGA status was related to differential expression of axon-guidance pathways, whereas in the CCCEH and IIS cohorts SGA was most strongly associated with immune pathways, although multivariate classification could still effectively discriminate between SGA and AGA status (Supplementary Fig. 2). This suggested the hypothesis that SGA is a complex and heterogeneous phenotype and by inference this heterogeneity may mask the identification of true signals that are restricted to a subgroup of individuals, that may represent distinct endotypes of SGA.

**Fig. 1:**
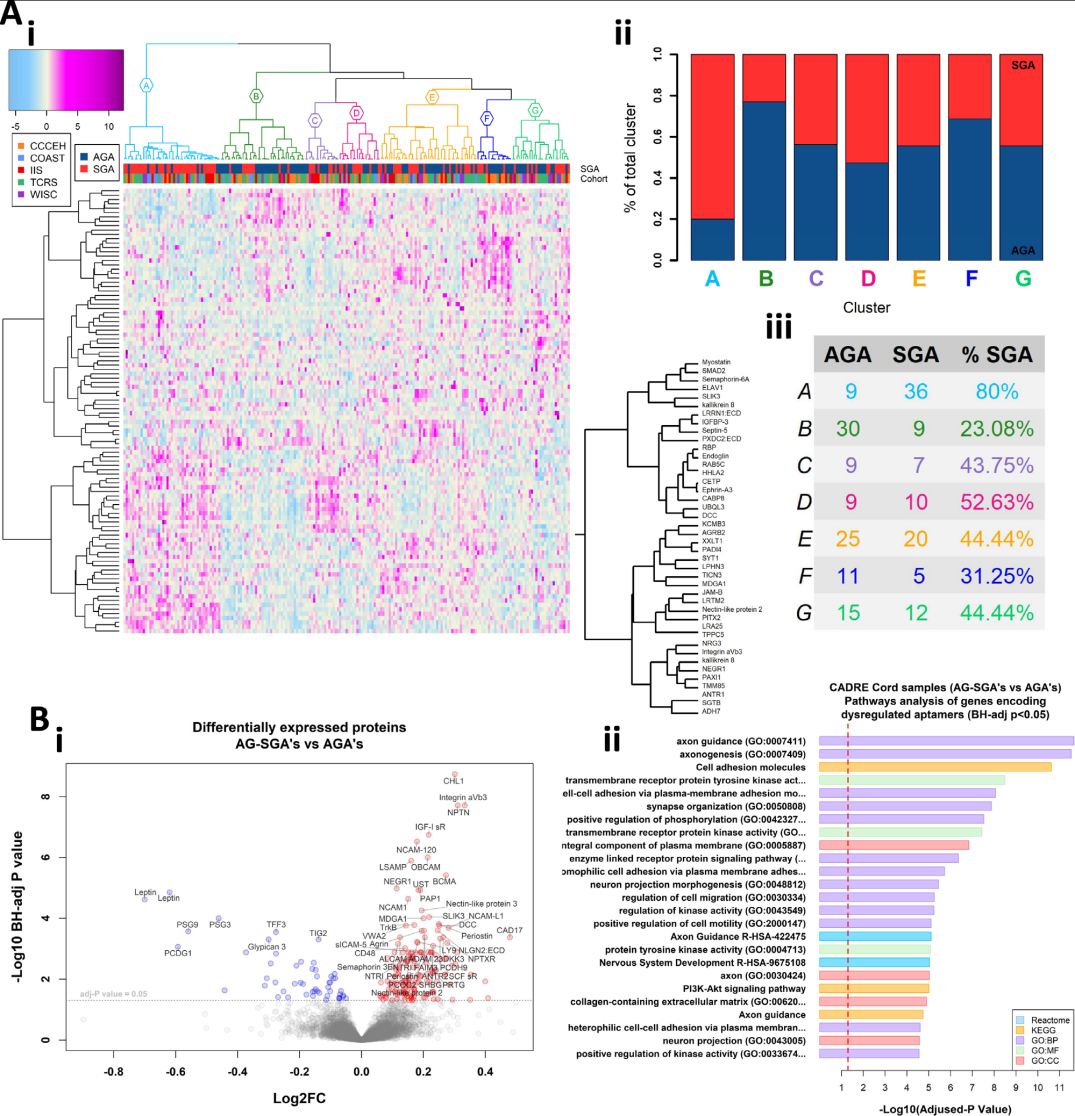
Consensus cluster analysis of aptamers identified with SGA-associated features selection. **A** **i** Heatmap illustrating the hierarchical consensus clustering results with samples (*n* = 207) in columns and aptamers (*n* = 102) in rows. Deeper pink color represents higher measurements and deeper blue color represents lower measurement, and this corresponds to the top element in the legend on the top right. The color bar across columns denotes each sample’s SGA status at birth and cohort, and this corresponds to the middle and bottom element of the legend. Dendrogram of samples is colored and label according to the consensus-defined clusters (**A**–**G**). Proportion of SGA in each cluster is displayed as a stacked bar plot (**ii**) and table (**iii**), with colors corresponding to the consensus-defined clusters. **B** **i** Volcano plot of the differential expression analysis (Limma) results for the cord blood comparison between AG-SGA (Cluster A SGA) and AGA individuals with adjustment for surrogate variables. The plot shows the Log_2_ fold change on the *x*-axis and the (−log_10_) Benjamini–Hochberg (BH)-adjusted *P*-value (two-sided) on the *y*-axis. The grey dashed line indicates a BH-adjusted *p*-value of 0.05 and points colored red (blue) represent aptamers considered to have significantly higher (lower) measurements in AG-SGA individuals. Protein names corresponding to the top 30 differentially expressed aptamers are displayed on the plot. **ii** Horizontal bar plot showing the top 25 significantly enriched pathways of the genes that encode significantly dysregulated aptamers from the KEGG, GO, and Reactome repositories. The *x*-axis shows the (−log_10_) adjusted *p*-value of the enrichment analysis and the *y*-axis displays the pathway terms. The results are order by decreasing adjusted *p*-value and colored according to the (sub)repository. The full results tables for the differential expression and pathways analysis are provided in the **Source Data** file.

To address this, we implemented a cluster analysis strategy to determine if the subjects born SGA form distinct subgroups. Briefly, we applied Partial Least Squares (PLS) as a supervised feature selection technique to each individual cohort to identify proteins related to SGA status, and merged proteins that were detected in at least three cohorts into a signature for consensus hierarchical clustering. As illustrated in Fig. 1A, seven clusters of subjects were identified. Cluster A was strongly enriched for SGA (36/45, 80%), and this cluster was characterized by upregulation of a distinct, 42-aptamer signature comprising axon guidance cues (e.g., Ephrin-A3, Semaphorin-6A, DCC) and growth factor signaling pathways (e.g., IGFBP-3, SMAD2). Whilst cluster A comprised 36% of the total number of subjects with SGA, the remaining 63 of the total 99 SGA samples (64%) were distributed amongst the other clusters which were not enriched for SGA (Fig. 1A and Supplementary Fig. 3A). Importantly, there was no observable effect of the cohort of origin on the clustering pattern (Fig. 1A and Supplementary Fig. 3B). Based on these data and for the purposes of this study, we refer to the SGA samples in Cluster A as axon guidance-associated (AG-SGA) and those with more heterogeneous profiles assigned to other clusters as non-axon guidance-associated (nonAG-SGA). Notably, the proportion of subjects classified as AG-SGA versus nonAG-SGA was remarkably consistent across all cohorts (range 33–40%) (Supplementary Table 3).

### AG-SGA is associated with prominent upregulation of axon guidance proteins

Comparison of AG-SGA with all AGA controls, adjusting for surrogate variables, showed that 221/7214 (3%) of the total aptamers were differentially expressed (Benjamini–Hochberg (BH)-adjusted *p* < 0.05), 169 (76%) of which were upregulated (Fig. 1B). Alongside dysregulation of leptin and IGF family proteins, AG-SGA exhibited substantial upregulation of aptamers corresponding to axon guidance proteins, based on Gene Ontology (GO), Kyoto Encyclopedia of Genes and Genomes (KEGG), and Reactome databases (Fig. 1B and Supplementary Fig. 3C, D). Aptamers corresponding to placenta/pregnancy-specific proteins were downregulated in AG-SGA, including Insulin-like 4/placentin (INSL4) and pregnancy-specific glycoproteins (PSG2, 3, and 9) (Fig. 1B and Supplementary Fig. 3E). Moreover, differential expression analysis of AG-SGA versus nonAG-SGA samples also found upregulation of axon guidance cues and downregulation of placenta/pregnancy-specific proteins (Supplementary Fig. 4). No aptamers recorded an FDR-adjusted *p*-value < 0.05 for the comparison between nonAG-SGA versus AGA, indicating that individuals with this SGA form may be indistinguishable from AGA status based on cord blood protein measurements. AG-SGA subjects had lower birth weight compared to nonAG-SGA (mean 2550 g vs 2727 g; *p* = 0.0036) but not birth length (mean, 48 cm vs 47 cm; *p* = 0.21), and there was no significant difference in sex between the groups (Supplementary Fig. 5). In this context it is noteworthy that maternal weight gain during pregnancy was half that in AG-SGA compared to nonAG-SGA individuals in the IIS cohort (8.4 kg vs 17.1 kg, *p* = 0.06). Taken together, these results suggest that a subset of individuals born SGA exhibit enhanced expression of axon guidance-related proteins, detectable in cord blood proteomic profiles, that may represent a distinct molecular endotype from other forms of SGA.

### Network analysis of cord blood-derived proteomic profiles

**Fig. 2:**
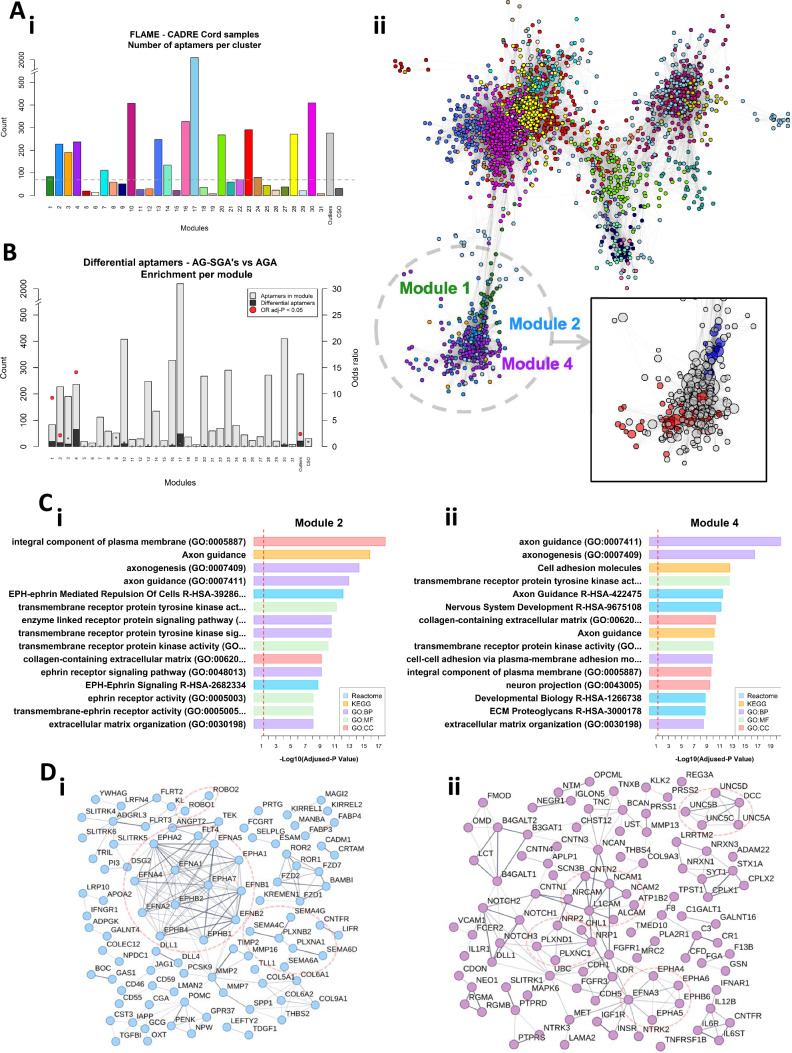
Network analysis of cord blood-derived proteomic profiles. **A** **i** Bar plot displaying number of aptamers (*y*-axis) per cluster identified by FLAME. *X*-axis shows the 31 clusters of functionally related aptamers (and outliers/CSOs). *Y*-axis denotes aptamer number (count) and double slash indicates an axis scale break to aid visualization. **ii** Representative (cosine) network wiring diagram demonstrating the intra- and inter-module connections amongst aptamers, colored by FLAME module (corresponding to bars in (**i**), which serves as a legend). (Insert) Section of the representative wiring diagram (indicated with dashed grey circle) focused on modules 1, 2, and 4 overlaid with differential expression analysis results for the comparison between AG-SGAs and AGAs. Nodes colored red are significantly upregulated and those colored blue are significantly downregulated. Node size corresponds to the absolute log_2_FC for the analysis. **B** Stacked bar plot displaying Fisher’s test results for over-representation of significant differentially expressed aptamers amongst modules. *X*-axis and left *y*-axis show the modules and number of aptamers (as in **A** (**ii**)), respectively. Black shading in each bar indicates the number of aptamers that were significantly dysregulated from the analysis of AG-SGA’s versus AGA for that module. Points correspond to the right *y*-axis and show the Fisher’s test odds ratio (full results table supplied in the **Source Data** file). Points colored red indicate those with a BH-adjusted *p*-value < 0.05 (two-sided) associated with the odds ratio (*p*-value adjustment for number of modules). The significant result for the outliers module suggests several aptamers dysregulated in AG-SGAs could not be assigned to a module. **C** Horizontal bar plots showing the top 15 significantly enriched pathways (KEGG, GO, and Reactome repositories) from genes encoding Module 2 (**i**) and 4 (**ii**) aptamers. *X*-axis shows the (−log_10_) Benjamini–Hochberg adjusted *p*-value (one-sided) and *y*-axis displays the pathway terms. Results ordered by decreasing adjusted *p*-value and colored by (sub)repository. **D** STRING Protein-Protein Interaction (PPI) networks for the aptamers/proteins modules 2 (**i**) and 4 (**ii**). Edges between proteins represent the confidence of the connection based on prior knowledge interactions. Dashed red circles indicate cliques of interest in the networks.

Proteins do not exist or function in isolation, they are expressed in the context of modules. We next employed the Fuzzy clustering by Local Approximation of Memberships (FLAME) algorithm^18^ as an unsupervised approach to identify functionally related protein co-expression modules. FLAME identified 31 discrete modules in the data with a median membership of 70 aptamers (Fig. 2A). Notably, three modules were enriched with proteins that were differentially expressed between AG-SGA versus AGA subjects; Module 1 (20/84 (23.81%); Odds Ratio (OR) 9.23 [95%CI = 5.19–15.82]; BH-adjusted *p* = 1.01 × 10^−10^), Module 2 (16/228 (7.02%), OR 2.13 [95%CI = 1.17–3.63]; BH-adjusted *p* = 0.037), and Module 4 (65/237 (27.43%), OR 14.12 [95%CI = 10.02–19.78]; BH-adjusted *p* = 1.48 × 10^−40^) (Fig. 2A, B). Similar results were observed using aptamers dysregulated from the comparison between AG-SGA and nonAG-SGA subjects, as expected from the overlap in the differentially expressed aptamers (Supplementary Fig. 6A). These modules formed a distinct region of the network topology (Fig. 2B), suggesting overlapping functions. Pathways enrichment analysis of all aptamers in Modules 2 and 4 showed they were strongly associated with axon guidance pathways, consistent with the overrepresentation of aptamers with significantly higher expression in AG-SGAs (Fig. 2A–C). In contrast, the aptamers in Module 1 were enriched in pathways including extracellular matrix, collagen, and IGF binding (Supplementary Fig. 6B).

To further characterize these modules, we created Protein-Protein Interaction (PPI) networks of the aptamers from each module member with STRING^19^. Prominent sub-networks of ephrin-related interactions were a feature of the PPIs for both module 2 and 4, alongside cliques of other axon guidance pathways, including semaphorin and plexin interactions in module 2, and UNC5, neuropilin, plexin and Cellular Adhesion Molecule interactions for module 4 (Fig. 2D). The Module 1 PPI exhibited prominent interactions between collagen proteins aligning with the corresponding pathways analysis results (Supplementary Fig. 6C).

We next calculated module eigenvectors to summarize the major source of variation and assess the relationship between these modules and birth measurements. The module 4 eigenvector was significantly associated with birth weight, length, and head circumference, but not gestational age (Supplementary Fig. 7). In contrast, the module 2 eigenvector had a weak correlation with birth weight and was strongly correlated with gestational age. Finally, we applied FLAME analysis to the cord blood profiles of each cohort individually and identified specific axon guidance-associated modules in each cohort (Supplementary Figs. 8 and 9), confirming that their presence is a general feature of cord blood profiles from individual recruited from diverse environmental locations and genetic backgrounds.

### Relation of axon guidance proteins in peripheral blood with lung function outcomes

**Fig. 3:**
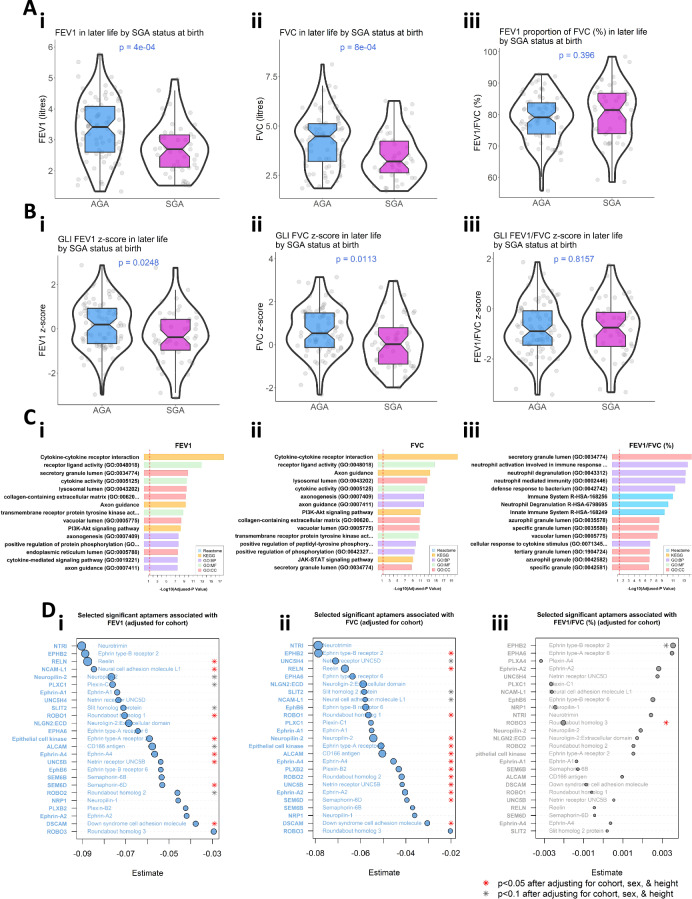
Analysis of SGA status and proteomic profiles generated from blood samples collected in later life. **A** Violin plots of FEV_1_ (**i**), FVC (**ii**), and FEV_1_ percentage of FVC (%) (**iii**) stratified by SGA status at birth for individuals from the TCRS, IIS, and WHEAL cohorts with later life blood samples. The violins visualize the distributions, and the boxplots show the median and interquartile range (IQR) with whiskers extending to the most extreme values within 1.5 × IQR below and above the 1st and 3rd quartiles, respectively. Points represent individuals (80 AGA and 46 SGA subjects). The (nominal) Mann–Whitney *p*-value (two-sided) corresponding to the pairwise comparison in each plot is shown (blue text). **B** Violin plots of FEV_1_ (**i**), FVC (**ii**), and FEV_1_ percentage of FVC (%) (**iii**) z-scores calculated with the GLI reference equations stratified by SGA status at birth for individuals from the TCRS, IIS, and WHEAL cohorts. Plot parameters are the same as those in (**A**) and show Mann–Whitney *p*-values (two-sided). **C** Horizontal bar plot showing the top 15 significantly enriched pathways from the KEGG, GO, and Reactome repositories of genes encoding aptamers significantly negatively associated with FEV_1_ (**i**), FVC (**ii**), and FEV_1_ percentage of FVC (%) (**iii**) in later life. The *x*-axis shows the (−log_10_) Benjamini–Hochberg adjusted *p*-value (one-sided) of the enrichment analysis and the *y*-axis displays the pathway terms. The results are order by decreasing adjusted *p*-value and colored according to the (sub)repository. **D** Plots showing linear model results for 25 selected axon guidance-related aptamers for FEV_1_ (**i**), FVC (**ii**), and percentage of FEV_1_ of FVC (%) (**iii**) comparisons. Points are colored red/blue depending on the significant positive/negative association (two-sided *p*-value < 0.05), non-significant results are colored grey. Red/grey asterisks on the right of the plots show results that recorded a *p*-value < 0.05/ < 0.1 (unadjusted) after also adjusting for sex and height. These plots demonstrate that many axon guidance-related proteins associated with SGA status at birth may also be associated with lung function restriction later in life. Results tables for pathways enrichment and linear model analysis are provided in the **Source Data** file.

Protein profiles were generated from peripheral blood samples collected during childhood (IIS; 8–13 yrs (*n* = 19), WHEALS; 11–13 yrs (*n* = 32)) and adulthood (TCRS; 36 yrs (*n* = 75)) with corresponding lung function measurements assessed with spirometry (Supplementary Table 4). We observed strong positive correlation (Spearman’s rho = 0.904, *p* < 2.2 × 10^−16^) in aptamer expression between cord blood-derived and later life peripheral blood-derived protein profiles for the 12/126 (9.52%) of these individuals data at both collection time points (Supplementary Fig. 10A). For individuals with later life protein profiles, those born SGA exhibited reduced height on average (*p* = 0.011), but not weight or BMI (Supplementary Fig. 10B). Notably, individuals born SGA had reduced FEV_1_ and FVC, but not the percentage of FVC (%FEV_1_/FVC), compared to those born AGA, and this was most noticeable in the adult samples (Fig. 3A and Supplementary Fig. 10C). As we recorded significant difference in height between individuals born SGA, we calculated FEV_1_, FVC, and FEV_1_/FVC ratio z-scores with the Global Lung Function Initiative (GLI) reference equations^20,21^, which employ a large reference population to remove bias related to age, sex, and height. Analysis of these z-scores confirmed that individuals born SGA had reduced FEV_1_ and FVC, but not the FEV_1_/FVC ratio, irrespective of age, sex, and height (Fig. 3B).

To identify individual aptamers associated with FEV_1_, FVC, and %FEV_1_/FVC, we applied linear modelling adjusting for cohort (and thus age). The results showed similar aptamer profiles are significantly associated with FEV_1_ and FVC, but not %FEV_1_/FVC (Supplementary Fig. 10D, E). Importantly, axon guidance pathways were significantly enriched by genes encoding aptamers that were negatively associated with FEV_1_ and FVC, and many aptamers corresponding to axon guidance-related proteins, such as Ephrin, Plexin, and Semaphorin family members, were negatively associated with FEV1 and FVC, but not %FEV_1_/FVC (Fig. 3C, D, Supplementary data). Many of the aptamers that correlated with FEV_1_ and FVC remained significant after also adjusting for sex and height (Fig. 3D and Supplementary Fig. 11A). Further, we applied elastic net regression to the childhood/adult protein profiles to assess lung function measurement prediction as a multivariate approach (with cohort, sex, and height as covariates). The results demonstrate moderate-to-good prediction of FEV_1_ and FVC, but not %FEV_1_/FVC, and several axon guidance-related proteins were among the top predictive coefficients (e.g., SPON2, CHL1, DCC) (Supplementary Fig. 11B, C). Additionally, restricting the input aptamers to those dysregulated from the AG-SGA versus AGA analysis in cord blood (*n* = 221), demonstrated similar prediction for FEV_1_ and FVC, but not %FEV_1_/FVC, driven by axon guidance-related proteins (Supplementary Fig. 11D, E). We note that the age of subjects from the three cohorts does not capture the full continuum between childhood and adulthood, and this is a limitation of the analysis.

### Axon guidance proteins are GWAS candidates for FEV_1_ and FVC

**Fig. 4:**
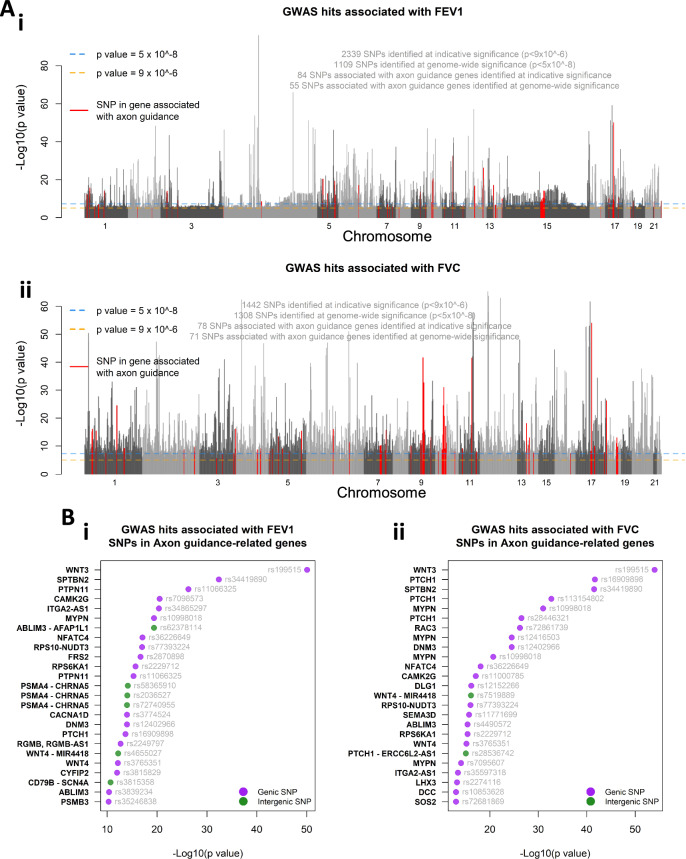
Meta-analysis of SNPs associated with FEV_1_ and FVC. **A** Bar plot of −Log_10_(*p*-values) of the recorded GWAS SNP association with FEV_1_ (**i**) and FVC (**ii**). The *y*-axis shows the two-sided *p*-value for the association and the *x*-axis is order by chromosome regions, with alternating shade of grey indicating the chromosome where each SNP is located (odd numbered chromosome labelled). SNPs colored red are those associated with axon guidance-related genes; the cluster in chromosome 15 in (**i**) corresponds to multiple SNPs in *PSMA4*, and clusters in chromosome 9 and 10 in (**ii**) correspond to multiple SNPs in *PTCH1* and *MYPN*/*UNC5B*/*CAMK2G*, respectively. The dashed orange line indicates the minimum two-sided *p*-value threshold output from GWAS catalog (*p*-value = 9 × 10^−6^) and the dashed blue line represents the genome-wide significance threshold (two-sided *p*-value = 5 × 10^−8^, corresponding to a Bonferroni correction for approximately one million independent common variants tested genome-wide). **B** Plots of the top 25 SNPs significantly associated with axon guidance-related genes for FEV_1_ (**i**) and FVC (**ii**). The *x*-axis shows the −Log_10_(*p*-value) (two-sided) of the association and the *y*-axis shows the mapped gene(s). Points are colored according whether they are genic or intergenic SNPs and the SNP identifier is shown in grey text alongside each point.

To further evaluate the relationship between axon guidance and FEV_1_ and FVC, we queried the NHGRI-EBI Catalog of human genome-wide association studies (GWAS Catalog)^22^ to identify variants associated with these spirometry measurements, highlighting those linked to genes from axon guidance pathway terms from the KEGG, GO, and Reactome repositories. This approach identified 1109 SNPs associated with FEV_1_ at genome-wide significance (*p*-value < 5 × 10^−8^) from 50 studies (catalog accession codes), of which 55 (4.96%) were in regions mapped to axon guidance-related genes (Fig. 4A, B and Supplementary Fig. 12A). Similarly, 1308 SNPs were associated with FVC (*p*-value < 5 × 10^−8^) from 33 studies, with 71 (5.43%) SNPs linked to axon guidance-related genes. Fifteen of the genome-wide significant SNPs that mapped to axon guidance-related genes were associated with both FEV_1_ and FVC, and this was greater than expected by chance (*p* = 1.325 × 10^−14^, OR = 25.59 [95%CI range 12.26–51.42]) (Supplementary Fig. 12B). This demonstrates that genetic variation in regions harboring axon guidance genes are known markers influencing FEV_1_ and FVC measurements.

### Expression of axon guidance cues across multiple organs during fetal development in a sheep model of fetal growth restriction (FGR)

**Fig. 5:**
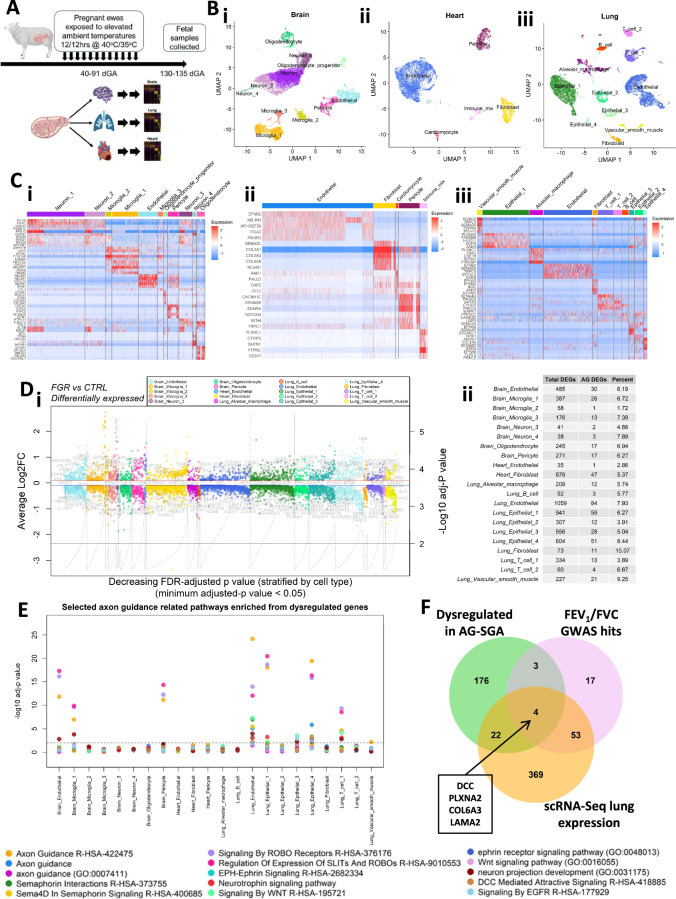
Single cell RNA-Seq analysis of multiple fetal sheep organs. **A** Overview of sheep model. Pregnant ewes were exposed to elevated ambient temperatures between 40 and 91 (± 1) dGA to induce FGR and fetal samples were collected between 130–135 dGA. Created in BioRender. Bosco, A. (2026) https://BioRender.com/kalaxrb. **B** UMAP plots of cells from fetal brain (**i**), heart (**ii**), and lung (**iii**) tissue, colored and labeled by cell type. Axes show the first two UMAP variables, respectively. **C** Heatmap of top axon guidance-related genes by cell type for the fetal brain (**i**), heart (**ii**), and lung (**iii**) tissue. *X*-axis shows cells, stratified by cell type, and *y*-axis shows genes. Gene expression colored red and blue indicate higher and lower expression, respectively. **D** **i** Combined volcano plot showing differentially expressed genes between FGR and control. *X*-axis shows the two-sided (Benjamini–Hochberg) FDR-adjusted *p*-value (*p* = 0.05 minimum) and left *y*-axis shows the average log_2_FC. The points are colored according to cell type/organ and the red/blue horizontal lines denote an average log_2_FC of 0.1/−0.1. Selected axon guidance-related genes are shown in the plot in grey text. The right y-axis shows the (−log_10_) FDR-adjusted *p*-values (two-sided, Benjamini–Hochberg) and corresponds to the dashed grey line. **ii** Table showing the number of DEGs, number of axon guidance-related DEGs and percent of axon guidance DEGs amongst total DEGs for each cell type/organ. **E** Pathways enrichment analysis results for 15 axon guidance-related pathway terms from differentially expressed genes (FGR vs control) for each cell type/organ. *X*-axis shows the cell type/organ and *y*-axis shows the (−log_10_) Benjamini–Hochberg adjusted one-sided enrichment *p*-value. The dashed grey line indicates an adjusted *p*-value of 0.01. Points are colored by pathways terms, shown in the legend below. **F** Venn diagram showing overlap of genes encoding proteins dysregulated in AG-SGA samples (green, see Fig. 1B), axon guidance-related genes that map to SNPs associated with FEV_1_ and/or FVC at genome-wide significance (*p* < 5 × 10^8^) (pink, see Fig. 4A), and axon guidance-related genes expressed in at least 1% of cells from mammalian (sheep) fetal lung samples (orange). The four intersect genes are shown in the box insert.

We employed an experimental sheep model of heat stress-induced FGR to determine whether axon guidance proteins are expressed in multiple organs during fetal development. Pregnant ewes were exposed to elevated ambient temperatures between 40 and 91 (± 1) days gestation age (dGA) to induce FGR and we collected fetal brain, heart, and lung samples at 130–135 dGA for scRNA-Seq profiling (Fig. 5A). Following preprocessing and QC, there were 47,420 cells available for analysis representing 27 cell types from the three organs (Fig. 5B and Supplementary Fig. 13). Focusing our analysis on 563 genes present that correspond to human axon guidance-related genes, we found strikingly varied expression patterns across fetal tissues and cell types (Fig. 5C). These included genes encoding members of the major axon guidance cues and their receptors, such as ephrins, semaphorins, netrins, ROBO, and plexins, confirming their expression in a range of cell types and organs during late mammalian fetal development. We applied MAST^23^ (with sheep/donor modeled as a random effect) to determine significantly dysregulated genes between FGR and control samples. Axon guidance-related genes featured among DEGs for all cell types assessed (Fig. 5D). Additionally, axon guidance-related pathways were significantly enriched from these DEGs, most prominently Axon Guidance and SLIT/ROBO pathways from the Reactome repository from endothelial and epithelial cells from the lung and endothelial and pericytes from the brain (Fig. 5E). We note that these findings were generated from a small exploratory study model, and thus we treat these results as preliminary.

Finally, we probed the overlap between the proteomics data, GWAS data, and sheep scRNA-Seq data to identify AG-SGA associated genes/proteins expressed during fetal lung development that have persistent influence on lung function in later life. We focused on the overlap between proteins associated with AG-SGA status in cord blood, GWAS hits for FEV_1_ and/or FVC, and genes expressed in ovine fetal lung. This approach identified four candidate genes/proteins; the netrin receptor DCC, the semaphorin receptor Plexin A1, the alpha 3 chain of type VI collagen (*COL6A3*), and Laminin subunit alpha 2 (*LAMA2*) (Fig. 5F).

## Discussion

Children who are born SGA are at increased risk for adverse health outcomes, including all-cause mortality and the subsequent development of chronic diseases that affect the respiratory, cardiovascular, metabolic and nervous systems, but little is known about the underlying biological mechanisms involved. Here, we provide evidence that one-third of children born SGA show dysregulation of axon guidance proteins, a major molecular system known to be involved in the development of a broad array of tissues and organ systems across phylogeny^24^. Based on our recent finding that SGA status is associated with spirometric restriction in adulthood^8^, we assessed the association between lung function and the expression of axon guidance proteins in peripheral blood and found that the latter was inversely related to FEV_1_ and FVC up to adult life, demonstrating that these pathways may play a role in the impaired lung growth observed in SGA. We obtained orthogonal evidence in support of this contention from the GWAS catalogue, demonstrating that polymorphisms that map to axon guidance cues are associated with FEV_1_ and/or FVC at genome-wide significance. Finally, we showed that axon guidance cues are expressed in the brain, lung, and heart during fetal development in the context of FGR in a sheep model. Intersecting proteomic, GWAS, and ovine fetal lung data, we identified four candidate genes/proteins (DCC netrin 1 receptor, plexin A1, collagen type VI alpha 3 chain, laminin subunit alpha 2) that are dysregulated in early life, are GWAS hits influencing FEV_1_ and/or FVC traits, and expressed during fetal lung development. Together, our findings identify a biological mechanism that could at least partially explain the multiorgan morbidity and impaired physiological function observed in SGA.

All four major families of canonical axon guidance proteins (ephrins, semaphorins, netrins, SLITs) were overexpressed in cord blood in the subgroup of children showing an alteration in this molecular system in our population sample. Axon guidance molecules and their receptors were first described as playing a fundamental role in regulating connectivity in the developing nervous system^25^. The process is mediated by guidance cues, which are secreted into the extracellular environment and guide neurons to their targets by acting as attractants or repellents (push/pull mechanism). Whilst axon guidance cues were originally characterized in the brain, they are also expressed outside the brain, where it has been postulated that they play a role in the early stages of branching morphogenesis^26^, the developmental process by which many organs, including the lung and vascular system, generate tree-like structures. Evidence from lung bud organ cultures and three-dimensional lung epithelial culture systems have demonstrated a role for semaphorins and netrins in regulating morphogenic responses^27,28^. Moreover, mice that are deficient in EphrinB ligands and receptors die *in utero*, due to defective formation and morphogenesis of the vascular architecture^29^. However, if signals by single axon guidance molecules promote or inhibit vascular morphogenesis in specific tissues is still unknown^30,31^. Taken together, these findings suggest that axon guidance cues are key regulators of morphogenic responses in vascular systems and, by this mechanism, in a variety of tissues and organs^24^.

We reasoned that, if axon guidance played a major role in lung morphogenesis, confirmatory signals would be present in genetic studies of lung function. Indices derived from spirometric tests have been richly profiled with GWAS, and we used publicly available data to show that polymorphisms in axon guidance-related genes were commonly reported to be associated with FEV_1_ and FVC. Moreover, Shrine et al.^32^ performed a GWAS meta-analysis of lung function comprising >580,000 participants and identified a set of 559 putative causal genes, including those encoding Neurotrimin, Semaphorin 3 A, Netrin receptors (*DCC*, *UNC5B*), and Neuropilin 2, among others. Furthermore, their analysis identified “Axon guidance” (adj-*p*-value = 0.0016; ConsensusPathDB/Reactome) and “Axonal guidance signaling” (*p*-value = 2.51 × 10^−15^; Ingenuity Pathways Analysis) as pathways associated with the putative causal genes. It is thus plausible that the role of axon guidance cues in lung development may not be limited to individuals born SGA. Precisely how axon guidance dysregulation in fetal lung development may persist to influence respiratory function in later life is unknown, although epigenetic mechanisms are presumably involved^33^.

We used an exploratory experimental sheep model to assess the expression of axon guidance cues during early fetal development in the context of FGR at single cell resolution. We observed expression of members of the major axon guidance cues and their receptors in the lung, including semaphorins/plexins, Ephrin, ROBO, and netrin, among others, and these demonstrated differing expression levels between cell types. Overall, these data highlight that a subset of axon guidance cues manifest specific expression patterns in organs with specialized cell types (e.g., lung epithelial and AT2 cells) and more general conserved patterns in cell populations that are shared across organs. Additionally, although only a small number of animals were available for analysis, our preliminary findings show that axon guidance-related genes and pathways may be commonly dysregulated in multiple cell types from FGR sheep compared to control, although larger studies are required to determine the extent to which this may be related to altered development of different organs and multiorgan comorbidity.

By overlapping the proteomics data, GWAS data, and ovine fetal lung expression data, we identified four candidate genes/proteins (DCC netrin 1 receptor, plexin A1, collagen type VI alpha 3 chain, laminin subunit alpha 2) that potentially contribute to the link between SGA status at birth and subsequent abnormal lung development and impaired physiological function. DCC (and UNC5B) are receptors for netrin 1, which is thought to play a role in lung branching through the regulation of morphogenic responses induced by fibroblast growth factor stimulation of lung endoderm^28^. Plexin A1 functions as the primary receptor for Semaphorin 3 A, which in turn is a regulator of lung branching morphogenesis^27,34^. Collagen type 6 is a structural component of the extracellular matrix and is essential for normal lung structure and function^35^. Laminins are expressed in the basement membrane and are essential for normal lung development^36^. Laminin alpha 1 is important for branching morphogenesis and laminin alpha 2 is important for smooth muscle differentiation^36^. Moreover, thickening of the basement membrane has been observed in children who go on to develop asthma^37^, and low birth weight individuals are at increased risk for the development of the disease^38^. Together, our data, in combination with these previous studies, highlight important roles for these four candidates in lung development and function.

The risk factors that give rise to axon guidance-associated SGA merit further investigation. AG-SGA represented approximately one-third of all SGA subjects, and this proportion was remarkably consistent across cohorts, suggesting it is a general feature of SGA that is robust to large variations in environmental locations and genetic backgrounds. AG-SGA newborns had significantly lower birth weights on average than nonAG-SGA, suggesting the former may represent a more severe form of SGA. Additionally, AG-SGA individuals exhibited downregulation of placenta-specific proteins compared to nonAG-SGA and AGA individuals, indicating placental dysfunction may play a prominent role in this group. Whilst SGA may be driven by several factors, including placental dysfunction, (epi)genetics, and maternal lifestyle^39^, we note that mothers of AG-SGA children within the IIS cohort failed to gain weight on par with their nonAG-SGA and AGA counterparts, providing a clue that maternal factors may be an important driver of poor fetal growth and nutrition in AG-SGA.

Our study has limitations which should be acknowledged. Notable among these are the lack of a comprehensive set of harmonized clinical variables that capture risk factors during pregnancy across all cohorts, and unrecorded sources of variation associated with the collection of cord blood samples (e.g., duration of labor and time between delivery, cord blood clamping, and sample processing). Moreover, our results describe mostly associations and, therefore, mechanistic studies will be necessary to demonstrate causality. Further, we generated data at the end of pregnancy and as such did not capture the putative mechanisms during earlier time points *in utero*. The cohorts used in this study were collected across several decades employing different collection protocols, undoubtably contributing to the substantial variation in proteomic profiles observed between cohorts (particularly TCRS). Whilst the influence of this variation could be effectively removed with surrogate variables, purpose-designed studies to confirm and extend these findings would benefit from comprehensive consideration of all anticipated sources of unwanted variation in their design. Additionally, our study did not detect a consistent molecular pattern for nonAG-SGA status that can distinguish them from AGA status, and no substantial differences in the recorded clinical variables compared to AGA. Future studies investigating maternal genetics with comprehensive clinical variables recorded during pregnancy (such as nutrition, ultrasound biometric, placental function, and lifestyle factors) may elucidate the potential genetic and environmental factors precipitating nonAG-SGA status. Also, only 12 individuals had samples available in both the birth and childhood/adult timepoints, and as such it was not possible to meaningfully analyze the data longitudinally in a paired manner, nor was it possible to faithfully designate AG-SGA or nonAG-SGA endotype status to individuals with only childhood/adult samples. Finally, the study was performed in children born in the United States, and the applicability of our findings to populations with much higher incidence of SGA will require confirmatory studies. Despite this limitation, it was a strength of this study that AG-SGA status was stably identified in birth cohorts diverse in terms of geography, race and ethnicity, and decade of birth, suggesting that our results may be generalized to other populations.

In summary, our results suggest that alterations in the developmental mechanisms regulated by axon guidance proteins may play a critical role in the link between being born SGA and impaired lung growth. Further investigation is warranted to determine the extent to which these mechanisms may be involved in the multiorgan morbidity and increased risk for premature mortality observed in individuals born SGA.

## Methods

### Ethics statement

The local institutional review board at each participating site approved the study protocol. These were the University of Arizona IRB for the TCRS and IIS cohorts, and sheep study; the University of Wisconsin–Madison Health Sciences IRB for the COAST and WISC cohorts; the Columbia University IRB for the CCCEH cohort; and the Henry Ford Health IRB for the WHEALS cohort. Written informed consent or parent/guardian permission was obtained along with child assent as appropriate for participation in specific cohorts and the Children’s Respiratory and Environmental Workgroup (CREW) protocol. Consent was obtained from participants, parents, or legal guardians for the publication of de-identified data in the public domain. All participating institutions approved the data sharing procedures specified in the CREW and/or CADRE consortium agreements. Animal protocols were approved by the Institutional Animal Care and Use Committee at the University of Arizona. All experiment followed the guidelines set by the US National Research Council’s “Guide for The Care and Use of Laboratory Animals” and the US Public Health Service’s “Policy on Humane Care and Use of Laboratory Animals.” All experiments were performed at the University of Arizona Agricultural Research Center. This center is accredited by the Association for Assessment and Accreditation of Laboratory Animal Care International. Experimental details are reported in compliance with the animal Research: Reporting of In Vivo experiments guidelines (doi:10.1371/journal.pbio.3000411).

### Study subjects

The samples used in this study were curated from cohorts in the recently established CADRE, previously referred to as The CREW consortium^15^; a resource to pool and harmonize data from 13 allergy and asthma birth cohort studies in the US. Six of these cohorts are utilized in the present study.

The CCCEH cohort includes a total of 727 enrollments from prenatal clinics located in three New York City neighborhoods in Northern Manhattan and the South Bronx. This cohort was established to investigate the relationship between exposure to environmental toxicants and development of childhood illnesses^40^.

The COAST study is a birth cohort study that enrolled 289 children at high risk of developing asthma. This cohort was established to determine the role of immune response, lower respiratory infections, and gene-environment interactions in childhood asthma pathogenesis^41^.

The IIS is a population-based non-selected birth cohort that enrolled 482 mothers in the third trimester of pregnancy and followed the infants through 9 years of age^42^. The goal of IIS was to explore early and prenatal immune markers in the early origins of asthma.

The TCRS is a population-based non-selected birth cohort that enrolled 1246 healthy infants at birth between 1980 and 1984^43^. TCRS was designed to study respiratory disease risk factors and outcomes. Participants underwent in depth assessments into the fifth decade of life, which included measurement of lung function measurements and collection of serum or plasma.

The WHEALS cohort is a diverse, general-risk birth cohort with 1,258 enrollments from the metropolitan Detroit area. The goal of WHEALS is to link early life environmental factors with potential biological markers associated with childhood allergy and asthma risk^44^.

The WISC enrolled >200 families from rural farm and rural non-farm homes in Wisconsin. The goal of WISC is to investigate the association of early life exposure to animal farm environments with innate immune cell development, allergic sensitization, and reduced respiratory viral illness during infancy^45^.

In the present study, samples with available aliquots of cord blood or later life peripheral blood samples were included from the CCCEH (*n* = 43 cord samples), COAST (*n* = 20 cord samples), IIS (*n* = 40 cord and 19 8 yr samples), TCRS (*n* = 78 cord & 75 36 yr samples), WHEALS (*n* = 32 11–13 yr samples), and WISC (*n* = 26 cord samples) cohorts. SGA status was defined as birthweight lower than the 10th percentile and Average for Gestational Age (AGA) was considered birthweight within the 10th and 90th percentiles. All individuals assessed in this study (SGA and AGA) were born at term (i.e., no participants had preterm births). Sample were selected from each cohort so that there was an approximately even representation of SGA/AGA status and female/male subjects. The 126 individuals from the IIS, TCRS, and WHEALS cohorts used for later life analysis were selected as they had a peripheral blood aliquot available and had spirometry performed. Only 12 of the 126 (9.52%) individuals with a later life sample also had a samples in the cord blood data set. As such, it was not possible to conduct comprehensive paired analysis between the linked samples or retrospectively assign AG-SGA versus nonAG-SGA status to the later life samples. Supplementary method Table 1 shows information of these 12 individuals.

### Data harmonization

The CREW consortium within the ECHO program^15^ was established in 2016. In 2023, CREW transitioned into the CADRE and continued to process data. Clinical data from 5 of the 12 longitudinal birth cohorts was used. The cohorts collected similar information from participant surveys using differently worded questions at various ages for different recall and follow-up periods, depending on each cohort’s protocol. Investigators and the data coordinating team at the University of Wisconsin developed harmonized variable definitions consistent across all cohorts for parental atopy using an iterative process. The data coordinating team then created the final harmonized parental atopy variables, which the cohort data managers verified. For all other variables, investigators developed harmonized definitions consistent across all cohorts, and then cohort data managers created and verified the final harmonized variables.

### Proteomic data generation

Proteomic data was generated with the SomaScan platform (SomaLogic, Boulder, CO, USA)^46^, which allows high-throughput, simultaneous quantification of thousands of proteins. The fluorescence-based detection of aptamer abundance is reported as Relative Fluorescent Units (RFU), which is proportional to the concentration in the sample. The term “aptamer” refers to the short, single-stranded oligonucleotides that bind to target proteins with high affinity. Before data generation, the sample order was randomized and checked for even distribution with respect to SGA status and sex to ensure there was no inadvertent grouping by SGA/sex in the sequence order. The data received the following standardization/normalization as part of the data generation process: (1) Hybridization control normalization (to mitigate variation within the run that comes from the readout steps), (2) Median signal normalization (pooled calibrator replicates within run to mitigate technical variation in the calibrator signal), (3) Plate scale (adjusts for overall signal intensity differences between runs), (4) Calibration scale (adjusts for aptamer reagent-specific assay differences between runs), and (5) Median normalization (final sample normalization). The cord and adult data from the TCRS cohort were generated first, with expansion to include cord blood data from the CCCEH, COAST, IIS, and WISC cohorts and childhood data from the COAST, IIS, WHEALS, and WISC cohorts. The childhood data generated from the COAST and WISC cohorts were ultimately excluded from analysis due to missing spirometry data (COAST) and small sample size, (WISC, *n* = 3). The data was generated using the same methodologies, platform, and chemistry, ultimately profiling the same set of aptamers. A notable difference in the sample preparation was that TCRS samples collected as serum and the remaining CADRE cohort samples were collected as plasma. Supplementary method Fig. 1 shows that the data generated from serum and plasma samples is strongly positively correlated, which we believe justifies their joint analysis. Additionally, this potential technical effect would be accounted for with batch correction/surrogate variable adjustment.

### Quality control

All subsequent data QC and analysis was conducted in the R (version 4.3.2) environment unless otherwise stated. The SomaScan data.adat files for cord and later life sample were imported into R and assessed with the same QC pipeline. Sample RFU and log10(RFU) distribution were visualized with boxplots to detect any aberrant samples. Aptamers other than those corresponding to human proteins were excluded, which were those annotated Hybridization Control Elution, Non-Biotin, Non-Cleavable, Non-Human, Spurimer, and Spuriomer. Aptamer expression was assessed with respect to buffer samples and removed if they had an average expression difference of less than 100 RFU and/or a ratio less than 1.5. This step ensured a gap between aptamer expression and baseline buffer expression and ultimately excluded few aptamers. Outlier samples were assessed with a preliminary PCA to visualize separation from the buffer, calibrator, and pooled QC samples. The QC and calibrator samples were assessed to confirm even expression across the sequence order, thus confirming appropriate normalization steps were effective. Buffer, calibrator, and QC samples were then excluded from further quality control. As mentioned above, multiple aptamers can correspond to the same target protein. To assess the overlap of these aptamers, PCA was run on all aptamers and the first two PC were plotted with linkage between aptamers that encode the same target protein (Supplementary Method Fig. 2). This strategy showed that linked aptamers do not occupy the same principal component space, indicating they represent different aspects (e.g., conformations, subtypes) of the same target protein. Thus, all aptamers with the same protein were retained for analysis (rather than averaged or summed), and analysis was conducted at the aptamer level. For this reason, some analyses show a target protein multiple times, as expected from closely related aptamers corresponding to the same target protein. Aptamer density distributions were also assessed for outlier aptamer expression. Density distribution curves, Relative Log Expression plots, and Cumulative Distribution Function plots were generated for each sample, stratified by relevant technical and biological variables (e.g., assay plate, sex), to assess outlier samples. Additionally, PCA was used again to detect outliers (without buffer, calibrator, and QC samples), and the top 10 PCs were to assess to determine whether they capture variation attributable to any known variables (e.g., Sex). An example QC report is available on the Dryad repository. Following pre-processing and QC, there were 207 Cord blood proteomic profiles with 7214 aptamers corresponding to 6536 unique proteins and 139 later life (pediatric/adult) samples with 7154 aptamers corresponding to 6480 unique proteins.

### Principal component analysis

The log(RFU) data was used as input for PCA with the *PCA* function from the FactoMineR package. The input data was scaled within the function and the top 10 PC were calculated. The eigenvector (1st PC), which captures the greatest proportion of the variation in the data, was used as a summary metric of the source of variation. The cohort of origin was the major source of variation for the cord blood proteomics data (shown in Supplementary Fig. 1) and was also the greatest source of variation in the later life peripheral blood samples (Supplementary Methods Fig. 3).

### Differential expression analysis

Linear Models for Microarray Analysis (Limma)^16^ was developed for microarray experiments and employs empirical Bayes methods to shrink variance estimates towards a global value to improve statistical power for high-dimensional data (i.e., many more features than arrays) and thus is suitable for SomaScan technology. Significantly dysregulated aptamers were identified with the standard limma pipeline with default parameters. Briefly; a design matrix was constructed with the *model.matrix* function from the stats package, linear models were fit for each aptamer with the *lmFit* function, empirical Bayes moderation was applied with the *eBayes* function, and output metric were tabulated with the topTable function. For input, the RFU data was log-transformed and analysis was run for SGA status accounting for surrogate variables (see below), and SGA sub-cluster (endotype) status accounting for surrogate variables. Results tables are supplied in the **Source Data** accompanying this publication.

### Pathways analysis

The Enrichr database^47^, which comprises >500,000 pathways terms from ~230 libraries, was used to assign known biological pathways to input gene sets via the enrichR package. A set of gene symbols was the input for analysis and the Kyoto Encyclopedia of Genes and Genomes^48^ (KEGG; “KEGG_2021_Human”), Reactome^49^ (“Reactome_2022”), and Gene Ontology^50^ (GO; “GO_Biological_Process_2021”, “GO_Cellular_Component_2021”, “GO_Molecular_Function_2021”) libraries were used to identify significantly enriched pathways. The queried libraries were selected as they are well-established repositories that represent a broad range of relevant biology. As this analysis relied on the genes which encode the target aptamers, and the assay does not cover the entire products of the protein-encoding genome, we ran pathways analysis on the genes that encode all aptamers used in the analysis (*n* = 7214) to establish a baseline of associated pathways. The top pathways are associated with the immune system and cytokine signaling (Supplementary Method Fig. 4), as expected from a platform developed for biological research. Top pathways are displayed in the main text as horizontal bar plots, ordered by decreasing (−log_10_) adjusted *p*-values (*x*-axis) with the pathway term displayed on the *y*-axis. The bars are colored according to their pathway library. Pathways analysis results tables generated in this study are supplied in the **Source Data** accompanying this publication.

### Surrogate variable analysis

Generation of high-dimensional omics data unavoidably captures variation (noise) from unwanted sources. Cord blood samples are particularly sensitive to this consideration as the unique biological, environmental, and physiological factors associated with pregnancy/birth as well as the logistics of on-site, post-delivery collection introduces sources of potential variation that are often difficult to record, such as the precise length of labor, time between delivery and cord blood clamping for collection, and time between clamping and sample processing. For this reason, we employ Surrogate Variable Analysis (SVA)^17^ to identify latent sources of unwanted variation present in the data that is unrelated to variables of interest (e.g., SGA status). The RFU values were log-transformed for input and the number of surrogate variables was estimated with the *num.sv* function using the “be” procedure with 200 permutations. The *sva* function was run with for the estimated number surrogate variables with 200 iterations. The surrogate variables were used as input into Limma models to account for unwanted variation during differential expression analysis and used as covariates in the *removeBatchEffect* function to create an SVA-corrected data matrix for feature selection and consensus clustering (see below). In general, the SVs strongly correlated with the top PC, which were those that capture the strongest technical variation in the data (cohort of origin), and presumably additional unwanted technical/biological variation. Additionally, PCA analysis demonstrated that adjusting for SVs effectively corrects/removes major sources of unwanted variation. Supplementary method Fig. 5 shows plots generated during SVA.

### Sparse partial least squares–discriminatory analysis (sPLS-DA)

To assess whether the cord blood-derived proteomic profiles could be stratified based on SGA status with a multivariate approach, we employed sPLS-DA with the mixOmics R package^51^. The tune.splsda() function was used to determine the (sparse) number of features for two components from the range 5, 6, 7, 8, 9, 10, 15, 20, 25, 30, 50. The perf() function was used to assess the model accuracy with the “Mfold” approach employing fivefold repeated 200 times.

### Feature selection

We used PLS classification to identify aptamers consistently associated with SGA status in cohorts contributing cord blood samples. PLS was run on the SVA-corrected log(RFU) data from each cohort separately with the *opls* function from the rpols R package^52^, with 5× cross-validation permutated 200 times. Aptamers were considered important in the stratification of SGA status for a given cohort if the Variable Importance in Projection (VIP) value was above 1.5. We assessed alternative VIP thresholds (1.0, 1.2, 1.3, 1.35, 1.4, 1.45, 1.55, 1.6, 1.7) and found that the outcome was largely consistent with different VIP values (see below in “consensus clustering” section). The VIP metric estimates the importance of each variable (aptamer) in the projection generated from a PLS model. In general, variables with VIP scores greater than 1 are considered important for given model/projection. Using this approach, 837 aptamers were selected from samples from the CCCEH cohort, 890 from COAST, 698 from IIS, 695 from TCRS, and 731 from WISC. Aptamers were retained if they were selected in at least 3/5 (60%, i.e., a majority) of the cohorts. From this, there were in 102 aptamers considered consistently associated with SGA status available for consensus clustering analysis. Output metrics and associated plots of the PLS feature selection are shown in Supplementary method Fig. 6.

### Consensus clustering

We used the ConsensusClusterPlus R package^53^ to apply hierarchical consensus clustering to the SVA-corrected data, restricted to the aptamers identified as consistently associated with SGA during feature selection. The consensus cluster approach randomly resamples the data and aggregates the results to quantify the occurrence/likelihood of samples being assigned the same cluster, resulting in robust and stable clustering. The hclust algorithm was used with Ward’s D2 linkage and 1-Pearson correlation distance, and clustering was resampled 1000 times with all aptamers and 80% of the samples randomly selected for each resample. We tested clustering up to 25 clusters and found that 7 was the optimum number of clusters for this dataset from the Cumulative Density Function and consensus matrix. Output plots from ConsensusClusterPlus optimization are shown in Supplementary Method Fig. 7. Additionally, we assessed different VIP thresholds (1.0, 1.2, 1.3, 1.35, 1.4, 1.45, 1.55, 1.6, 1.7) to assess whether the consensus clustering results were robust to alternative VIP values. Overall, the results showed that the consensus clustering outcomes using alternative VIP values were consistent with the chosen VIP value of 1.5 (Supplementary Method Fig. 8). Whilst a VIP of 1.0 included too many aptamers (*n* = 1962) and VIP of 1.7 included too few (*n* = 20), the remaining VIP values exhibited a distinct cluster (Cluster A) enriched with approximately 75% SGA subjects (range 64.86–85.19%), aligning with the consensus clustering outcomes with a VIP threshold of 1.5.

### Sensitivity analysis

To confirm that the findings from the consensus cluster analysis were not driven by a small number of outlying samples, we performed a sensitivity analysis by reducing the number of input samples to more homogeneous subsets according to decreasing confidence regions of the first two principal components. PCA was performed on the log-transformed SVA-adjusted RFU values and confidence regions of 95, 90, 85, 80, and 75% were assessed (Supplementary Method Fig. 9). Confidence regions were defined using the percentile (e.g., 95th) of the Chi-square distribution with two degrees of freedom. The ellipse radius was set to the square root of the Chi-squared percentile so the resulting ellipse enclosed the central probability mass of that percentile (e.g., 95%) for the approximately bivariate (PC1 & 2) normal distribution, retaining samples closest to the multivariate mean. Consensus clustering on samples retained from each confidence region identified a cluster (Cluster A) which was enriched for SGA samples and exhibited axon guidance-related pathways from genes encoding dysregulated proteins between these SGA samples and all AGA samples, aligning with the findings using the full dataset.

### Module detection

We employed Fuzzy clustering by Local Approximation of MEmbership (FLAME)^18^ to identify protein co-regulation modules in the data. The FLAME algorithm constructs a neighborhood graph and assigns objects (aptamers) into clusters with respect to Cluster Specific Objects (CSO), the aptamers with archetypal features that typify subregions in the graph. An iterative converging process is applied outwards from CSOs to assign each object a fuzzy membership approximated from the memberships of its neighboring aptamers. The RFU data was log-transformed and adjusted for cohort with the *removeBatchEffect* function from the limma package. Aptamers with expression of one standard deviation below the average of the median aptamer values were excluded, and the remaining 6,199 aptamers were rescaled between −1 and 1 for analysis. FLAME was run in Gene Expression Data Analysis Studio and the modules were optimized for distance (Cosine, Pearson, Euclidian), threshold (0, 5, 10, 20%), Partition Index, and K-nearest neighbor (kNN) number (Supplementary Method Fig. 10). Following optimization, cosine distance with a 0% threshold and 7 kNNs were selected, and these parameters were also used for the analysis of individual cohorts. Module assignment of each aptamer is supplied in the **Source Data**. Network wiring diagrams were constructed on the cosine measure matrix of the same data used for the FLAME analysis (excluding rescaling between −1 and 1), and edges with values < 0.6 and nodes with degree <10 were excluded to aid visualization. Networks were graphed with the igraph package using the Fruchterman-Reingold layout.

### Protein-protein interaction (PPI) networks

We used STRING (Version 12.0)^19^ to construct PPI networks to further characterize modules of interest discovered by FLAME. The STRING database (available at https://string-db.org/) consists of known and predicted protein-protein interactions and include physical (direct) and functional (indirect) associations. Networks were restricted to interaction evidence from experimental and database sources with a minimum interaction score strength requirement of 0.4 (medium), with unconnected nodes not visualized. A screenshot of the PPI network input setting parameters is supplied in Supplementary Method Fig. 11.

### Statistical analysis

The following functions were used for standard statistical tests (from the stats R package unless otherwise stated). Two-sided Fisher’s test with *fisher.test* function. Kruskal–Wallis rank sum test with the *kruskal.test* function with Dunn’s post hoc test of multiple comparisons with the *dunnTest* function (FSA package; a wrapper of the *dunn.test* function (dunn.test package)). Two-sided Mann–Whitney *U* test with the *wilcox.test* function. Spearman’s rank correlation with the *cor* function. Linear modelling with the *lm* function. For the linear model of later life protein expression and lung function measurements, power estimate based on simulated protein expression indicated that the sample size was powered to detect moderate-to-large effect size, but may be limited in detecting small effect sizes. *P*-value adjustment for multiple comparisons with the *p.adjust* function using the BH method. The GLI reference equations^20,21^ were used to calculate FEV_1_, FVC, and FEV_1_/FVC z-scores, which adjust for age, sex, and height. The GLI reference population includes >97,000 records, collected from 72 centers in 33 countries, from healthy non-smokers aged between 2.5 and 95 years (55.3% females)^20^. Height, Sex, Age, FEV_1_, and FVC were supplied to the online resource (available at https://gli-calculator.ersnet.org/index.html) to calculates z-scores, and the analysis was run as “race-neutral”. For age, the TCRS cohort samples were set at 36 (36 year follow up), the IIS cohort was set to their age at spirometry testing (~ 8–10 yrs), and the WHEALS cohort was set to 12 (midpoint in the 11–13 yr spirometry testing age range).

### Elastic net regression models

Elastic net models were trained to predict FEV_1_, FVC, and the FEV_1_ percentage of FVC from childhood/adult protein profiles, with cohort (thus age), sex, and height included as covariates. Different models were trained separately with all available aptamers (*n* = 7154) and with only the childhood/adult corresponding aptamers that were identified from the AG-SGA versus AGA analysis of cord blood-derived profiles (*n* = 221, as shown in the volcano plot in Fig. 1B(i)), for each lung function measurement. For each model, the data was split into the same training set (~ 70%, *n* = 88), and test/validation set (~ 30%, *n* = 38) that was not used in training (held-out). The *α* value was optimized (with the cv.glmnet function from the glmnet R package) as that with the lowest cross-validated mean squared error (MSE) from the range 0-to-1 increasing by 0.1 increments. The α for each model is shown in Supplementary Method Table 2. Final models were trained with cv.glmnet function using the optimized *α* and tenfold cross validation. Two lambda (*λ*) values were assessed; the *λ* with the smallest cross-validation MSE (“minimum”) and the largest *λ* with a cross-validated error within one standard error of the minimum (“1se”). Plots of the models’ prediction on training and test sets, residual analysis, and *R*^2^ values across cross validation folds are shown below in Supplementary Method Fig. 12.

### Genome-wide association study meta-analysis

We leveraged the NHGRI-EBI Catalog of human GWAS^22^ (available at https://www.ebi.ac.uk/gwas/) to identify gene variants linked to pulmonary traits from previously published GWAS studies. We used the trait labels “forced expiratory volume” (with synonyms “FEV1”, “FEVt”, and “timed vital capacity”) and “vital capacity” (with synonyms “FVC” and “forced vital capacity”) to query FEV_1_ and FVC, respectively. Outputs from the GWAS queries are supplied in the **Source Data**. Variants without an annotated chromosome location or from sex chromosomes were excluded. Significant variants associated with our search terms were visualized with bar plots ordered by chromosome and chromosomal location. Axon guidance-related genes (*n* = 707) in this context were considered those included in the “Axon guidance” pathways from KEGG (hsa04360), GO:Biological Process (GO:0007411) or Reactome (R-HSA-422475).

### Sheep (Ovis aries) model

Studies on pregnant Columbia-Rambouillet ewes were approved by the University of Arizona Institutional Animal Care and Use Committee and performed at the Agricultural Research Complex, which is accredited by the American Association for Accreditation of Laboratory Animal Care International. Columbia-Rambouillet crossbred ewes with singleton pregnancies were purchased from the University of Arizona Sheep Unit and transported to the laboratory at 35 ± 2 days of gestation age (dGA). The ewes were two to four years of age with unknown parity. Singleton fetuses were determined by ultrasonography prior to group assignment. Ewes were assigned by a simple randomization method into one of two groups, control and FGR. Placental insufficiency-induced FGR fetuses (*n* = 2; one female, one male) were created by exposing pregnant ewes to elevated ambient temperatures (40 °C for 12 h; 35 °C for 12 h; dew point 22 °C) from 40 ± 1 to 91 ± 1 dGA (a range covering approximately from 30 to 65% of the gestational timeline) (term 149 dGA) as described previously^54^. Control fetuses (*n* = 2; one female, one male) were from ewes maintained at 22 ± 1 °C that were fed Standard Bread Alfalfa Pellets (Sacate Pellet Mills, Inc.) supplemented with trace minerals for sheep (Code #1131-Z, Maid Rite Feeds, Willcox AZ) to the average *ad libitum* feed intake of ewes in the FGR group. The alfalfa diet was fed to meet 100% of the Nutrient Requirements of Small Ruminants as reported by the National Research Council^55^, and consisted of the following; Crude Protein (not less than 14%), Crude fat (not less than 1.5%), Crude fiber (not more than 28%), ash (not more than 12%), added minerals (not more than 0.1%). Water and salt were available to ewes *ad libitum*. After the hyperthermic exposure, all ewes were maintained in a thermoneutral environment alongside ewes in the control group. Ewes were monitored daily throughout the treatment and each animal was fed and watered daily with health status was recorded. At the end of the treatment the ewe and fetus were humanely killed with an over-dose of pentobarbital sodium given intravenously through the maternal and fetal femoral vein (86 mg/kg, Euthasol; VirbacAnimal Health, Fort Worth, TX). Death was confirmed by loss of heartbeat and breath and with a bilateral pneumothorax as a secondary method. Fetal samples were collected from the brain, heart and lung between 130 and 135 dGA. Sheep were used as they are a standard model animal for study of fetal physiology, and physiological characteristics of the heat stress FGR fetal sheep model parallel those reported for human IUGR fetuses and other sheep models of placental restriction^56,57^, which indicates these characteristics are common for FGR/placental insufficiency and not imposed by heat stress directly. The reporting of this model abides by the ARRIVE guidelines for reporting of in vivo work.

### Single cell RNA-sequencing (scRNA-Seq)

#### Sample processing

For each tissue dissociation experiment, three different fetal sheep tissues (heart, lung, brain) were received in duplicate in PBS and kept on ice until dissociation was performed that same day. All samples were processed within 2.5 h of receiving tissue. Each tissue was taken through dissociation following these steps: tissues were removed from tubes, gently blotted to remove excess moisture, placed on a tared petri dish, weighed, and cut down to size with a scalpel to obtain a specimen no greater than 200 mg/10 ml of dissociation solution. Tissue pieces were gently minced with surgical scissors into ~1 mm^2^ pieces with ~1 mL Leibovitz L-15 (Gibco, 11-415-064) to avoid excess drying of tissue. For each sample, minced tissues were transferred to an autoclaved scintillation vial with a micro stir bar and 10 mL Dissociation Media (Leibovitz L-15 [Gibco, 11-415-064], 15 μM HEPES [Corning, 25-060-CI], 6 g/L glucose [Sigma Aldrich, G7021-100G], 10% FBS [Omega-Scientific, FB-11], 500U/ml Collagenase IV [Worthington Biochemical Corporation, LS004188] and 0.1 mg/ml DNase I [Sigma Aldrich, DN25]). Tissues in media were incubated for 30 min at 37 °C at 80 rpm rotation. After 30 min incubation, 5 mL of FBS was added to each vial to neutralize enzyme activity and samples were passed through a 100μm filter into a 50 mL conical tube. Scintillation vials were washed twice with 5 mL of rinsing media consisting of PBS with 5 mM EDTA (Fisher Scientific, BP2482-100) and 5% FBS. These volumes were used to gently wash cells through the filter. Samples were centrifuged at 300 × *g* for 5 min at 4 °C and the pellet was resuspended in 2 mL rinsing media. The 2 mL sample resuspensions were filtered again through a 70 μm filter and counted in triplicate using trypan blue on a Countess II (Invitrogen, AMQAX1000). Average cell counts were calculated. Samples were centrifuged again at 300 × *g* for 5 min at 4 °C and the cells were resuspended in 5% FBS in PBS at a concentration of 1000 total cells/μL (1 million cells/mL). Once resuspended at the appropriate concentration, samples were handed off to Arizona Genetics Core for downstream single-cell sequencing.

#### Data generation

Single-cell suspensions were submitted for single-cell RNA-sequencing (scRNA-seq) at the Arizona Genetics Core using the 10× Genomics Universal 3′ Gene Expression workflow. Single-cell emulsions were generated, and barcoded mRNA from individual cells was captured and reverse-transcribed into cDNA using the 10× Genomics Chromium X Controller platform. The resulting cDNA was used to generate scRNA-seq libraries, which were then sequenced according to the recommended guidelines on either an Illumina NovaSeq 6000 or Element Biosciences AVITI sequencing instrument. Each sample was run in duplicate to increase read depth and cell numbers.

#### Data preprocessing

Raw.fastq files generated for each sample (in duplicate) were processed with CellRanger count (version 9.0.0) via the 10× cloud platform using the Oar_rambouillet_v1.0 reference genome, and output FastQC reports were assessed for post-alignment QC. Raw feature by barcode matrix.h5 files were imported into R (version 4.3.2) for QC and analysis.

#### Sample quality control and integration

Barcodes were ranked by total Unique Molecular Identifier count detected with the *barcodeRanks* function from the DropletUtils package using default parameters, and knee and inflection points of the resulting curve were calculated. The *emptyDrops* function (DropletUtils) was used to differentiate barcodes associated with single cells and exclude those containing only ambient RNA using the inflection point using 10,000 iterations. Genes were removed if not expressed by any cell, and then filter to only those expressed in at least 1% of the total cells. QC metrics, including mitochondrial and ribosomal content, were calculated with the addPerCellQC function from the scuttle package. Histograms were generated to visualize the number of genes and transcripts expressed per cell. Cells were excluded if they had less than 1000 transcripts for all samples, and/or less than 500 genes for 9 samples, less than 250 genes for 7 samples, and less than 50 genes for 3 samples. The difference in the gene expression threshold corresponds to differing homogeneity of the single-tissue single cell preparation. Mitochondrial gene expression was not detected or very small in this data set.

A Seurat (version 5.0.3)^58,59^ object was created for each sample with the *CreateSeuratObject* function, and a standard Seurat pipeline was applied using the *NormalizeData*, *FindVariableFeatures*, *ScaleData*, and *RunPCA* functions. PCA loadings and components were assessed with the *VizDimLoadings* and *DimPlot*, respectively. The *FindNeighbors* (dims = 1:15) and FindClusters (resolution = 0.8) functions were used for cell clustering, and the RunUMAP (dims = 1:15) function was used for dimensionality reduction to assess clustering. Heatmaps were generated with the *DoHeatmap* function to assess top marker gene expression. A consensus approach was used to remove Doublets if they were identified by either DoubletFinder^60^, scDblFinder^61^, or scds^62^. Duplicate samples were merged and normalized with *SCTransform*^63^ (vst.flavor = “v2”) using 3000 variable features and integrated with the *IntegrateLayers* function. The canonical correlation analysis, reciprocal PCA, and Harmony^64^ methods were assessed for integration effectiveness, and the Harmony approach was selected. Samples were integrated separately by organ and cell types were annotated using established marker genes. For the purposed of this study, it was not necessary to deeply characterized each cell type, so high-level annotations were used with similar cell subtype differentiated as numbered subclusters (e.g., Endothelial_1 and Endothelial_2), although more granular annotations are shown in Supplementary Fig. 13, for example Lung epithelial 1 and 4 subclusters likely correspond to AT II and AT I epithelial cells, respectively. Clusters containing only a small number of cells were excluded. Following preprocessing, QC, and integration, there were 47,420 cells available for analysis. Key metrics for each sample are supplied in Supplementary Method Table 3.

#### scRNA-seq analysis

A representative UMAP plot for each organ was included to show all cell types. For this, the samples were normalized with the *SCTransform* function (vst.flavor = “v2”) using 3000 variable features, PCA was applied with the *RunPCA* function, and the samples were integrated with the *IntegrateLayers* function (method = “HarmonyIntegration”, orig.reduction = “pca”, assay = “SCT”, normalization.method = “SCT”). The *FindNeighbors* (dims = 1:30) and *FindClusters* (resolution = 0.2 for brain, 0.1 for heart, and 0.08 for lung samples) were used to identify clusters, the *RunUMAP* function was used to construct the UMAP, and *DimPlot* was used for plotting with cells labelled according to their cell type. To focus the analysis on axon guidance genes, we took the intersect of genes from the “Axon guidance” pathways from KEGG (hsa04360), GO:Biological Process (GO:0007411) or Reactome (R-HSA-422475), resulting in 707 genes (the same input genes used for the GWAS meta-analysis). Of these, 563 were present among the genes available following pre-processing and QC in the scRNA-Seq data set. Heatmap was created with the *DoHeatmap* function using the top 5 genes identified for each cluster using the *FindAllMarkers* function. MAST adjusting for random effects (MAST + RE)^23,65,66^ was used for differential expression analysis between FGR positive and negative samples. Cell types were excluded from the analysis if they had less than 50 cells per condition and/or less than 5 cells per sheep. Log_2_-transformed raw counts were used as input and MAST was applied adjusting for cellular detection rate (CDR) and sheep as a random variable ( ~ Condition + CDR + (1 | Sheep)). Due to the small number of donors, meaningful analysis of sex differences was not possible. However, there was equal representation of sex from the fetuses of each group. The results were visualized as a combined volcano plot created with R’s base plotting function. Pathways analysis was run on the gene names of differentially expressed genes between FGR and control for each cell type using the same process described above in the *Pathways* analysis section.

### Reporting summary

Further information on research design is available in the Nature Portfolio Reporting Summary linked to this article.

## Supplementary information

Supplementary Information

Reporting Summary

Transparent Peer Review file

## Source data

Source Data

## Data Availability

Raw and processed proteomics data have been deposited to the PRIDE^67^ Affinity Proteomics Archive repository with the dataset identifier PAD000036. The raw scRNA-Seq data generated from the sheep model is available via the NCBI Sequence Read Archive under BioProject accession PRJNA1444340 and processed count data is available on Dryad under: 10.5061/dryad.5mkkwh7h3 (10.5061/dryad.5mkkwh7h3). There are no restrictions on data access. Source data used for graphing is provided with this paper. The data generated from differential expression, pathways, linear modeling, and module detection and enrichment analyses in this study are provided in the Source Data file. This study used data from the NHGRI-EBI Catalog of human Genome-Wide Association Studies^22^ (available at https://www.ebi.ac.uk/gwas/); summary statistics were downloaded on 01/06/2025. This study used the Global Lung Function Initiative^20,21^ reference population via the online resource (available at https://gli-calculator.ersnet.org/index.html). Source data are provided with this paper.

## Code availability

Code used for analysis in this manuscript is available via Zenodo under: 10.5281/zenodo.19226999 (10.5281/zenodo.19226999)^68^.
