# Supplementary material for: Multicohort analysis unveils axon guidance pathways linking small for gestational age to spirometric restriction
Source: Nat Commun. 2026 May 2;17:5952. doi: 10.1038/s41467-026-72490-w (PMC13342533; doi:10.1038/s41467-026-72490-w)
Supplement: Supplementary file 1 — Supplementary Information [file 41467_2026_72490_MOESM1_ESM.pdf]

Supplementary Material

Multicohort analysis unveils axon guidance pathways linking small for gestational age to spirometric restriction

James F. Read[1,*], Debra A. Stern[1], Tara F. Carr[1], Amber L. Spangenberg[1], Meiven Yang[2], Rosa I Luna-Ramirez[3], Stefano Guerra[1], Wayne J. Morgan[1], Alex T. Binder[4], Jeffrey J. VanWormer[5], Christine M. Seroogy[6], Rachel L. Miller[7], Edward M. Zoratti[8], Carole Ober[9], Daniel J. Jackson[6], Sean W. Limesand[3], Diane R. Gold[10], James E. Gern[6], CADRE consortium†, Anthony Bosco[1,2,#], Fernando D. Martinez[1,#].

[1]Asthma and Airway Disease Research Center, University of Arizona, Tucson, AZ, USA.
[2]Department of Immunobiology, The University of Arizona College of Medicine, Tucson, AZ, USA.
[3]School of Animal and Comparative Biomedical Sciences, University of Arizona, Tucson, AZ, USA.
[4]Office of Informatics and Information Technology, University of Wisconsin School of Medicine and Public Health, Madison, WI, USA.
[5]Center for Clinical Epidemiology & Population Health, Marshfield Clinic Research Institute, Marshfield, WI, USA.
[6]Department of Pediatrics, University of Wisconsin School of Medicine and Public Health, Madison, WI, USA.
[7]Division of Clinical Immunology, Icahn School of Medicine at Mount Sinai, New York, NY, USA
[8]Division of Allergy and Clinical Immunology, Department of Internal Medicine, Henry Ford Health, Detroit, MI, USA
[9]Department of Human Genetics, University of Chicago, Chicago, IL, USA.
[10]Department of Environmental Health, Harvard T. H. Chan School of Public Health, Boston, MA, USA; Channing Division of Network Medicine, Brigham and Women's Hospital and Harvard Medical School, Boston, MA, USA.
* Corresponding author (jread@arizona.edu).
# These Authors contributed equally.
† A list of authors and their affiliations appears at the end of the main paper.

Supplemental Tables and Figures

Supplementary table 1. Overview of geographical locations and race/ethnicity of CADRE consortium cohorts used in the study. This table is an abridged version of Table 1 in(1)

Cohort	Initial enrollment	Location	Population	Recruitment years	Cauc/WH	AA	Hispanic	Asian	Other
**CCCEH**	727	Manhattan/ Bronx, NY	General	1998-2006	0	291 (40%)	436 (60%)	0	0
**COAST**	289	Suburban Madison, WI	High risk, suburban	1998-2000	243 (87%)	12 (4%)	8 (3%)	0 (0%)	7 (2%)
**IIS**	482	Tucson, AZ	General	1997-2003	280 (58%)	24 (5%)	125 (26%)	0 (0%)	53 (11%)
**TCRS**	1246	Tucson, AZ	General	1980-1984	822 (66%)	50 (4%)	312 (25%)	0 (0%)	62 (5%)
**WHEALS**	1258	Metropolitan Detroit, MI	General	2003-2007	25 (26%)	787 (63%)	81 (6%)	30 (2%)	35 (3%)
**WISC**	212	Rural Wisconsin	Rural, farm/ non-farm	2013-2019	204 (96%)	4 (2%)	2 (1%)	2 (1%)	0 (0%)

*Abbreviations: CCCEH, Columbia Center for Children's Environmental Health Cohort; COAST, Childhood Origins of Asthma study; IIS, Infant Immune Study; TCRS, Tucson Children's Respiratory Study; WHEALS, Wayne County Health Environment Allergy and Asthma Longitudinal Study; WISC, Wisconsin Infant Study Cohort; Cauc/WH, Caucasian/White; AA, African American.

Supplementary table 2. Relevant birth metrics stratified by cohort of origin.

	CCCEH (n = 43)	COAST (n = 20)	IIS (n = 40)	TCRS (n = 78)	WISC (n = 26)
**Sex (assigned female at birth) (%)**	22/43 (51.16%)	12/20 (60%)	20/40 (50%)	40/78 (51.28%)	12/26 (46.15%)
**SGA status (born SGA) (%)**	22/43 (51.16%)	11/20 (55%)	20/40 (50%)	40/78 (51.28%)	15/26 (57.69%)
**Birth weight (grams)*** **mean (95% CI); [min - max]**	3,111.86 (2,969.46 - 3,254.26) [2,130 - 3,995]	2,980.00 (2,759.22 - 3,200.78) [2,240 - 3,970]	3,079.18 (2,913.65 - 3,244.72) [2,041 - 4,026]	3,071.90 (2,957.14 - 3,186.65) [2,020 - 4,146]	3,031.0 (2,810.42 - 3,251.58) [1,929 - 3,941]
**birth length (cm)†** **mean (95% CI); [min - max]**	50.12 (49.36 - 50.88) [46.0 - 55.0]	48.49 (47.15 - 49.83) [43.18 - 55.37]	48.97 (48.19 - 49.76) [45.0 - 55.0]	49.29 (48.73 - 49.85) [44.5 - 54.5]	49.26 (48.23 - 50.29) [43.18 - 53.98]
**Head circumference at birth (cm)‡** **mean (95% CI); [min - max]**	33.43 (32.89 - 33.96) [31.0 - 37.0]	33.38 (32.84 - 33.91) [31.0 - 36.5]	33.65 (33.14 - 34.17) [30.0 - 38.0]	33.98 (33.61 - 34.35) [30.5 - 37.5]	Not recorded
**Gestational age (weeks)** **mean (95% CI); [min - max]**	39.16 (38.74 - 39.58) [36.0 - 42.0]	39.05 (38.36 - 39.74) [36.0 - 41.0]	39.65 (39.33- 39.97) [38.0 - 42.0]	39.38 (39.09 - 39.67) [35.0 - 42.0]	38.88 (38.44 - 39.33) [36.0 - 41.0]

*Abbreviations: CCCEH, Columbia Center for Children's Environmental Health Cohort; COAST, Childhood Origins of Asthma study; IIS, Infant Immune Study; TCRS, Tucson Children's Respiratory Study; WHEALS, Wayne County Health Environment Allergy and Asthma Longitudinal Study; WISC, Wisconsin Infant Study Cohort; SGA, Small for Gestational Age; CI, Confidence Interval; min, minimum; max, maximum; cm, centimeter.

Supplementary table 3. Number and proportion of AG-SGA versus nonAG-SGA by cohort.

	CCCEH	COAST	IIS	TCRS	WISC
**AG-SGA**	7	3	8	14	4
**nonAG-SGA**	14	6	12	24	7
**Total SGA**	21	9	20	38	11
**% AG-SGA**	33.30%	33.30%	40.00%	36.80%	36.40%

*Abbreviations: CCCEH, Columbia Center for Children's Environmental Health Cohort; COAST, Childhood Origins of Asthma study; IIS, Infant Immune Study; TCRS, Tucson Children's Respiratory Study; WHEALS, Wayne County Health Environment Allergy and Asthma Longitudinal Study; WISC, Wisconsin Infant Study Cohort; SGA, Small for Gestational Age.

Supplementary table 4. Relevant variables for individuals providing later life peripheral blood samples, stratified by cohort.

	IIS (n = 19)	TCRS (n = 75)	WHEALS (n = 32)	All (n = 126)
Age	8-13yrs	36yrs	11-13yrs	8-36yrs
Sex (female)	10/19 (52.63%)	40/75 (53.33%)	16/32 (50%)	66/126 (52.38%)
SGA status at birth	8/19 (42.1%)	22/75 (29.33%)	16/32 (50%)	46/126 (36.51%)
FEV_1 (liters) mean [range]	1.94 [1.52-2.91]	3.68 [2.2 - 5.76]	2.45 [1.33 - 4.07]	3.13 [1.33 - 5.76]
FVC (liters) mean [range]	2.25 [1.7 - 3.2]	4.83 [2.92 - 8.11]	3.09 [1.94 - 4.73]	4.0 [1.7 - 8.11]
FEV_1/FVC (%) mean [range]	86.34 [79.19 - 98.45]	76.35 [55.76 - 92.15]	82 [58.52 - 92.83]	79.29 [55.76 - 98.45]
Height (cm) mean [range]	137.7 [126 - 157]	172.6 [154.9 - 200.7]	159.4 [140.9 - 180.0]	163.9 [126 - 200.7]
Weight (kilograms) mean [range]	33 [23 - 58]	86.94 [51.71 - 188.24]	59.35 [34.2 - 104.1]	71.8 [23 - 188.24]
BMI mean [range]	17.26 [12.16 - 23.53]	29.46 [17.81 - 62.35]	22.99 [16.26 - 35.15]	25.97 [12.16 - 62.35]

*Abbreviations: IIS, Infant Immune Study; TCRS, Tucson Children's Respiratory Study; WHEALS, Wayne County Health Environment Allergy and Asthma Longitudinal Study; SGA, Small for Gestational Age; FEV_1, Forced Expiratory Volume in one second; FVC, Forced Vital Capacity; BMI, body mass index.

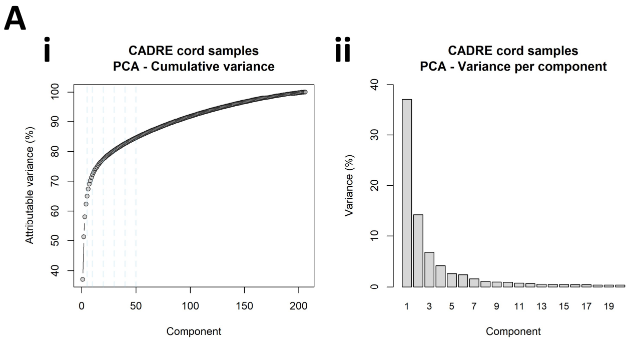

iii

	Variance (%)	Cumulative variance (%)
PC1	36.995	36.995
PC2	14.268	51.263
PC3	6.825	58.088
PC4	4.186	62.274
PC5	2.64	64.914
PC6	2.427	67.341
PC7	1.63	68.971
PC8	1.138	70.109
PC9	1.004	71.113
PC10	0.953	72.066

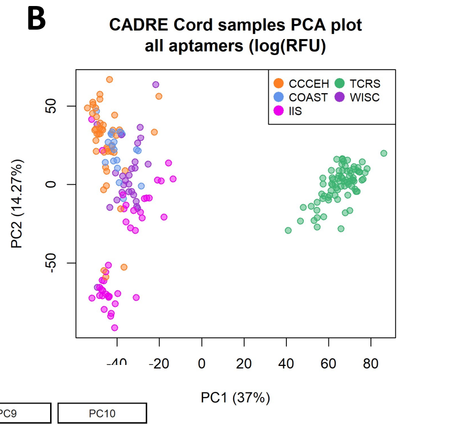

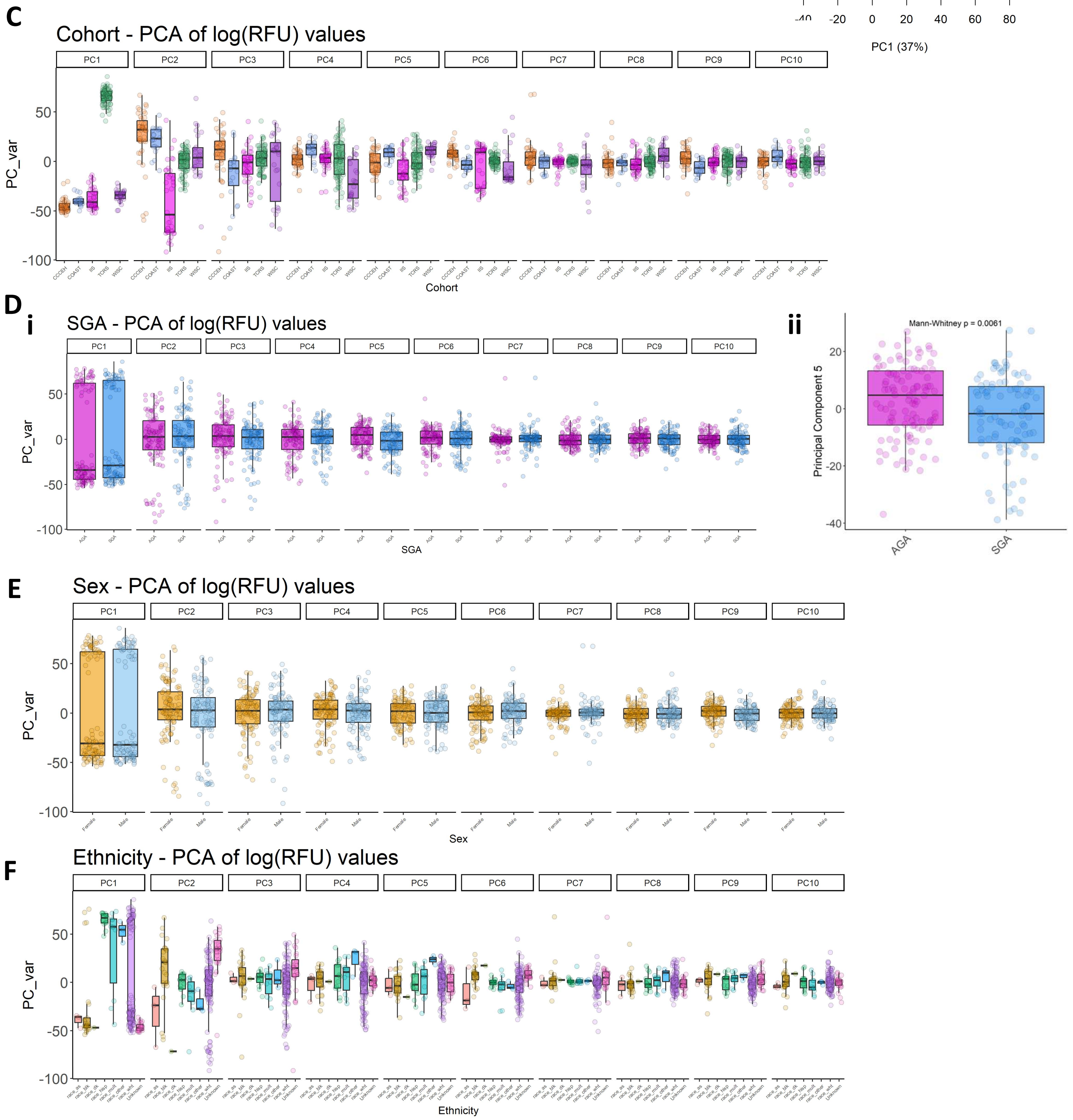

Supplementary figure 1. **A**) Principal component analysis of cord blood-derived proteomic profiles. Contribution of variation displayed as cumulative dot plot (**i**), per component bar plot (**ii**), and table (**iii**). **B**) Scatter plot of the first two PCs colored by the cohort of origin of the cord blood samples (n=207). **C**) Boxplots showing the top 10 PCs stratified by cohort of origin (n=207 per PC). **D**) Boxplots showing the top 10 PCs stratified by SGA status at birth (n=207 per PC) (**i**) and dedicated boxplot of SGA-associated PC5 showing the (nominal) Mann-Whitney p value for the comparison (n=108 AGA and n=99 SGA). **E**) Boxplots showing the top 10 PCs stratified by Sex assigned at birth (n=207 per PC). **F**) Boxplots showing the top 10 PCs stratified by reported ethnicity. Ethnicity was recorded differently between the cohorts and is partially confounded with cohort.

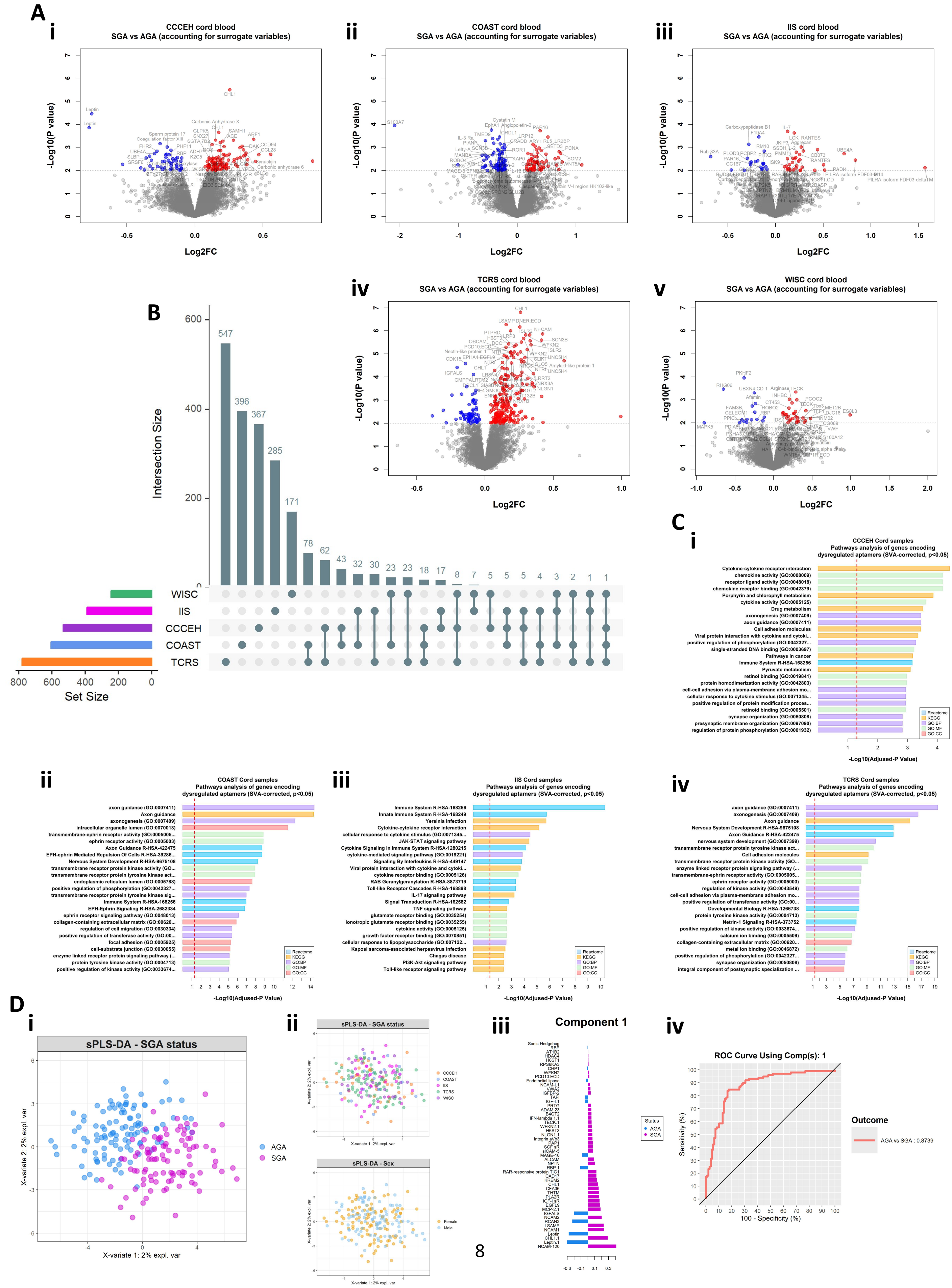
A
i
CCCEH cord blood
SGA vs AGA (accounting for surrogate variables)
ii
COAST cord blood
SGA vs AGA (accounting for surrogate variables)
iii
IIS cord blood
SGA vs AGA (accounting for surrogate variables)
iv
TCRS cord blood
SGA vs AGA (accounting for surrogate variables)
v
WISC cord blood
SGA vs AGA (accounting for surrogate variables)
-Log10(P value)
Log2FC
CHL1
Leptin
B
Intersection Size
547
396
367
285
171
78
62
43
32
30
23
23
18
17
8
7
5
5
5
4
3
2
1
1
WISC
IIS
CCCEH
COAST
TCRS
Set Size
C
i
CCCEH Cord samples
Pathways analysis of genes encoding dysregulated aptamers (SVA-corrected, p<0.05)
Cytokine-cytokine receptor interaction
chemokine activity (GO:0008009)
receptor ligand activity (GO:0048018)
chemokine receptor binding (GO:0042379)
Porphyrin and chlorophyll metabolism
cytokine activity (GO:0005125)
Drug metabolism
axonogenesis (GO:0007409)
axon guidance (GO:0007411)
Cell adhesion molecules
Viral protein interaction with cytokine and cytoki...
positive regulation of phosphorylation (GO:0042327...
single-stranded DNA binding (GO:0003697)
Pathways in cancer
Immune System R-HSA-168256
Pyruvate metabolism
retinol binding (GO:0019841)
protein homodimerization activity (GO:0042803)
cell-cell adhesion via plasma-membrane adhesion mo...
cellular response to cytokine stimulus (GO:0071345...
positive regulation of protein modification proces...
retinoid binding (GO:0005501)
synapse organization (GO:0050808)
presynaptic membrane organization (GO:0097090)
regulation of protein phosphorylation (GO:0001932)
Reactome
KEGG
GO:BP
GO:MF
GO:CC
-Log10(Adjusted-P Value)
ii
COAST Cord samples
Pathways analysis of genes encoding dysregulated aptamers (SVA-corrected, p<0.05)
axon guidance (GO:0007411)
Axon guidance
axonogenesis (GO:0007409)
intracellular organelle lumen (GO:0070013)
transmembrane-ephrin receptor activity (GO:0005005...
ephrin receptor activity (GO:0005003)
Axon Guidance R-HSA-422475
EPH-ephrin Mediated Repulsion Of Cells R-HSA-39286...
Nervous System Development R-HSA-9675108
transmembrane receptor protein kinase activity (GO...
transmembrane receptor protein tyrosine kinase act...
endoplasmic reticulum lumen (GO:0005788)
positive regulation of phosphorylation (GO:0042327...
transmembrane receptor protein tyrosine kinase sig...
Immune System R-HSA-168256
EPH-Ephrin Signaling R-HSA-2682334
ephrin receptor signaling pathway (GO:0048013)
collagen-containing extracellular matrix (GO:00620...
regulation of cell migration (GO:0030334)
positive regulation of transferase activity (GO:00...
focal adhesion (GO:0005925)
cell-substrate junction (GO:0030055)
enzyme linked receptor protein signaling pathway (...
protein tyrosine kinase activity (GO:0004713)
positive regulation of kinase activity (GO:0033674...
-Log10(Adjuseed-P Value)
iii
IIS Cord samples
Pathways analysis of genes encoding dysregulated aptamers (SVA-corrected, p<0.05)
Immune System R-HSA-168256
Innate Immune System R-HSA-168249
Yersinia infection
Cytokine-cytokine receptor interaction
cellular response to cytokine stimulus (GO:0071345...
JAK-STAT signaling pathway
Cytokine Signaling In Immune System R-HSA-1280215
cytokine-mediated signaling pathway (GO:0019221)
Signaling By Interleukins R-HSA-449147
Viral protein interaction with cytokine and cytoki...
cytokine receptor binding (GO:0005126)
RAB Geranylgeranylation R-HSA-8873719
Toll-like Receptor Cascades R-HSA-168898
IL-17 signaling pathway
Signal Transduction R-HSA-162582
TNF signaling pathway
glutamate receptor binding (GO:0035254)
ionotropic glutamate receptor binding (GO:0035255)
cytokine activity (GO:0005125)
growth factor receptor binding (GO:0070851)
cellular response to lipopolysaccharide (GO:007122...
Kaposi sarcoma-associated herpesvirus infection
Chagas disease
PI3K-Akt signaling pathway
Toll-like receptor signaling pathway
-Log10(Adjusted-P Value)
iv
TCRS Cord samples
Pathways analysis of genes encoding dysregulated aptamers (SVA-corrected, p<0.05)
axon guidance (GO:0007411)
axonogenesis (GO:0007409)
Axon guidance
Nervous System Development R-HSA-9675108
Axon Guidance R-HSA-422475
nervous system development (GO:0007399)
transmembrane receptor protein tyrosine kinase act...
Cell adhesion molecules
transmembrane receptor protein kinase activity (GO...
enzyme linked receptor protein signaling pathway (...
transmembrane-ephrin receptor activity (GO:0005005...
ephrin receptor activity (GO:0005003)
regulation of kinase activity (GO:0043549)
cell-cell adhesion via plasma-membrane adhesion mo...
positive regulation of transferase activity (GO:00...
Developmental Biology R-HSA-1266738
protein tyrosine kinase activity (GO:0004713)
Netrin-1 Signaling R-HSA-373752
positive regulation of kinase activity (GO:0033674...
calcium ion binding (GO:0005509)
collagen-containing extracellular matrix (GO:00620...
metal ion binding (GO:0046872)
positive regulation of phosphorylation (GO:0042327...
synapse organization (GO:0050808)
integral component of postsynaptic specialization ...
-Log10(Adjusted-P Value)
D
i
sPLS-DA - SGA status
X-variate 1: 2% expl. var
X-variate 2: 2% expl. var
AGA
SGA
ii
sPLS-DA - SGA status
CCCEH
COAST
IIS
TCRS
WISC
sPLS-DA - Sex
Female
Male
iii
Component 1
Status
AGA
SGA
iv
ROC Curve Using Comp(s): 1
Sensitivity (%)
100 - Specificity (%)
Outcome
AGA vs SGA : 0.8739

Supplementary figure 2. Differential expression analysis (limma) for the comparison between cord blood-derived proteomic profiles from subjects born SGA versus AGA. (**A**) Volcano plot showing $\log_2$ fold change and ($-\log_{10}$) BH-adjusted p values with selected significantly dysregulated aptamers labeled with their corresponding protein name. (**B**) Upset plot demonstrating the overlap of significantly dysregulated aptamers from the comparison between SGA and AGA status for each cohort (**C**) Horizontal bar plot displays the top 25 enriched pathways of genes encoding significantly dysregulated aptamers (BH-adj p value < 0.05) for each cohort (results for WISC cohort not shown, supplied in the Data supplement). The x-axis shows the adjusted enrichment p value and the y-axis shows the pathway terms. Bars are colored according to the repository of the enriched term. The full lists of DEGs and enriched pathways for each cohort is supplied in the Supplementary Data. **D**) Outputs from sparse Partial Least Squares discriminatory analysis (sPLS-DA) to classify cord blood-derived proteomic profiles based on SGA status. Variate plots of cord blood-derived profiles (n=207) demonstrate stratification by SGA status (**i**), but not Cohort or Sex (**ii**). Top feature loadings for component 1 display aptamers associated with axon guidance and immune processes (**iii**). Several aptamer names include an additional number (e.g., “Leptin.1”), which was included to handle duplicate protein names for aptamers. 5-fold cross validation shows an AUC-ROC of 0.874 (**iv**).

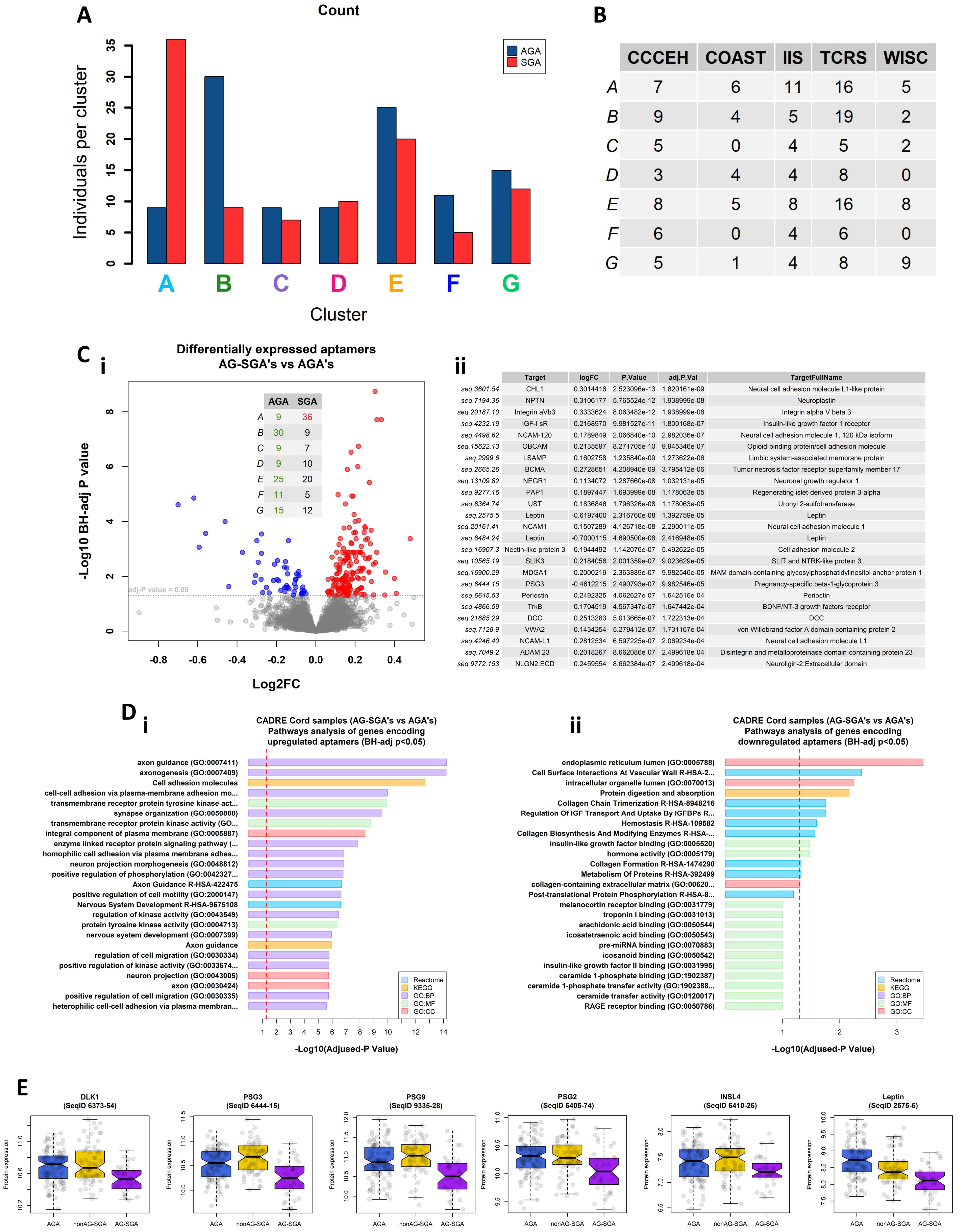

B

	CCCEH	COAST	IIS	TCRS	WISC
A	7	6	11	16	5
B	9	4	5	19	2
C	5	0	4	5	2
D	3	4	4	8	0
E	8	5	8	16	8
F	6	0	4	6	0
G	5	1	4	8	9

C i (inset)

	AGA	SGA
A	9	36
B	30	9
C	9	7
D	9	10
E	25	20
F	11	5
G	15	12

ii

	Target	logFC	P.Value	adj.P.Val	TargetFullName
seq.3601.54	CHL1	0.3014416	2.523096e-13	1.820161e-09	Neural cell adhesion molecule L1-like protein
seq.7194.36	NPTN	0.3106177	5.765524e-12	1.938999e-08	Neuroplastin
seq.20187.10	Integrin aVb3	0.3333624	8.063482e-12	1.938999e-08	Integrin alpha V beta 3
seq.4232.19	IGF-I sR	0.2168970	9.981527e-11	1.800168e-07	Insulin-like growth factor 1 receptor
seq.4498.62	NCAM-120	0.1789849	2.066840e-10	2.982036e-07	Neural cell adhesion molecule 1, 120 kDa isoform
seq.15622.13	OBCAM	0.2135597	8.271705e-10	9.945346e-07	Opioid-binding protein/cell adhesion molecule
seq.2999.6	LSAMP	0.1602758	1.235840e-09	1.273622e-06	Limbic system-associated membrane protein
seq.2665.26	BCMA	0.2728651	4.208940e-09	3.795412e-06	Tumor necrosis factor receptor superfamily member 17
seq.13109.82	NEGR1	0.1134072	1.287660e-08	1.032131e-05	Neuronal growth regulator 1
seq.9277.16	PAP1	0.1897447	1.693999e-08	1.178063e-05	Regenerating islet-derived protein 3-alpha
seq.8364.74	UST	0.1836846	1.796326e-08	1.178063e-05	Uronyl 2-sulfotransferase
seq.2575.5	Leptin	-0.6197400	2.316760e-08	1.392759e-05	Leptin
seq.20161.41	NCAM1	0.1507289	4.126718e-08	2.290011e-05	Neural cell adhesion molecule 1
seq.8484.24	Leptin	-0.7000115	4.690500e-08	2.416948e-05	Leptin
seq.16907.3	Nectin-like protein 3	0.1944492	1.142076e-07	5.492622e-05	Cell adhesion molecule 2
seq.10565.19	SLIK3	0.2184056	2.001359e-07	9.023629e-05	SLIT and NTRK-like protein 3
seq.16900.29	MDGA1	0.2000219	2.363889e-07	9.982546e-05	MAM domain-containing glycosylphosphatidylinositol anchor protein 1
seq.6444.15	PSG3	-0.4612215	2.490793e-07	9.982546e-05	Pregnancy-specific beta-1-glycoprotein 3
seq.6645.53	Periostin	0.2492325	4.062627e-07	1.542515e-04	Periostin
seq.4866.59	TrkB	0.1704519	4.567347e-07	1.647442e-04	BDNF/NT-3 growth factors receptor
seq.21685.29	DCC	0.2513283	5.013665e-07	1.722313e-04	DCC
seq.7128.9	VWA2	0.1434254	5.279412e-07	1.731167e-04	von Willebrand factor A domain-containing protein 2
seq.4246.40	NCAM-L1	0.2812534	6.597225e-07	2.069234e-04	Neural cell adhesion molecule L1
seq.7049.2	ADAM 23	0.2018267	8.662086e-07	2.499618e-04	Disintegrin and metalloproteinase domain-containing protein 23
seq.9772.153	NLGN2:ECD	0.2459554	8.662384e-07	2.499618e-04	Neuroligin-2:Extracellular domain

Supplementary figure 3. **A**) Bar plot showing the number of SGA and AGA subjects per cluster from consensus cluster analysis. **B**) Table displaying the number of subjects from each cohort in the clusters identified by the consensus cluster analysis. **C**) Differential expression analysis (limma) for the comparison between cord blood-derived proteomic profiles from subjects designated AG-SGAs (SGAs in cluster A) versus all AGA. (**i**) Volcano plot showing $\log_2$ fold change and ($-\log_{10}$) BH-adjusted p values with a tabulated overview of the samples used in the comparison (insert, number in red versus number in green). Aptamers considered significantly up- and down-regulated are shown in red and blue, respectively. (**ii**) Tabulated results of the differential expression analysis showing the top 25 dysregulated aptamers. **D**) Horizontal bar plot displays the top 25 enriched pathways of genes encoding significantly dysregulated aptamers (BH-adj p value < 0.05). Pathways analysis was conducted on genes encoding aptamers that were significantly higher (**i**) and lower (**ii**) in SGA individuals, separately. The x-axis shows the adjusted enrichment p value and the y-axis show the pathway terms. Bars are colored according to the repository of the enriched term. **E**) Boxplots of selected pregnancy/placenta associated aptamers downregulated in AG-SGA samples versus AGA. Boxplots show the median and interquartile range (IQR), with whiskers extending to the largest and smallest values within 1.5×IQR. Points represent individual samples (n = 108 AGA, 63 nonAG-SGA, and 36 AG-SGA subjects). The y-axis shows the normalized protein expression. Each aptamer shown recorded an FDR-adjusted p value < 0.05 in the analyses of AG-SGA versus AGA and nonAG-SGA.

A

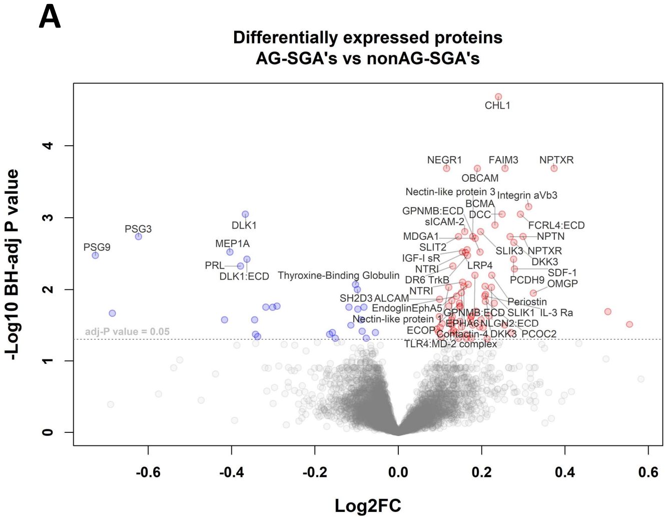

B

	Target	logFC	P.Value	adj.P.Val	TargetFullName
seq.3601.54	CHL1	0.2402184	2.872024e-09	2.071878e-05	Neural cell adhesion molecule L1-like protein
seq.13109.82	NEGR1	0.1158550	6.779435e-08	2.054416e-04	Neuronal growth regulator 1
seq.15511.37	NPTXR	0.3738072	1.004745e-07	2.054416e-04	Neuronal pentraxin receptor
seq.6574.11	FAIM3	0.2558191	1.354411e-07	2.054416e-04	Fas apoptotic inhibitory molecule 3
seq.15622.13	OBCAM	0.1895167	1.423909e-07	2.054416e-04	Opioid-binding protein/cell adhesion molecule
seq.20187.10	Integrin aVb3	0.3127719	5.830872e-07	7.010652e-04	Integrin alpha V beta 3
seq.21685.29	DCC	0.2491312	8.854022e-07	8.927174e-04	DCC
seq.8973.23	FCRL4:ECD	0.2929862	1.107709e-06	8.927174e-04	Fc receptor-like protein 4:Extracellular domain
seq.6373.54	DLK1	-0.3672272	1.113731e-06	8.927174e-04	Protein delta homolog 1
seq.2665.26	BCMA	0.2319842	1.750113e-06	1.262531e-03	Tumor necrosis factor receptor superfamily member 17
seq.14756.29	sICAM-2	0.1593799	2.478749e-06	1.562911e-03	Intercellular adhesion molecule 2
seq.10565.19	SLIK3	0.1974554	2.599796e-06	1.562911e-03	SLIT and NTRK-like protein 3
seq.8997.4	NPTXR	0.2990204	3.275043e-06	1.817397e-03	Neuronal pentraxin receptor
seq.18930.28	SLIT2	0.1443989	3.837146e-06	1.830241e-03	Slit homolog 2 protein
seq.6444.15	PSG3	-0.6234431	3.876344e-06	1.830241e-03	Pregnancy-specific beta-1-glycoprotein 3
seq.16907.3	Nectin-like protein 3	0.1785582	4.161566e-06	1.830241e-03	Cell adhesion molecule 2
seq.7194.36	NPTN	0.2679787	4.313015e-06	1.830241e-03	Neuroplastin
seq.16900.29	MDGA1	0.1843554	4.827148e-06	1.934614e-03	MAM domain-containing glycosylphosphatidylinositol anchor protein 1
seq.10746.24	DKK3	0.2771130	5.803552e-06	2.203517e-03	Dickkopf-related protein 3
seq.4232.19	IGF-I sR	0.1648664	7.699386e-06	2.777169e-03	Insulin-like growth factor 1 receptor
seq.8240.207	GPNMB:ECD	0.1958979	8.784962e-06	2.995581e-03	Transmembrane glycoprotein NMB:Extracellular domain
seq.19558.10	LRP4	0.1538483	9.329275e-06	2.995581e-03	Low-density lipoprotein receptor-related protein 4
seq.19786.26	MEP1A	-0.4043049	9.550646e-06	2.995581e-03	Meprin A subunit alpha
seq.20550.38	NTRI	0.1611747	1.012647e-05	3.043848e-03	Neurotrimin
seq.9335.28	PSG9	-0.7271437	1.197959e-05	3.344179e-03	Pregnancy-specific beta-1-glycoprotein 9

C

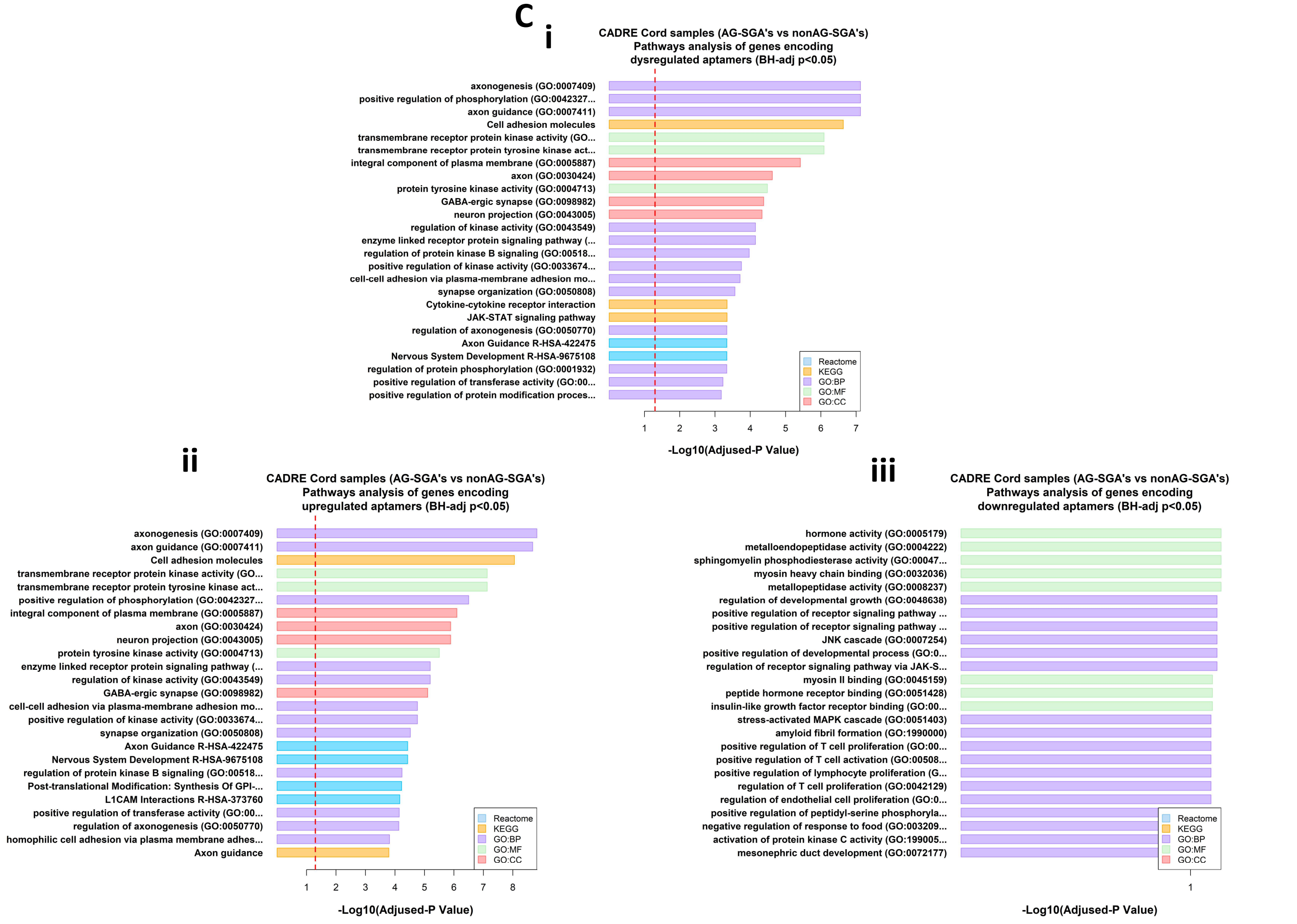

D

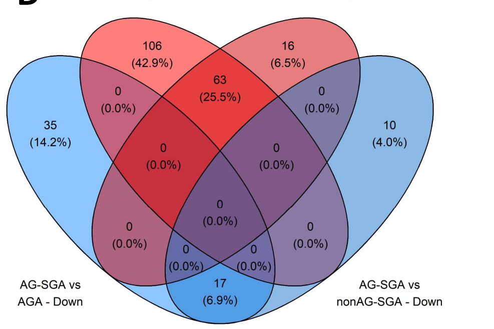

Supplementary figure 4. Differential expression analysis (limma) for the comparison between cord blood-derived proteomic profiles from subjects designated AG-SGAs (SGAs in cluster A) versus nonAG-SGAs. **A**) Volcano plot showing log_2 fold change and ($-log_{10}$) BH-adjusted p values with the top 50 significantly dysregulated aptamers labeled with their corresponding protein name. (**B**) Tabulated results of the differential expression analysis showing the top 25 dysregulated aptamers. (**C**) Horizontal bar plot displays the top 25 enriched pathways of genes encoding significantly dysregulated aptamers (BH-adj p value < 0.05). Pathways analysis was conducted on genes encoding all dysregulated aptamers (**i**), and those that were significantly higher (**ii**) and lower (**iii**) in AG-SGA individuals, separately. The x-axis shows the adjusted enrichment p value and the y-axis show the pathway terms. Bars are colored according to the repository of the enriched term. **D**) Venn diagram displaying the overlap of upregulated and downregulated aptamers from the AG-SGA versus all AGA and the AG-SGA versus nonAG-SGA comparisons.

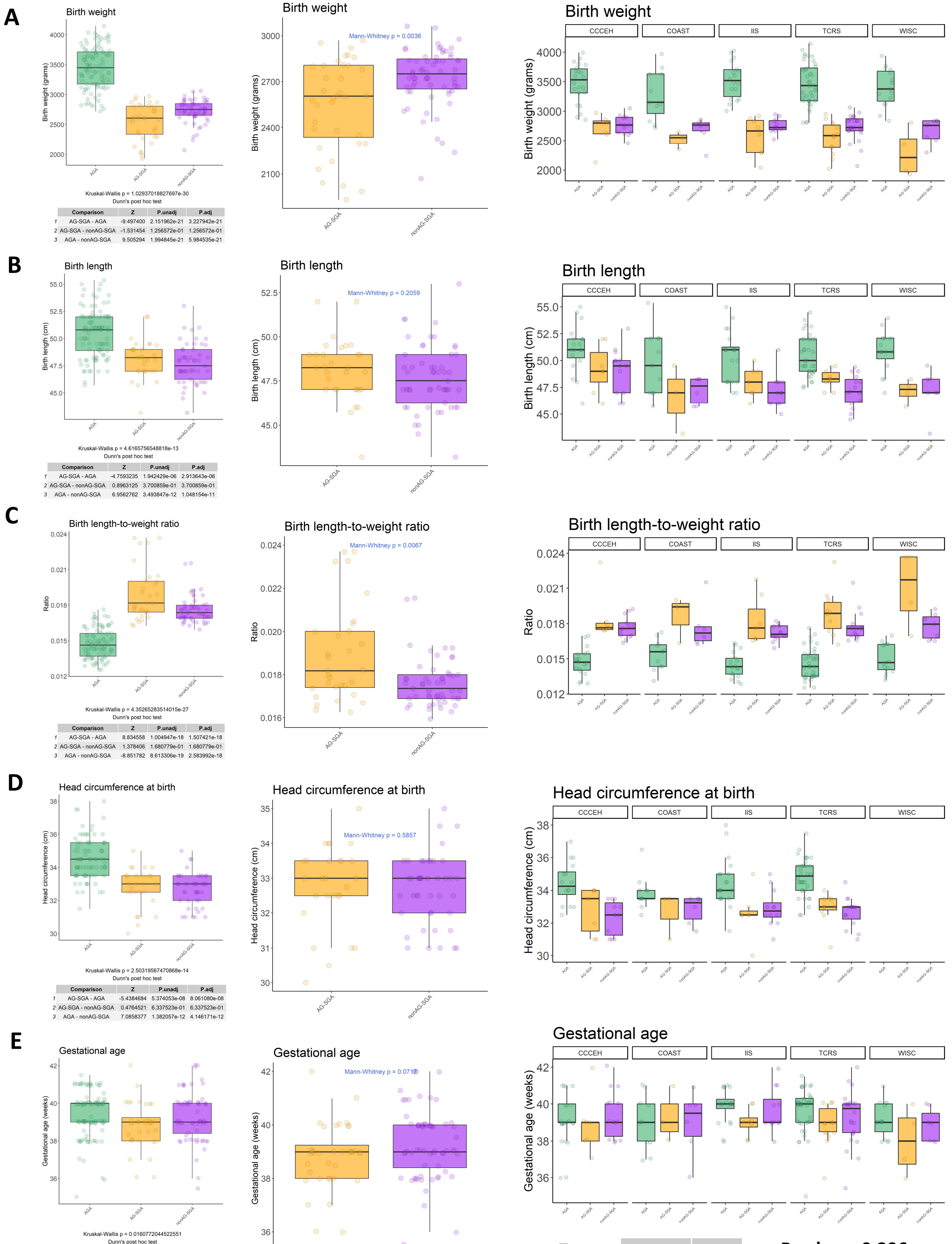

A — Birth weight. Kruskal-Wallis p = 1.02937018827697e-30. Dunn's post hoc test

	Comparison	Z	P.unadj	P.adj
1	AG-SGA - AGA	-9.497400	2.151962e-21	3.227942e-21
2	AG-SGA - nonAG-SGA	-1.531454	1.256572e-01	1.256572e-01
3	AGA - nonAG-SGA	9.505294	1.994845e-21	5.984535e-21

B — Birth length. Kruskal-Wallis p = 4.6165756548818e-13. Dunn's post hoc test

	Comparison	Z	P.unadj	P.adj
1	AG-SGA - AGA	-4.7593235	1.942429e-06	2.913643e-06
2	AG-SGA - nonAG-SGA	0.8963125	3.700859e-01	3.700859e-01
3	AGA - nonAG-SGA	6.9562762	3.493847e-12	1.048154e-11

C — Birth length-to-weight ratio. Kruskal-Wallis p = 4.35265283514015e-27. Dunn's post hoc test

	Comparison	Z	P.unadj	P.adj
1	AG-SGA - AGA	8.834558	1.004947e-18	1.507421e-18
2	AG-SGA - nonAG-SGA	1.378406	1.680779e-01	1.680779e-01
3	AGA - nonAG-SGA	-8.851782	8.613306e-19	2.583992e-18

D — Head circumference at birth. Kruskal-Wallis p = 2.50319567470868e-14. Dunn's post hoc test

	Comparison	Z	P.unadj	P.adj
1	AG-SGA - AGA	-5.4384684	5.374053e-08	8.061080e-08
2	AG-SGA - nonAG-SGA	0.4764521	6.337523e-01	6.337523e-01
3	AGA - nonAG-SGA	7.0858377	1.382057e-12	4.146171e-12

E — Gestational age. Kruskal-Wallis p = 0.0160772044522551. Dunn's post hoc test

	Comparison	Z	P.unadj	P.adj
1	AG-SGA - AGA	-2.8714655	0.004085734	0.01225720
2	AG-SGA - nonAG-SGA	-1.8940521	0.058218091	0.08732714
3	AGA - nonAG-SGA	0.9896687	0.322336059	0.32233606

F

	**Female**	**Male**
AG-SGA	16	20
nonAG-SGA	36	27

P value = 0.296

Odds ratio = 0.603
(95%CI = 0.24 – 1.48)

Supplementary figure 5. Analysis of relevant birth metrics between AGA/SGA groups. Each panel shows a three-group boxplot with statistical analysis results tabulated below (showing Kruskall-Wallis p value and Dunn's post-hoc test outputs, including 3-group multiple testing adjusted p value ['p.adj']), a two-group boxplot of AG-SGA and nonAG-SGA individuals with accompanying nominal Mann-Whitney p value for that comparison, and a three-group boxplot stratified by cohort of origin. The panels shows the analysis for birth weight (**A**), birth length (**B**) birth length-to-weight ratio (**C**), head circumference (**D**), and gestational age (**E**). The points represent individuals, with n=108 AGA, 63 nonAG-SGA, and 36 AG-SGA subjects. **F**) Table of the number of AG-SGA and nonAG-SGA samples from individuals assigned male or female at birth, and Fisher's test outputs from this contingency table.

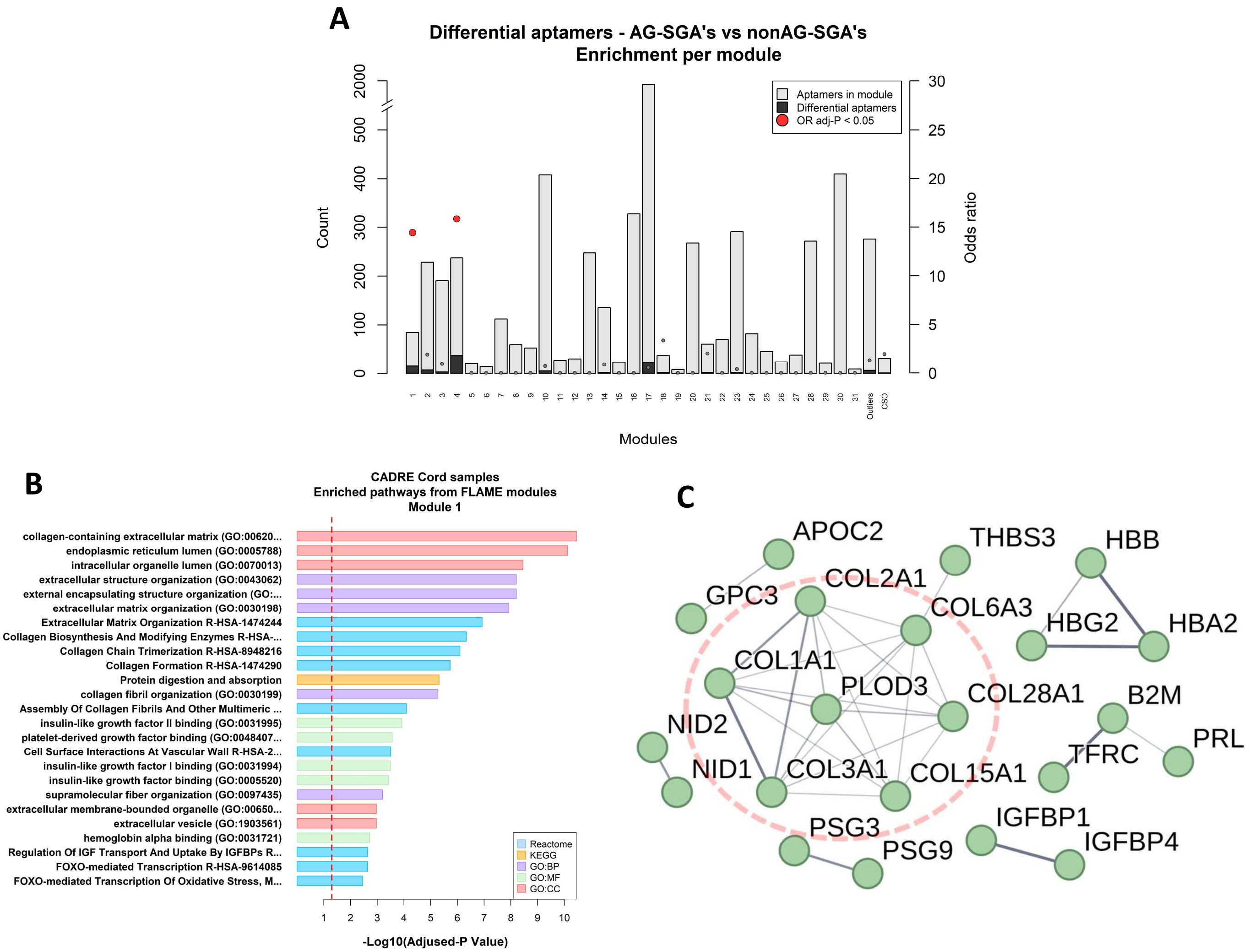

Supplementary figure 6. **A**) Stacked bar plot displaying the Fisher's test result for over-representation of significantly differentially expressed aptamers ('differential aptamers') amongst modules. The x-axis and left y-axis show the modules and number of aptamers. The black shading in each bar black indicates the number of aptamers that were significantly dysregulated from the analysis of AG-SGAs versus nonAG-SGAs for that module. The points on the plot (one per modules) correspond to the right y-axis and show the odds ratio from a Fisher's test of the enrichment of dysregulated aptamers among modules. Points colored red indicate those with a BH-adjusted-p value < 0.05 associated with the odds ratio (p value adjustment for number of modules). **B**) Horizontal bar plot showing the top 25 significantly enriched pathways from the KEGG, GO, and Reactome repositories of genes that encode Module 1 aptamers. The x-axis shows the (-log10) adjusted p value of the enrichment analysis and the y-axis displays the pathway terms. The results are order by decreasing adjusted-p value and colored according to the (sub)repository. **C**) STRING Protein-Protein Interaction (PPI) network for the aptamers (proteins) in Modules 1. Edges between nodes (proteins) represent the confidence of the connection based on prior knowledge interactions from experiments and databases. Connections were excluded if they were not at least "medium" confidence (threshold of 0.4). The dashed red circle indicates the clique of interest mentioned in the main text.

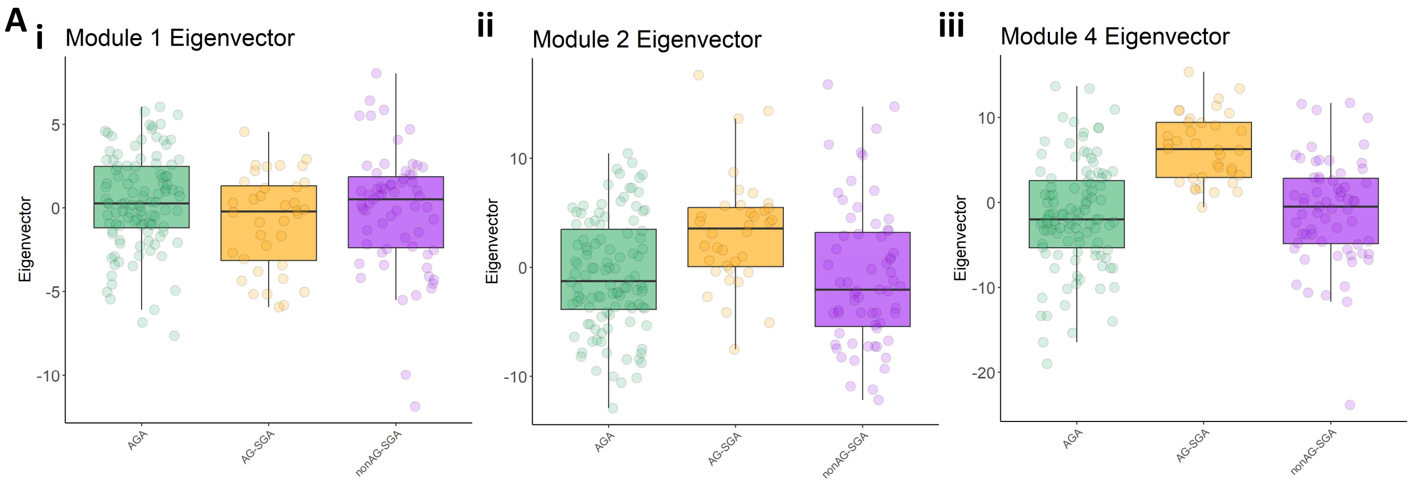

Kruskal-Wallis p = 0.20463405143892
Dunn's post hoc test

	Comparison	Z	P.unadj	P.adj
1	AG-SGA - AGA	-1.7791216	0.07521982	0.2256595
2	AG-SGA - nonAG-SGA	-1.1652913	0.24390109	0.3658516
3	AGA - nonAG-SGA	0.6240424	0.53259974	0.5325997

Kruskal-Wallis p = 0.00053503259736325
Dunn's post hoc test

	Comparison	Z	P.unadj	P.adj
1	AG-SGA - AGA	3.4658735	0.0005285118	0.0007927678
2	AG-SGA - nonAG-SGA	3.6579050	0.0002542853	0.0007628559
3	AGA - nonAG-SGA	0.6133167	0.5396669720	0.5396669720

Kruskal-Wallis p = 1.32635019300311e-10
Dunn's post hoc test

	Comparison	Z	P.unadj	P.adj
1	AG-SGA - AGA	6.5855613	4.531701e-11	1.359510e-10
2	AG-SGA - nonAG-SGA	5.6016426	2.123300e-08	3.184950e-08
3	AGA - nonAG-SGA	-0.6122023	5.404039e-01	5.404039e-01

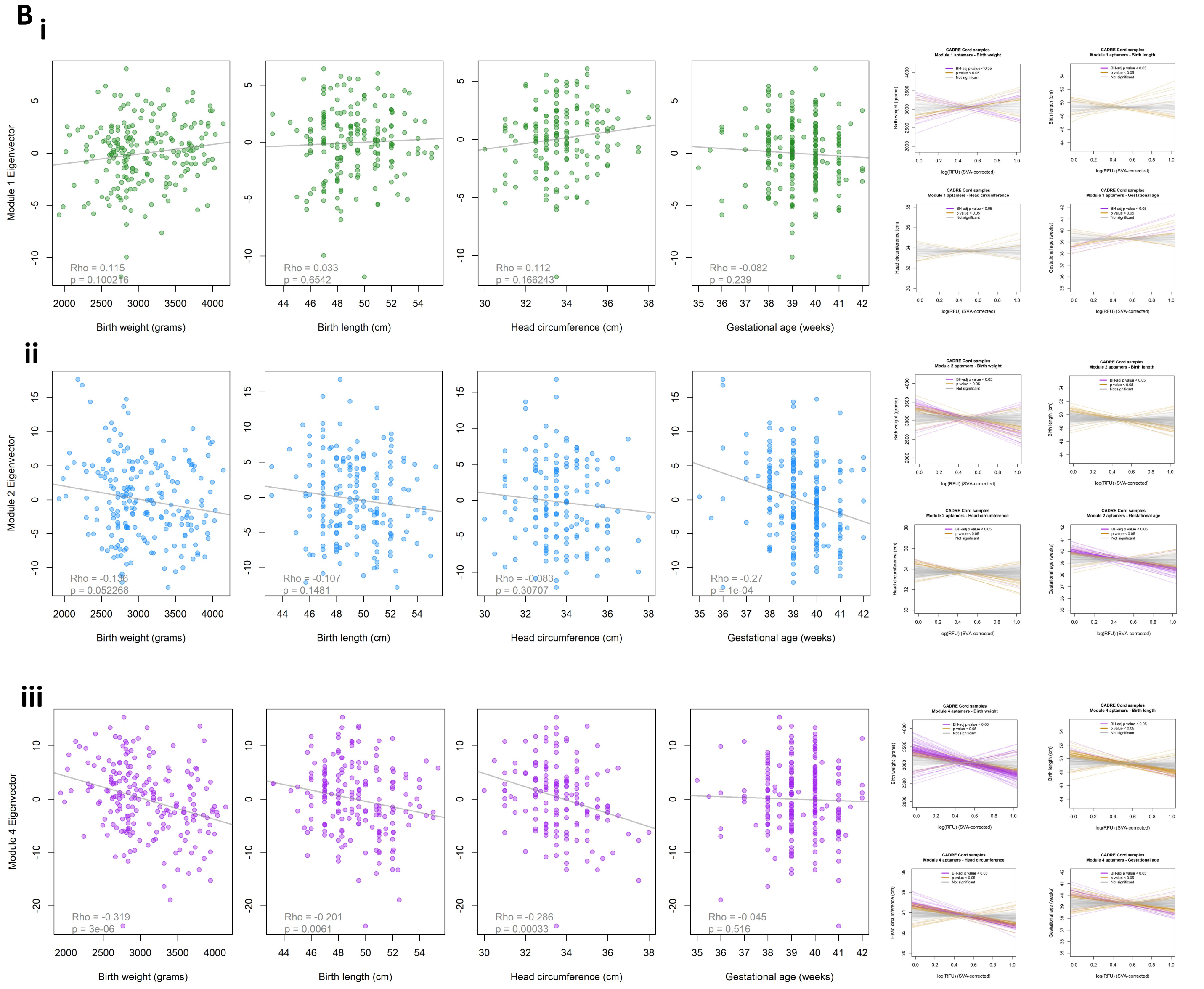

Supplementary figure 7. Analysis of module eigenvectors (first principal component) calculated from SVA-adjusted data with relevant birth variables. **A**) Boxplots of Module 1 (**i**), 2 (**ii**), and 4 (**iii**) eigenvectors stratified by AGA/SGA group, with accompanying statistical analysis (showing Kruskall-Wallis p value and Dunn's post-hoc test outputs, including 3-group multiple testing adjusted p value ['p.adj']) (below). The points on the boxplots represent individuals, (n = 108 AGA, 63 nonAG-SGA, and 36 AG-SGA subjects). **B**) Correlation plots between Module 1 (**i**), 2 (**ii**), and 4 (**iii**) eigenvectors and birth weight, length, head circumference, and gestational age. The Spearman's rho and p value for each correlation is shown in grey text. Accompanying the correlation plots of the eigenvectors are plots of the line-of-best-fit for each aptamer in that module compared to birth variables, colored by the strength of its Spearman's p value. This is included to demonstrate the relative number of individual aptamers in each module that show a significant correlation.

A

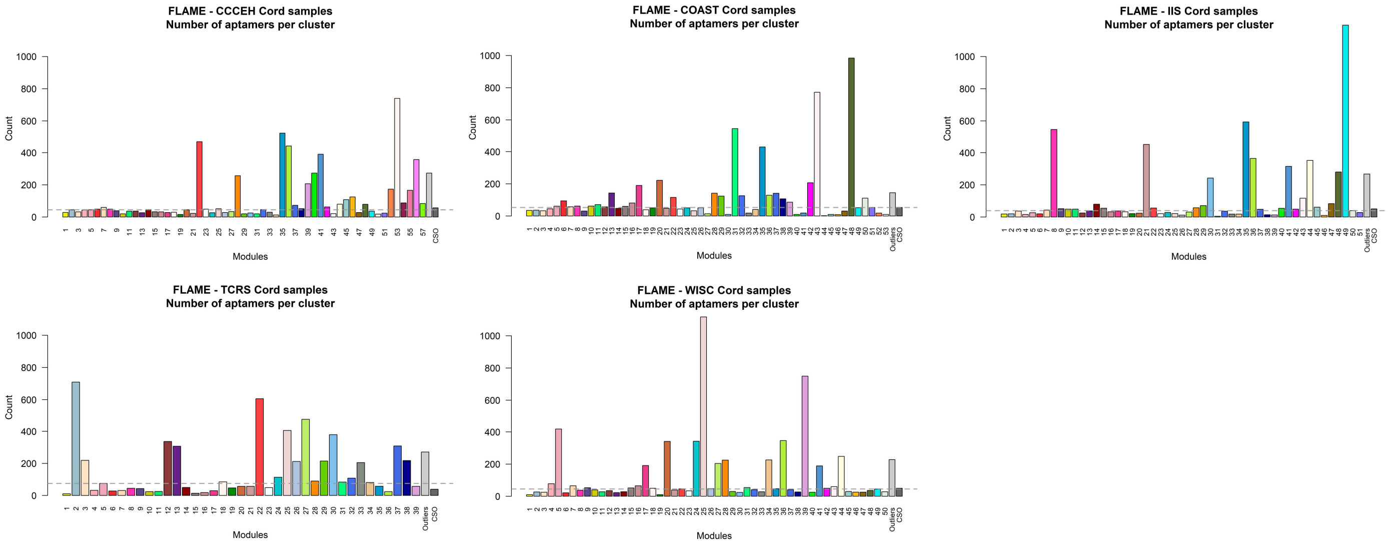
FLAME - CCCEH Cord samples
Number of aptamers per cluster
FLAME - COAST Cord samples
Number of aptamers per cluster
FLAME - IIS Cord samples
Number of aptamers per cluster
FLAME - TCRS Cord samples
Number of aptamers per cluster
FLAME - WISC Cord samples
Number of aptamers per cluster
Count
Modules
Outliers
CSO

B

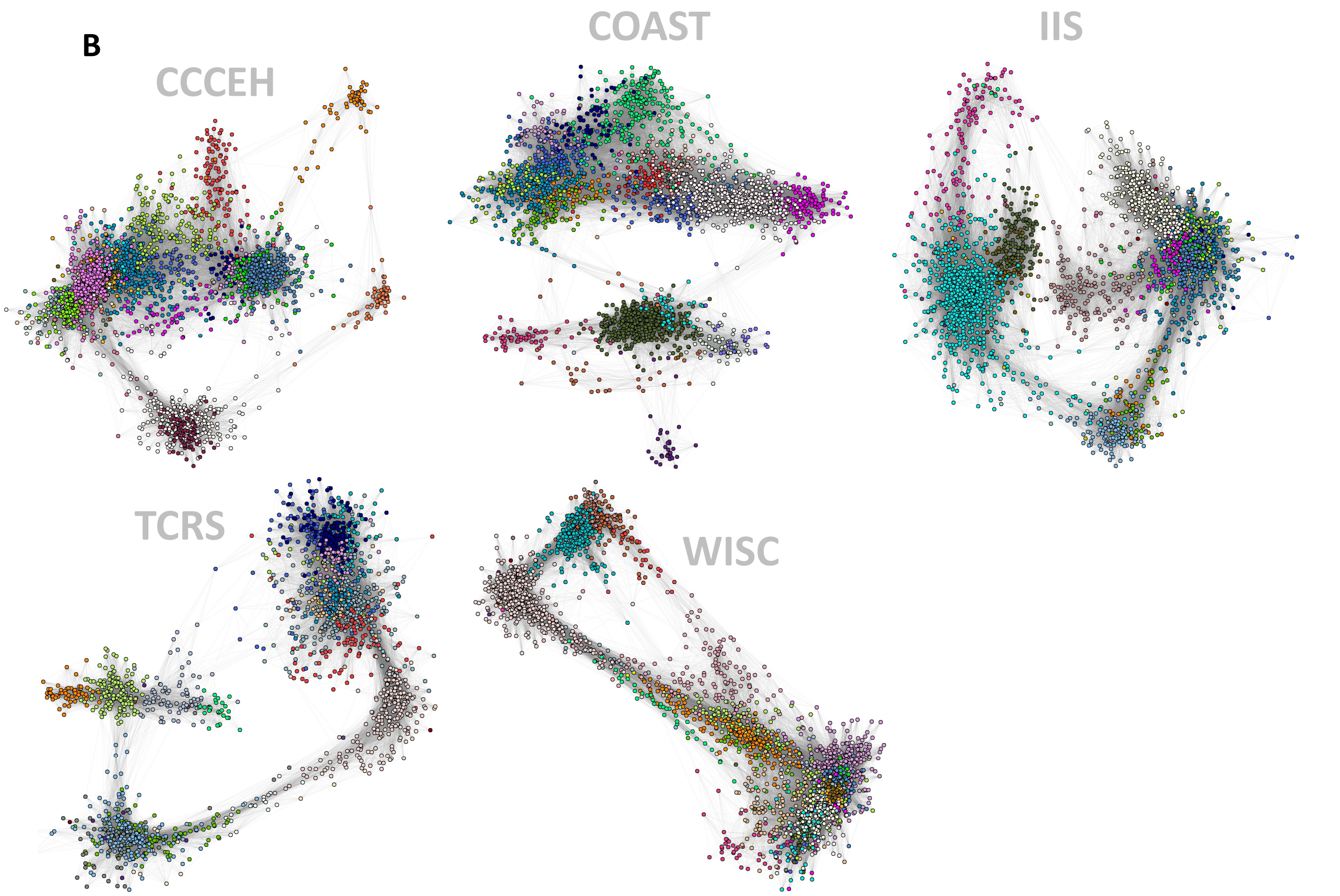
CCCEH
COAST
IIS
TCRS
WISC

Supplementary figure 8. FLAME network analysis of each of the five CADRE cohort separately. **A**) Bar plots displaying the number of aptamers (y-axis) per cluster identified by FLAME network analysis for each cohort. The x-axis shows the number of clusters of functionally-related aptamers, as well as a cluster of outliers deemed unrelated to any cluster and the Cluster Specific Objects (CSO). Representative (cosine) network wiring diagrams demonstrating the intra- and inter-module connections amongst aptamers, overlaid with the FLAME analysis assigned module, for each cohort. For visualization purposes, edges were filtered to those with a Pearson's correlation > 0.75 and nodes were excluded if they had degree < 20 for CCCEH and TCRS, < 30 for IIS and WISC, and < 40 for COAST. The node/module color corresponds to the bars in (**A**), which serves as a legend.

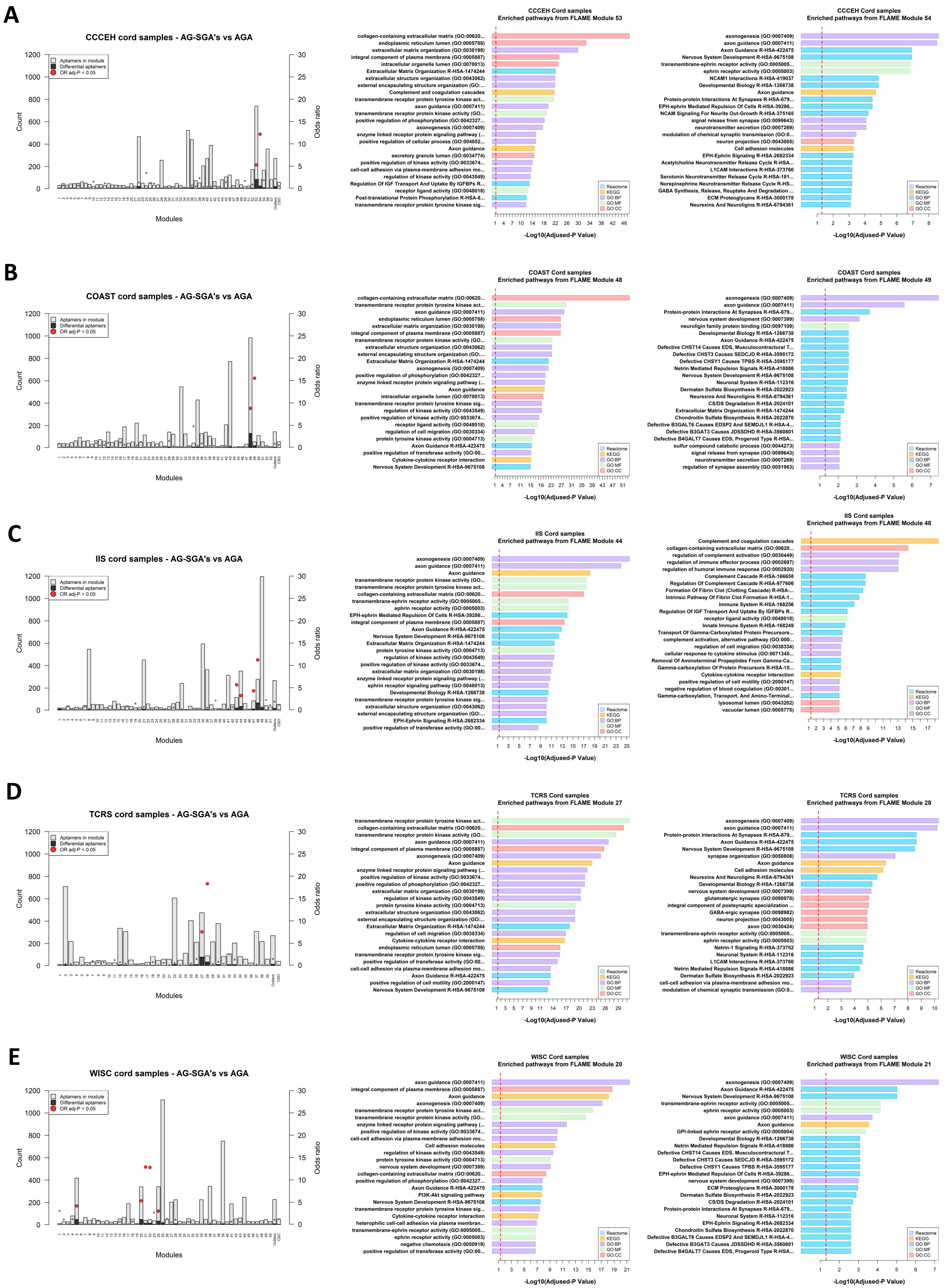
A
CCCEH cord samples - AG-SGA's vs AGA
Aptamers in module
Differential aptamers
OR adj-P < 0.05
Count
Odds ratio
Modules
CCCEH Cord samples
Enriched pathways from FLAME Module 53
collagen-containing extracellular matrix (GO:00620...
endoplasmic reticulum lumen (GO:0005788)
extracellular matrix organization (GO:0030198)
integral component of plasma membrane (GO:0005887)
intracellular organelle lumen (GO:0070013)
Extracellular Matrix Organization R-HSA-1474244
extracellular structure organization (GO:0043062)
external encapsulating structure organization (GO:...
Complement and coagulation cascades
transmembrane receptor protein tyrosine kinase act...
axon guidance (GO:0007411)
transmembrane receptor protein kinase activity (GO...
positive regulation of phosphorylation (GO:0042327...
axonogenesis (GO:0007409)
enzyme linked receptor protein signaling pathway (...
positive regulation of cellular process (GO:004852...
Axon guidance
secretory granule lumen (GO:0034774)
positive regulation of kinase activity (GO:0033674...
cell-cell adhesion via plasma-membrane adhesion mo...
regulation of kinase activity (GO:0043549)
Regulation Of IGF Transport And Uptake By IGFBPs R...
receptor ligand activity (GO:0048018)
Post-translational Protein Phosphorylation R-HSA-8...
transmembrane receptor protein tyrosine kinase sig...
-Log10(Adjused-P Value)
CCCEH Cord samples
Enriched pathways from FLAME Module 54
axonogenesis (GO:0007409)
axon guidance (GO:0007411)
Axon Guidance R-HSA-422475
Nervous System Development R-HSA-9675108
transmembrane-ephrin receptor activity (GO:0005005...
ephrin receptor activity (GO:0005003)
NCAM1 Interactions R-HSA-419037
Developmental Biology R-HSA-1266738
Axon guidance
Protein-protein Interactions At Synapses R-HSA-679...
EPH-ephrin Mediated Repulsion Of Cells R-HSA-39286...
NCAM Signaling For Neurite Out-Growth R-HSA-375165
signal release from synapse (GO:0099643)
neurotransmitter secretion (GO:0007269)
modulation of chemical synaptic transmission (GO:0...
neuron projection (GO:0043005)
Cell adhesion molecules
EPH-Ephrin Signaling R-HSA-2682334
Acetylcholine Neurotransmitter Release Cycle R-HSA...
L1CAM Interactions R-HSA-373760
Serotonin Neurotransmitter Release Cycle R-HSA-181...
Norepinephrine Neurotransmitter Release Cycle R-HS...
GABA Synthesis, Release, Reuptake And Degradation ...
ECM Proteoglycans R-HSA-3000178
Neurexins And Neuroligins R-HSA-6794361
Reactome
KEGG
GO:BP
GO:MF
GO:CC
-Log10(Adjused-P Value)
B
COAST cord samples - AG-SGA's vs AGA
Count
Odds ratio
Modules
COAST Cord samples
Enriched pathways from FLAME Module 48
collagen-containing extracellular matrix (GO:00620...
transmembrane receptor protein tyrosine kinase act...
axon guidance (GO:0007411)
endoplasmic reticulum lumen (GO:0005788)
extracellular matrix organization (GO:0030198)
integral component of plasma membrane (GO:0005887)
transmembrane receptor protein kinase activity (GO...
extracellular structure organization (GO:0043062)
external encapsulating structure organization (GO:...
Extracellular Matrix Organization R-HSA-1474244
axonogenesis (GO:0007409)
positive regulation of phosphorylation (GO:0042327...
enzyme linked receptor protein signaling pathway (...
Axon guidance
intracellular organelle lumen (GO:0070013)
transmembrane receptor protein tyrosine kinase sig...
regulation of kinase activity (GO:0043549)
positive regulation of kinase activity (GO:0033674...
receptor ligand activity (GO:0048018)
regulation of cell migration (GO:0030334)
protein tyrosine kinase activity (GO:0004713)
Axon Guidance R-HSA-422475
positive regulation of transferase activity (GO:00...
Cytokine-cytokine receptor interaction
Nervous System Development R-HSA-9675108
-Log10(Adjused-P Value)
COAST Cord samples
Enriched pathways from FLAME Module 49
axonogenesis (GO:0007409)
axon guidance (GO:0007411)
Protein-protein Interactions At Synapses R-HSA-679...
nervous system development (GO:0007399)
neuroligin family protein binding (GO:0097109)
Developmental Biology R-HSA-1266738
Axon Guidance R-HSA-422475
Defective CHST14 Causes EDS, Musculocontractural T...
Defective CHST3 Causes SEDCJD R-HSA-3595172
Defective CHSY1 Causes TPBS R-HSA-3595177
Netrin Mediated Repulsion Signals R-HSA-418886
Nervous System Development R-HSA-9675108
Neuronal System R-HSA-112316
Dermatan Sulfate Biosynthesis R-HSA-2022923
Neurexins And Neuroligins R-HSA-6794361
CS/DS Degradation R-HSA-2024101
Extracellular Matrix Organization R-HSA-1474244
Chondroitin Sulfate Biosynthesis R-HSA-2022870
Defective B3GALT6 Causes EDSP2 And SEMDJL1 R-HSA-4...
Defective B3GAT3 Causes JDSSDHD R-HSA-3560801
Defective B4GALT7 Causes EDS, Progeroid Type R-HSA...
sulfur compound catabolic process (GO:0044273)
signal release from synapse (GO:0099643)
neurotransmitter secretion (GO:0007269)
regulation of synapse assembly (GO:0051963)
-Log10(Adjused-P Value)
C
IIS cord samples - AG-SGA's vs AGA
Count
Odds ratio
Modules
IIS Cord samples
Enriched pathways from FLAME Module 44
axonogenesis (GO:0007409)
axon guidance (GO:0007411)
Axon guidance
transmembrane receptor protein kinase activity (GO...
transmembrane receptor protein tyrosine kinase act...
collagen-containing extracellular matrix (GO:00620...
transmembrane-ephrin receptor activity (GO:0005005...
ephrin receptor activity (GO:0005003)
EPH-ephrin Mediated Repulsion Of Cells R-HSA-39286...
integral component of plasma membrane (GO:0005887)
Axon Guidance R-HSA-422475
Nervous System Development R-HSA-9675108
Extracellular Matrix Organization R-HSA-1474244
protein tyrosine kinase activity (GO:0004713)
regulation of kinase activity (GO:0043549)
positive regulation of kinase activity (GO:0033674...
extracellular matrix organization (GO:0030198)
enzyme linked receptor protein signaling pathway (...
ephrin receptor signaling pathway (GO:0048013)
Developmental Biology R-HSA-1266738
transmembrane receptor protein tyrosine kinase sig...
extracellular structure organization (GO:0043062)
external encapsulating structure organization (GO:...
EPH-Ephrin Signaling R-HSA-2682334
positive regulation of transferase activity (GO:00...
-Log10(Adjused-P Value)
IIS Cord samples
Enriched pathways from FLAME Module 48
Complement and coagulation cascades
collagen-containing extracellular matrix (GO:00620...
regulation of complement activation (GO:0030449)
regulation of immune effector process (GO:0002697)
regulation of humoral immune response (GO:0002920)
Complement Cascade R-HSA-166658
Regulation Of Complement Cascade R-HSA-977606
Formation Of Fibrin Clot (Clotting Cascade) R-HSA-...
Intrinsic Pathway Of Fibrin Clot Formation R-HSA-1...
Immune System R-HSA-168256
Regulation Of IGF Transport And Uptake By IGFBPs R...
receptor ligand activity (GO:0048018)
Innate Immune System R-HSA-168249
Transport Of Gamma-Carboxylated Protein Precursors...
complement activation, alternative pathway (GO:000...
regulation of cell migration (GO:0030334)
cellular response to cytokine stimulus (GO:0071345...
Removal Of Aminoterminal Propeptides From Gamma-Ca...
Gamma-carboxylation Of Protein Precursors R-HSA-15...
Cytokine-cytokine receptor interaction
positive regulation of cell motility (GO:2000147)
negative regulation of blood coagulation (GO:00301...
Gamma-carboxylation, Transport, And Amino-Terminal...
lysosomal lumen (GO:0043202)
vacuolar lumen (GO:0005775)
-Log10(Adjused-P Value)
D
TCRS cord samples - AG-SGA's vs AGA
Count
Odds ratio
Modules
TCRS Cord samples
Enriched pathways from FLAME Module 27
transmembrane receptor protein tyrosine kinase act...
collagen-containing extracellular matrix (GO:00620...
transmembrane receptor protein kinase activity (GO...
axon guidance (GO:0007411)
integral component of plasma membrane (GO:0005887)
axonogenesis (GO:0007409)
Axon guidance
enzyme linked receptor protein signaling pathway (...
positive regulation of kinase activity (GO:0033674...
positive regulation of phosphorylation (GO:0042327...
extracellular matrix organization (GO:0030198)
regulation of kinase activity (GO:0043549)
protein tyrosine kinase activity (GO:0004713)
extracellular structure organization (GO:0043062)
external encapsulating structure organization (GO:...
Extracellular Matrix Organization R-HSA-1474244
regulation of cell migration (GO:0030334)
Cytokine-cytokine receptor interaction
endoplasmic reticulum lumen (GO:0005788)
transmembrane receptor protein tyrosine kinase sig...
positive regulation of transferase activity (GO:00...
cell-cell adhesion via plasma-membrane adhesion mo...
Axon Guidance R-HSA-422475
positive regulation of cell motility (GO:2000147)
Nervous System Development R-HSA-9675108
-Log10(Adjused-P Value)
TCRS Cord samples
Enriched pathways from FLAME Module 28
axonogenesis (GO:0007409)
axon guidance (GO:0007411)
Protein-protein Interactions At Synapses R-HSA-679...
Axon Guidance R-HSA-422475
Nervous System Development R-HSA-9675108
synapse organization (GO:0050808)
Axon guidance
Cell adhesion molecules
Neurexins And Neuroligins R-HSA-6794361
Developmental Biology R-HSA-1266738
nervous system development (GO:0007399)
glutamatergic synapse (GO:0098978)
integral component of postsynaptic specialization ...
GABA-ergic synapse (GO:0098982)
neuron projection (GO:0043005)
axon (GO:0030424)
transmembrane-ephrin receptor activity (GO:0005005...
ephrin receptor activity (GO:0005003)
Netrin-1 Signaling R-HSA-373752
Neuronal System R-HSA-112316
L1CAM Interactions R-HSA-373760
Netrin Mediated Repulsion Signals R-HSA-418886
Dermatan Sulfate Biosynthesis R-HSA-2022923
cell-cell adhesion via plasma-membrane adhesion mo...
modulation of chemical synaptic transmission (GO:0...
-Log10(Adjused-P Value)
E
WISC cord samples - AG-SGA's vs AGA
Count
Odds ratio
Modules
WISC Cord samples
Enriched pathways from FLAME Module 20
axon guidance (GO:0007411)
integral component of plasma membrane (GO:0005887)
Axon guidance
axonogenesis (GO:0007409)
transmembrane receptor protein tyrosine kinase act...
transmembrane receptor protein kinase activity (GO...
enzyme linked receptor protein signaling pathway (...
positive regulation of kinase activity (GO:0033674...
cell-cell adhesion via plasma-membrane adhesion mo...
Cell adhesion molecules
regulation of kinase activity (GO:0043549)
protein tyrosine kinase activity (GO:0004713)
nervous system development (GO:0007399)
collagen-containing extracellular matrix (GO:00620...
positive regulation of phosphorylation (GO:0042327...
Axon Guidance R-HSA-422475
PI3K-Akt signaling pathway
Nervous System Development R-HSA-9675108
transmembrane receptor protein tyrosine kinase sig...
Cytokine-cytokine receptor interaction
heterophilic cell-cell adhesion via plasma membran...
transmembrane-ephrin receptor activity (GO:0005005...
ephrin receptor activity (GO:0005003)
negative chemotaxis (GO:0050919)
positive regulation of transferase activity (GO:00...
-Log10(Adjused-P Value)
WISC Cord samples
Enriched pathways from FLAME Module 21
axonogenesis (GO:0007409)
Axon Guidance R-HSA-422475
Nervous System Development R-HSA-9675108
transmembrane-ephrin receptor activity (GO:0005005...
ephrin receptor activity (GO:0005003)
axon guidance (GO:0007411)
Axon guidance
GPI-linked ephrin receptor activity (GO:0005004)
Developmental Biology R-HSA-1266738
Netrin Mediated Repulsion Signals R-HSA-418886
Defective CHST14 Causes EDS, Musculocontractural T...
Defective CHST3 Causes SEDCJD R-HSA-3595172
Defective CHSY1 Causes TPBS R-HSA-3595177
EPH-ephrin Mediated Repulsion Of Cells R-HSA-39286...
nervous system development (GO:0007399)
ECM Proteoglycans R-HSA-3000178
Dermatan Sulfate Biosynthesis R-HSA-2022923
CS/DS Degradation R-HSA-2024101
Protein-protein Interactions At Synapses R-HSA-679...
Neuronal System R-HSA-112316
EPH-Ephrin Signaling R-HSA-2682334
Chondroitin Sulfate Biosynthesis R-HSA-2022870
Defective B3GALT6 Causes EDSP2 And SEMDJL1 R-HSA-4...
Defective B3GAT3 Causes JDSSDHD R-HSA-3560801
Defective B4GALT7 Causes EDS, Progeroid Type R-HSA...
-Log10(Adjused-P Value)

Supplementary figure 9. FLAME network analysis of each of the five CADRE cohort separately. Panels show the stacked bar plot of over-representation of aptamers dysregulated in AG-SGAs and pathway enrichment results for selected modules for the CCCEH (**A**), COAST (**B**), IIS, (**C**), TCRS (**D**), and WISC (**E**) cohorts. The stacked bar plots display the Fisher's test result for over-representation of significantly differentially expressed aptamers ('differential aptamers') amongst modules. The x-axis and left y-axis show the modules and number of aptamers. The black shading in each bar black indicates the number of aptamers that were significantly dysregulated from the analysis of all AG-SGAs versus all AGAs for that module. The points on the plot (one per module) correspond to the right y-axis and show the odd ratio from a Fisher's test of the enrichment of dysregulated aptamers among modules. Points colored red indicate those with a BH-adjusted-p value < 0.05 associated with the odds ratio (p value adjustment for number of modules). Horizontal bar plot showing the top 25 significantly enriched pathways from the KEGG, GO, and Reactome repositories of genes that encode selected module aptamers. The x-axis shows the (-log10) adjusted p value of the enrichment analysis and the y-axis displays the pathway terms. The results are order by decreasing adjusted-p value and colored according to the (sub)repository.

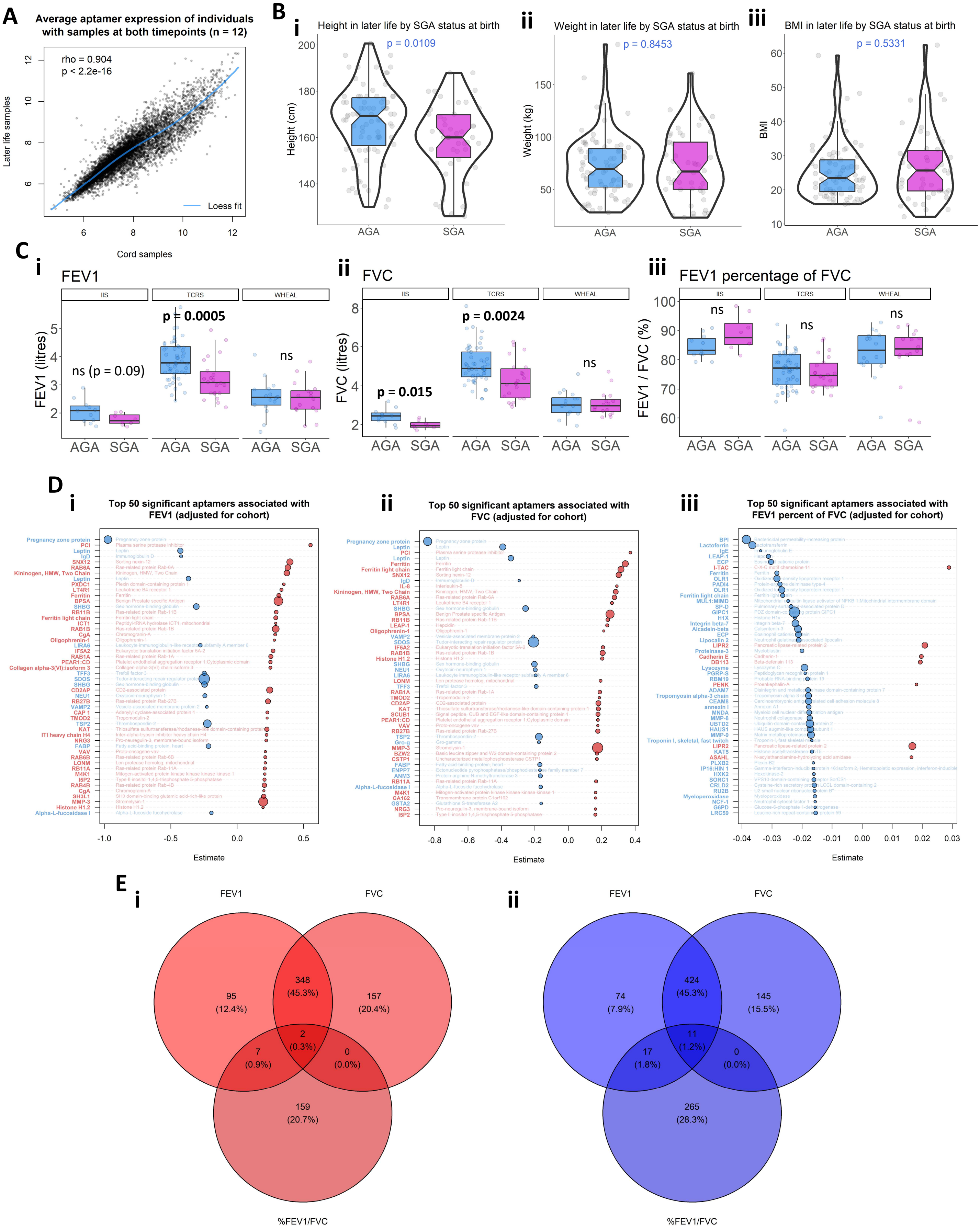

A
Average aptamer expression of individuals with samples at both timepoints (n = 12)
rho = 0.904
p < 2.2e-16
Loess fit
Later life samples
Cord samples
B
i
Height in later life by SGA status at birth
p = 0.0109
Height (cm)
ii
Weight in later life by SGA status at birth
p = 0.8453
Weight (kg)
iii
BMI in later life by SGA status at birth
p = 0.5331
BMI
AGA
SGA
C
i
FEV1
FEV1 (litres)
ns (p = 0.09)
p = 0.0005
ns
ii
FVC
FVC (litres)
p = 0.015
p = 0.0024
ns
iii
FEV1 percentage of FVC
FEV1 / FVC (%)
ns
IIS
TCRS
WHEAL
D
i
Top 50 significant aptamers associated with FEV1 (adjusted for cohort)
ii
Top 50 significant aptamers associated with FVC (adjusted for cohort)
iii
Top 50 significant aptamers associated with FEV1 percent of FVC (adjusted for cohort)
Estimate
E
i
FEV1
FVC
%FEV1/FVC
95 (12.4%)
348 (45.3%)
157 (20.4%)
2 (0.3%)
7 (0.9%)
0 (0.0%)
159 (20.7%)
ii
FEV1
FVC
%FEV1/FVC
74 (7.9%)
424 (45.3%)
145 (15.5%)
11 (1.2%)
17 (1.8%)
0 (0.0%)
265 (28.3%)

Supplementary figure 10. Analysis of height, weight, and BMI, and spirometry measurements of subjects with a blood sample available in later life. **A**) Correlation plot of average aptamer expression for the 12 individuals with samples available from cord blood (x-axis) and later life peripheral blood (y-axis) collection timepoints. Each point represents an individual aptamer (n = 7,133) and the blue line represents a loess fit of the data. The Spearman's Rho and p value for the association are shown in the top left corner. **B**) Violin plots of later life Height (**i**), weight (**ii**), and BMI (**iii**), stratified by SGA status at birth. Violin plots have the same parameters as those in Figure 3A & B in the main text, showing the two-group Mann-Whitney p value for each comparison. The points represent individuals (n = 80 AGA and 46 SGA subjects) **C**) Boxplots of FEV_1 (**i**), FVC (**ii**), and the FEV_1 percentage of FVC (**iii**), stratified by SGA status at birth and split by cohort. Points on the plot represent individuals, with the following samples sizes per cohort; IIS 11 AGA / 8 SGA, TCRS 53 AGA / 22 SGA, and WHEAL 16 AGA, 16 SGA. Associated two-group Mann-Whitney p values are displayed on the plot for each group-wise comparison. **D**) Top 50 aptamers identified in association with FEV_1 (**i**), FVC (**ii**), and percentage of FEV_1 of FVC (%) (**iii**) from linear models adjusting for cohort. The x-axis shows the model estimate for each comparison and the y-axis shows the protein name corresponding to the aptamers. Results are order by decreasing estimate, points are colored red/blue depending on the significant positive/negative association, and point size is proportional to the (nominal) p value associated with the spirometry measurement (adjusted for cohort). The unabbreviated name of proteins corresponding to aptamers is shown within each plot. **E**) Venn diagrams displaying the overlap in aptamers which showed significantly positively (**i**) and negatively (**ii**) association with FEV_1, FVC, and $\%FEV_1/FVC$.

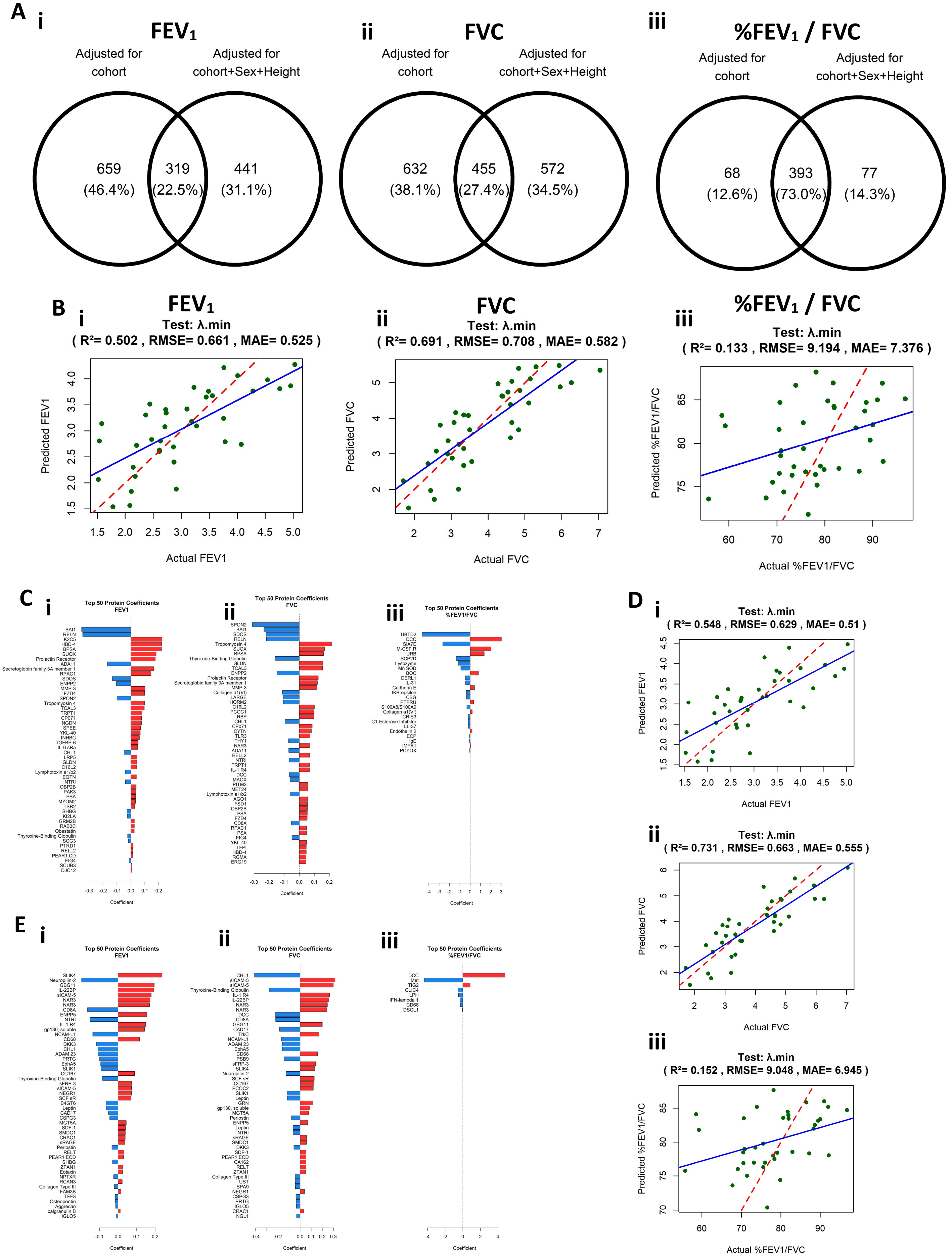

A
i FEV1
Adjusted for cohort
Adjusted for cohort+Sex+Height
659 (46.4%)
319 (22.5%)
441 (31.1%)
ii FVC
632 (38.1%)
455 (27.4%)
572 (34.5%)
iii %FEV1 / FVC
68 (12.6%)
393 (73.0%)
77 (14.3%)
B
i FEV1
Test: λ.min
(R²= 0.502 , RMSE= 0.661 , MAE= 0.525)
Predicted FEV1
Actual FEV1
ii FVC
Test: λ.min
(R²= 0.691 , RMSE= 0.708 , MAE= 0.582)
Predicted FVC
Actual FVC
iii %FEV1 / FVC
Test: λ.min
(R²= 0.133 , RMSE= 9.194 , MAE= 7.376)
Predicted %FEV1/FVC
Actual %FEV1/FVC
C
i Top 50 Protein Coefficients FEV1
ii Top 50 Protein Coefficients FVC
iii Top 50 Protein Coefficients %FEV1/FVC
Coefficient
D
i Test: λ.min
(R²= 0.548 , RMSE= 0.629 , MAE= 0.51)
Predicted FEV1
Actual FEV1
ii Test: λ.min
(R²= 0.731 , RMSE= 0.663 , MAE= 0.555)
Predicted FVC
Actual FVC
iii Test: λ.min
(R²= 0.152 , RMSE= 9.048 , MAE= 6.945)
Predicted %FEV1/FVC
Actual %FEV1/FVC
E
i Top 50 Protein Coefficients FEV1
ii Top 50 Protein Coefficients FVC
iii Top 50 Protein Coefficients %FEV1/FVC
Coefficient

Supplementary figure 11. Analysis of linear and multivariate model results of aptamer association with FEV_1, FVC, and FEV_1 percentage of FVC. **A**) Venn diagram of the overlap of significant aptamers (nominal $p < 0.05$) between analyses adjusting for cohort and cohort+sex+height for aptamer correlations with FEV_1 (**i**), FVC (**ii**), and the FEV_1 percentage of FVC (**iii**). **B**) Plots demonstrating the predictive performance on the test set (30% of total sample not used in model training) of elastic net regression models on FEV_1 (**i**), FVC (**ii**) and %FEV_1/FVC (**iii**) from all aptamers in later life peripheral blood protein profiles (with cohort, sex, and height as covariates). The x-axis shows the actual measurement for the samples, and the y-axis shows the predicted values. The red dashed line represent the reference line of perfect prediction (slope 1, intercept 0), and the solid blue line represents the best fit regression. Relevant prediction metrics are displayed in the plot titles; R2, R-squared; RMSE, Root Mean Squared Error; MAE, Mean Absolute Error. **C**) Top protein coefficients for elastic net regression models of FEV_1 (**i**), FVC (**ii**) and %FEV_1/FVC (**iii**) from all aptamers in later life peripheral blood protein profiles (i.e., those shown in (B)). **D**) Same plot parameters as (B) for models restricted to the later life peripheral blood aptamer counterparts identified from the AG-SGA versus AGA analysis of cord blood-derived protein profiles (n=221, shown in Figure 1B(i)). **E**) Same plot parameters as (C) showing top protein coefficients from the models restricted to the aptamers identified from the AG-SGA versus AGA analysis of cord blood-derived protein profiles. Related to (D).

A

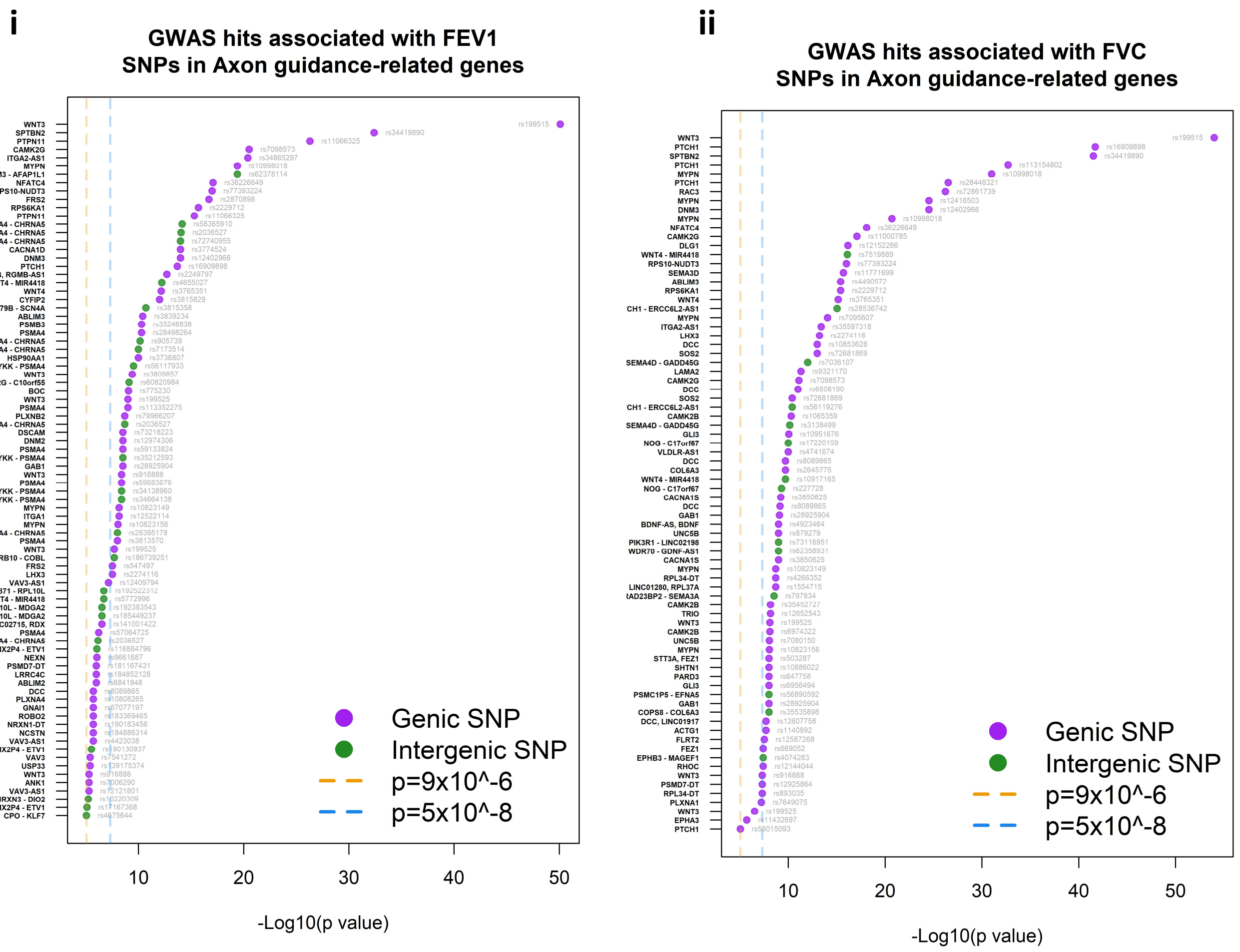

B

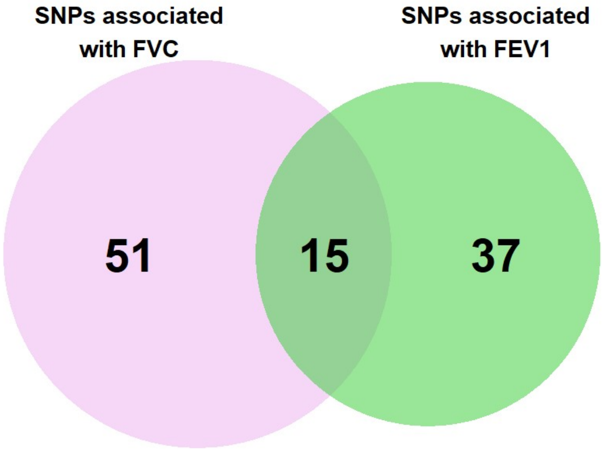

Contingency table

	Signif. in FEV1	Not signif. in FEV1
Signif. in FVC	15	51
Not signif. in FVC	37	3236

Fisher's test

	p value	Odds ratio	95% confidance interval range
Fisher's test	1.325×10^{-14}	25.59	12.26 - 51.42

Supplementary figure 12. Meta-analysis of SNPs associated with FEV_1 and FVC. **A**) Plots of all SNPs significantly associated with axon guidance related genes for FEV_1 (**i**) and FVC (**ii**). The x-axis shows the -Log10(p value) of the association and the y-axis shows the mapped gene(s). Points are colored according whether they are genic or intergenic SNPs and the SNP identifier is shown in grey text alongside each point. The dashed orange and blue vertical line represent the p values of $9x10^{-6}$ and $5x10^{-8}$, respectively. **B**) Venn diagram (left) showing the overlap of SNPs associated with FEV_1 (green) and FVC (pink) at genome wide significance ($p < 5x10^{-8}$). The numbers displayed exclude duplicate SNP identifiers. Contingency table and Fisher's test results (right) test whether the overlap is greater (or less) than expected by chance. Contingency table shows the number of SNPs that were and were not significant from the total pool of overlapping SNPs.

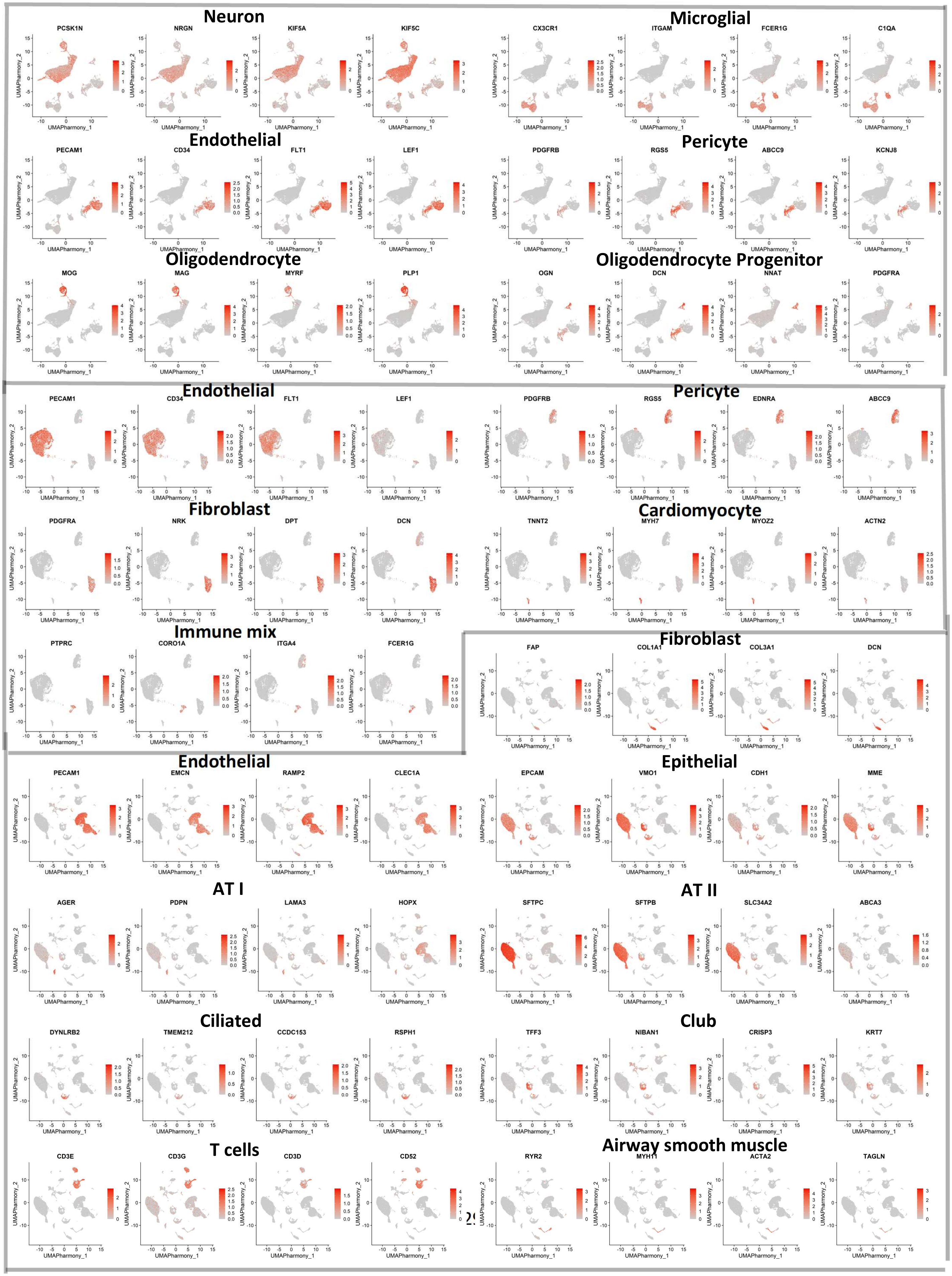
Neuron
PCSK1N
NRGN
KIF5A
KIF5C
Microglial
CX3CR1
ITGAM
FCER1G
C1QA
Endothelial
PECAM1
CD34
FLT1
LEF1
Pericyte
PDGFRB
RGS5
ABCC9
KCNJ8
Oligodendrocyte
MOG
MAG
MYRF
PLP1
Oligodendrocyte Progenitor
OGN
DCN
NNAT
PDGFRA
Endothelial
PECAM1
CD34
FLT1
LEF1
Pericyte
PDGFRB
RGS5
EDNRA
ABCC9
Fibroblast
PDGFRA
NRK
DPT
DCN
Cardiomyocyte
TNNT2
MYH7
MYOZ2
ACTN2
Immune mix
PTPRC
CORO1A
ITGA4
FCER1G
Fibroblast
FAP
COL1A1
COL3A1
DCN
Endothelial
PECAM1
EMCN
RAMP2
CLEC1A
Epithelial
EPCAM
VMO1
CDH1
MME
AT I
AGER
PDPN
LAMA3
HOPX
AT II
SFTPC
SFTPB
SLC34A2
ABCA3
Ciliated
DYNLRB2
TMEM212
CCDC153
RSPH1
Club
TFF3
NIBAN1
CRISP3
KRT7
T cells
CD3E
CD3G
CD3D
CD52
Airway smooth muscle
RYR2
MYH11
ACTA2
TAGLN
UMAPharmony_1
UMAPharmony_2

Supplementary figure 13. UMAP plots demonstrating expression of selected marker genes used for cell type annotation. Major cell types identified for the brain (top), heart (middle), and Lung (bottom) are shown with the expression level (scTransform) of four selected markers.

Supplementary methods Tables and Figures

Supplementary method table 1. Information for the 12 individuals with both a cord blood sample and a later life sample available.

Anonymous identifier	Cohort	Sex	SGA status	Gestational age	SGA endotype
X258633890922_7	IIS	Female	SGA	39	nonAG-SGA
X258633890921_7	IIS	Male	SGA	39	AG-SGA
X258633890933_5	IIS	Male	SGA	40	nonAG-SGA
X258633851991_1	TCRS	Female	SGA	36	AG-SGA
X258633851962_1	TCRS	Male	SGA	39	nonAG-SGA
X258633851991_2	TCRS	Female	SGA	40	nonAG-SGA
X258633851963_3	TCRS	Female	SGA	39.5	nonAG-SGA
X258633851958_3	TCRS	Female	SGA	38.3	nonAG-SGA
X258633851964_5	TCRS	Female	SGA	39	nonAG-SGA
X258633851962_6	TCRS	Female	SGA	39	AG-SGA
X258633851990_2	TCRS	Male	AGA	40.3	AGA
X258633890936_7	IIS	Female	SGA	39	nonAG-SGA

Abbreviations: SGA, Small for Gestational Age; IIS, Infant Immune Study; TCRS, Tucson Children's Respiratory Study; AG, Axon guidance.

Supplementary method table 2. The α value used in the elastic net regression models.

	FEV1	FVC	%FEV1/FVC
All aptamers	0.4	0.1	1
Restricted aptamers	0.1	0.1	1

Abbreviations: FEV1, Forced Expiratory Volume in 1 Second; FVC, Forced Vital Capacity; %FEV1/FVC, Forced Expiratory Volume in 1 Second percentage of Forced Vital Capacity.

Supplementary method table 3. Key QC metrics for each sheep scRNA-Seq profile.

Sample ID	Sheep	Organ	Condition	No. cells	Median genes per cell	Median transcripts per cell	Total transcripts	Median mitochondrial content (%)
**AB14**	A	Brain	CTRL	3,779	684	2,081	9,627,981	0
**AB15**	B	Brain	CTRL	2,479	1,737	4,002	12,796,056	0
**AB16**	A	Lung	CTRL	2,386	2,071	5,524.5	15,260,384	0
**AB17**	B	Lung	CTRL	2,546	2034	5,265	15,247,270	0
**AB18**	A	Heart	CTRL	2,316	1,529.5	3,031.5	8,496,114	0
**AB19**	B	Heart	CTRL	2,811	1,264	2,286	7,778,547	0
**AB22**	C	Brain	FGR	14,127	822	2,896	58,308,287	0
**AB23**	D	Brain	FGR	1,281	3,747	12,458	18,277,085	0
**AB24**	C	Lung	FGR	6,270	1,995	5,159.5	38,138,043	0
**AB25**	D	Lung	FGR	7,709	1,750	4,210	39,835,829	0
**AB26**	C	Heart	FGR	996	412.5	1,686	1,916,617	0
**AB27**	D	Heart	FGR	720	2,198.5	5,818.5	4,670,281	0
				Total cells	Average median genes	Average median transcript	Average total transcripts	Average mitochondrial content
				**47,420**	**1,687.04**	**4,535**	**19,196,041**	**0.0**

Abbreviations: CTRL, Control; FGR, Fetal Growth Restriction.

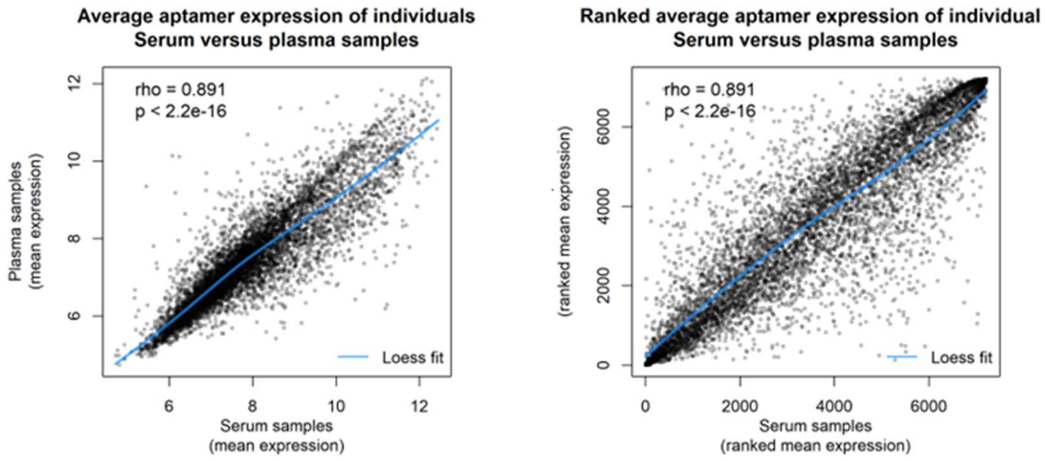

Supplementary method figure 1. Plots showing the correlation of proteomic data generated from serum (x-axis) and plasma (y-axis) samples. Plots show the average aptamer expression (RFU) (left) and ranked average expression (right). The blue line represents a Loess fit of the data, and the text show the Spearman's rho and associated p values of the correlation.

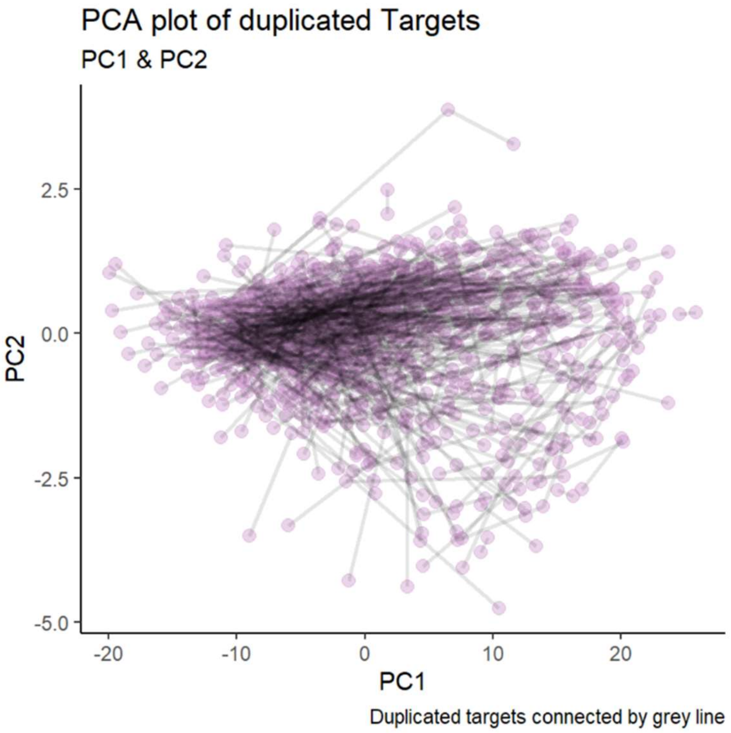

Supplementary method figure 2. Example PC plot with links between aptamers corresponding to the same target protein. This demonstrates little overlap in the space occupied by linked aptamers.

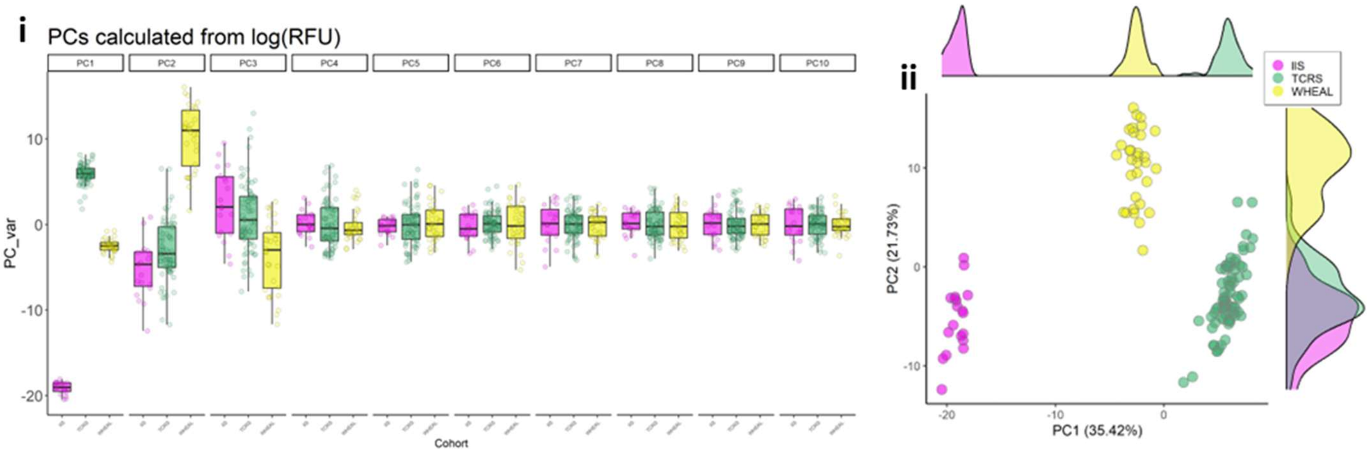

Supplementary methods figure 3. Principal component analysis of the log(RFU) values of all aptamers for the proteomic profiles generated from later life blood samples. (**i**) Boxplots of the top 10 PCs, stratified by cohort of origin. (**ii**) Scatterplot of the first two PCs colored by cohort.

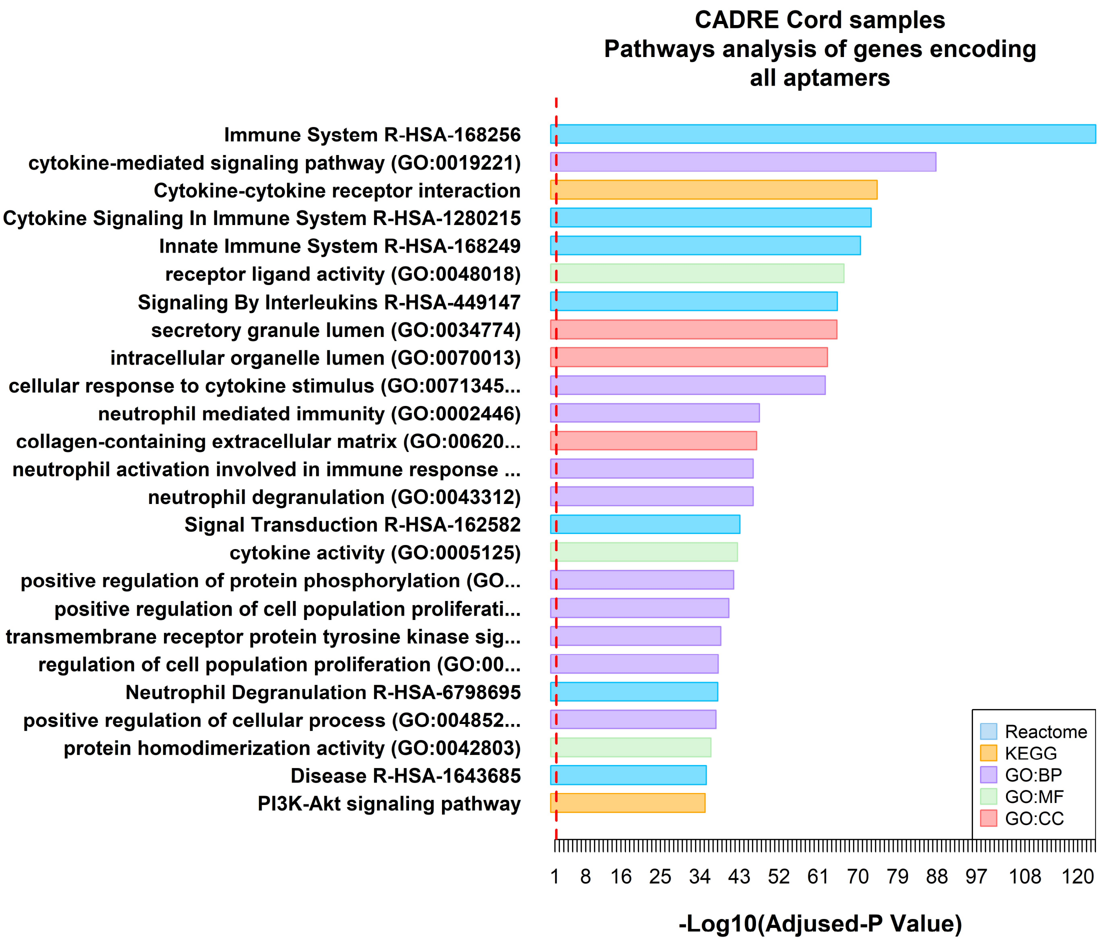

Supplementary method figure 4. Horizontal bar plot displays the top 25 enriched of genes that encode all aptamers used in this study (n = 7,214). The x-axis shows the adjusted enrichment p value and the y-axis show the pathway terms. Bars are colored according to the repository of the enriched term.

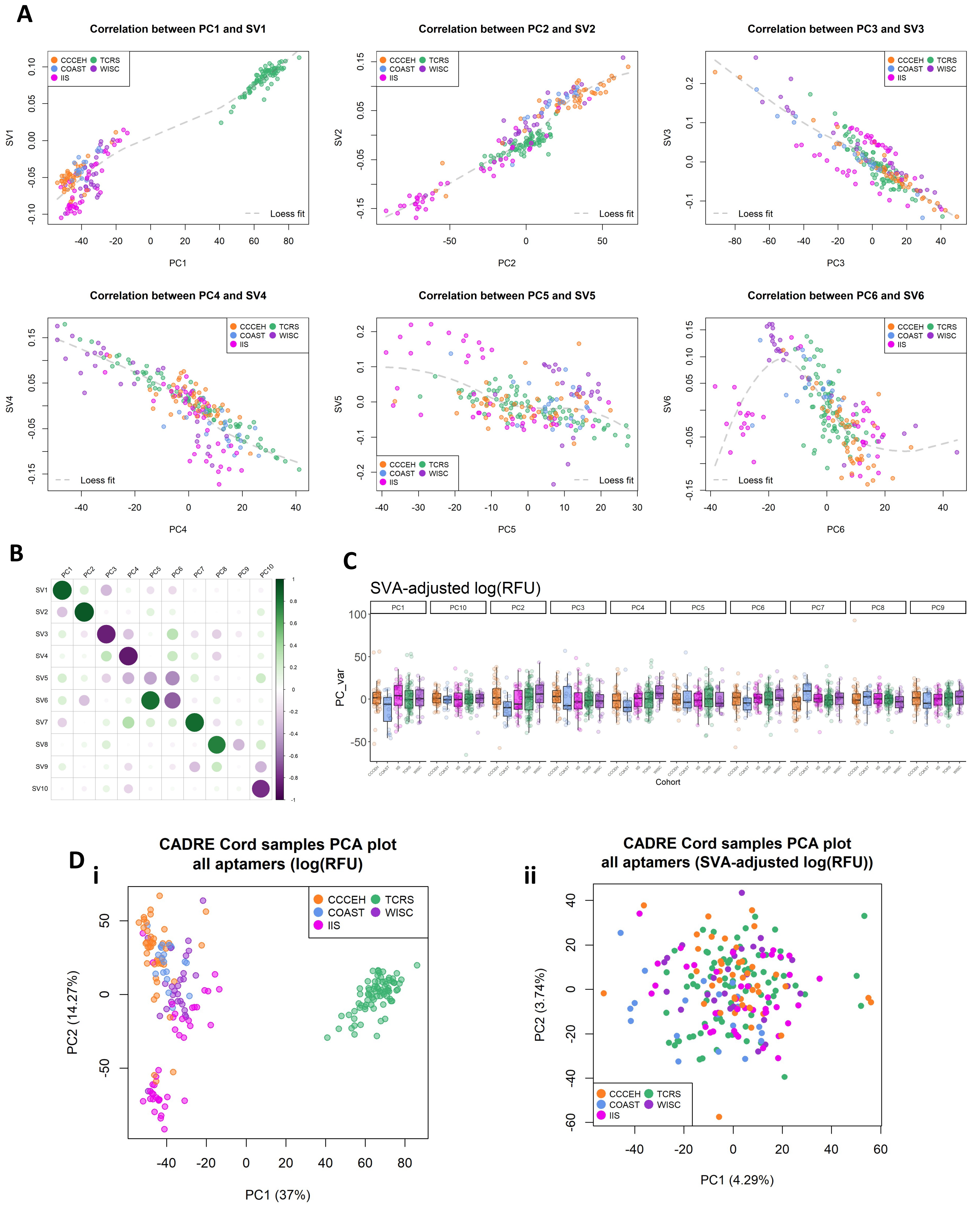

Supplementary methods figure 5. **A**) Correlation plots between principal components (x-axis) and surrogate variables (y-axis) for the top 6 PCs/SVs. Each point represents a cord blood-derived proteomic profiles and is colored by the cohort of origin. The dashed grey line represents a lowess fit of the data. **B**) Correlation dot plot showing the top 10 PCs (x-axis) and top 10 SVs (y-axis). The color and size correspond to the strength of the positive or negative correlation. **C**) Boxplots of the top 10 PC of the log(RFU) values after adjustment for SVs, stratified by cohort of origin. **D**) Comparison of scatterplots of the first two PC calculated on the log(RFU) data without (**i**) and with (**ii**) adjustment for SVs. Points are colored by the cohort of origin of the cord blood samples.

A

CCCEH

	R2X	R2X(cum)	R2Y	R2Y(cum)	Q2	Q2(cum)	Signif.	Iter.
p1	0.0552	0.0552	0.8390	0.839	0.519	0.519	R1	1
p2	0.0475	0.1030	0.1340	0.973	0.310	0.668	R1	1
p3	0.0412	0.1440	0.0227	0.996	0.258	0.754	R1	1

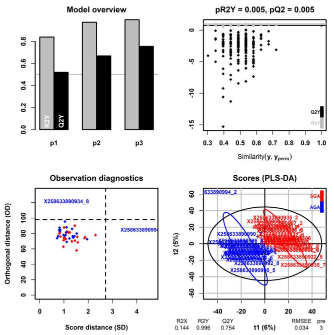

COAST

	R2X	R2X(cum)	R2Y	R2Y(cum)	Q2	Q2(cum)	Signif.	Iter.
p1	0.1540	0.154	0.916	0.916	0.628	0.628	R1	1
p2	0.0985	0.253	0.075	0.991	0.323	0.748	R1	1

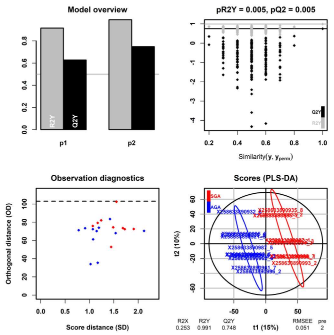

IIS

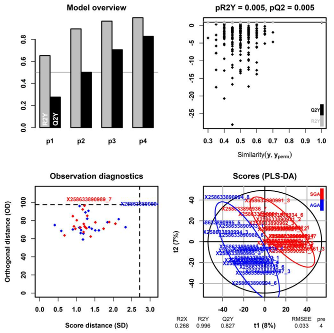

TCRS

	R2X	R2X(cum)	R2Y	R2Y(cum)	Q2	Q2(cum)	Signif.	Iter.
p1	0.0526	0.0526	0.6550	0.655	0.355	0.355	R1	1
p2	0.0297	0.0823	0.2760	0.931	0.379	0.600	R1	1
p3	0.0316	0.1140	0.0507	0.982	0.265	0.706	R1	1
p4	0.0379	0.1520	0.0118	0.994	0.252	0.780	R1	1

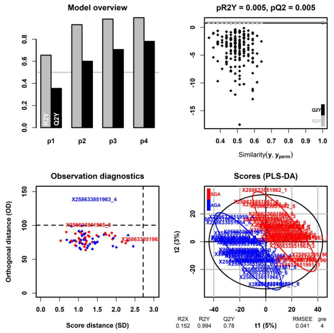

WISC

	R2X	R2X(cum)	R2Y	R2Y(cum)	Q2	Q2(cum)	Signif.	Iter.
p1	0.1140	0.114	0.667	0.667	0.282	0.282	R1	1
p2	0.0743	0.188	0.276	0.943	0.411	0.577	R1	1

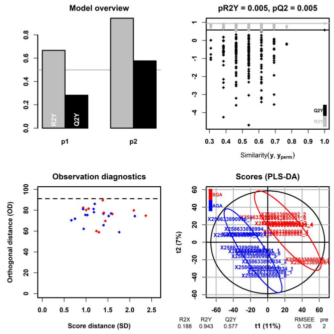

B

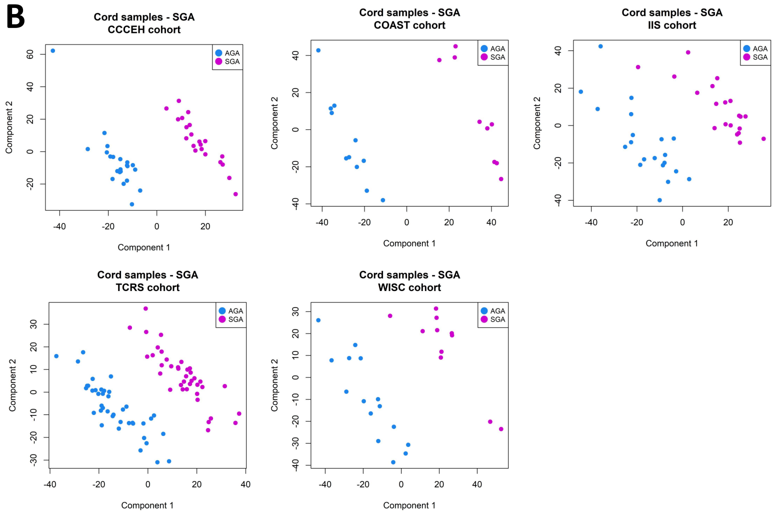

C

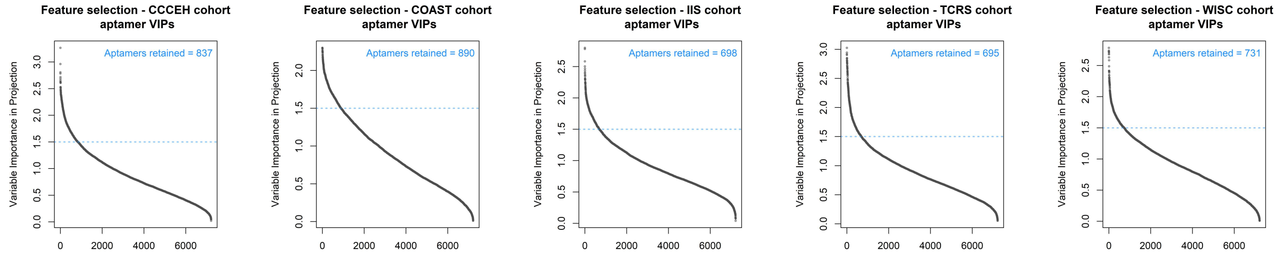

Supplementary method figure 6 (previous page). Output plots from PLS feature selection. **A**) Tabulated key metrics and output plots generated from the *opls* function from the rpols package used for PLS analysis, stratified by cohort. **B**) Plot of the first two components from the PLS analysis of each cohort demonstrating the successful stratification of sample by SGA and AGA status. **C**) Plot showing the number of aptamers selected for each cohort via a VIP threshold of 1.5. The x-axis shows the ranked aptamers, and the y-axis shows the VIP metric. The dashed blue line represents the VIP threshold above which aptamers were selected. The number of selected aptamers is displayed in blue text.

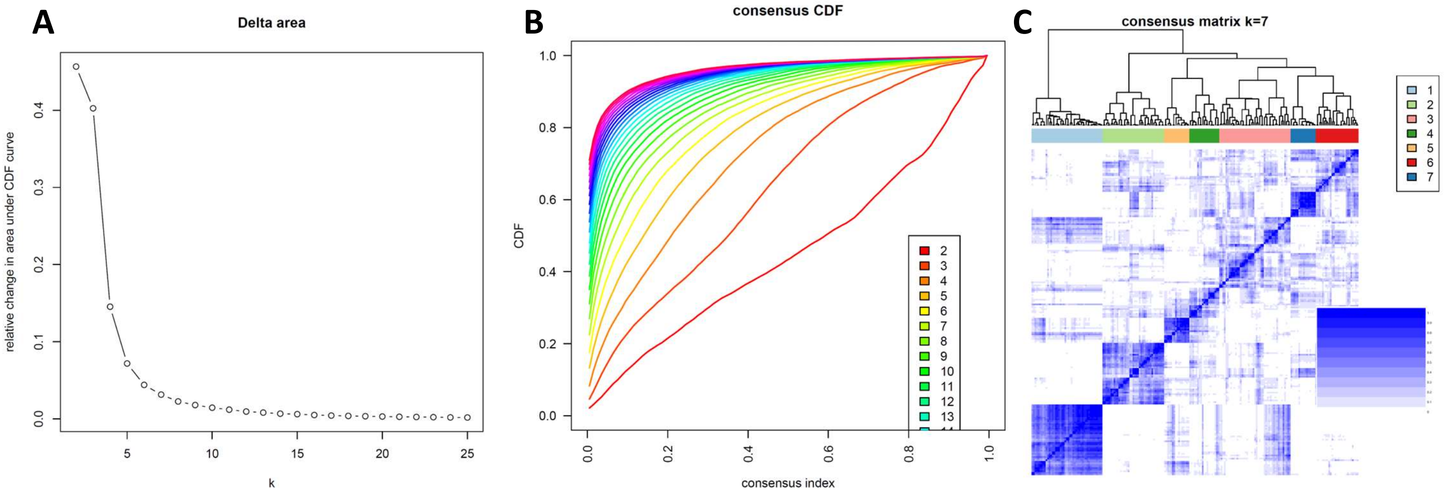

Supplementary method figure 7. Output plots from consensus clustering optimization. **A**) Plot showing the Cumulative Distribution Functions (CDF) the consensus matrix for each k (number of clusters). **B**) Plot showing the relative change in area under the CDF curve for each k compared to the previous k (k - 1), which visualizes the cluster number after which there is little appreciable increase in information. Optimal values of k from the plots in A and B are those which capture the majority of the information (between cluster variation), without creating arbitrary small clusters which add little or no additional information (i.e., where the plots converge/level off). **C**) Heatmaps of the consensus matrix of the selected cluster number (k = 7) displaying the occurrence of samples clustering together during resampling, which demonstrates the consensus clustering stability across 1,000 resamples. The gradient of dark blue to white, shown in the top legend, represents the proportion that each sample co-clustered with all other samples from 1,000 resamples. For any two sample pairs, the deepest blue color indicates that the two samples were always assigned the same cluster and white indicates that they were never assigned the same cluster. The dendrogram and corresponding color bar represent the clustering at k = 7, with cluster number shown in the bottom legend.

A

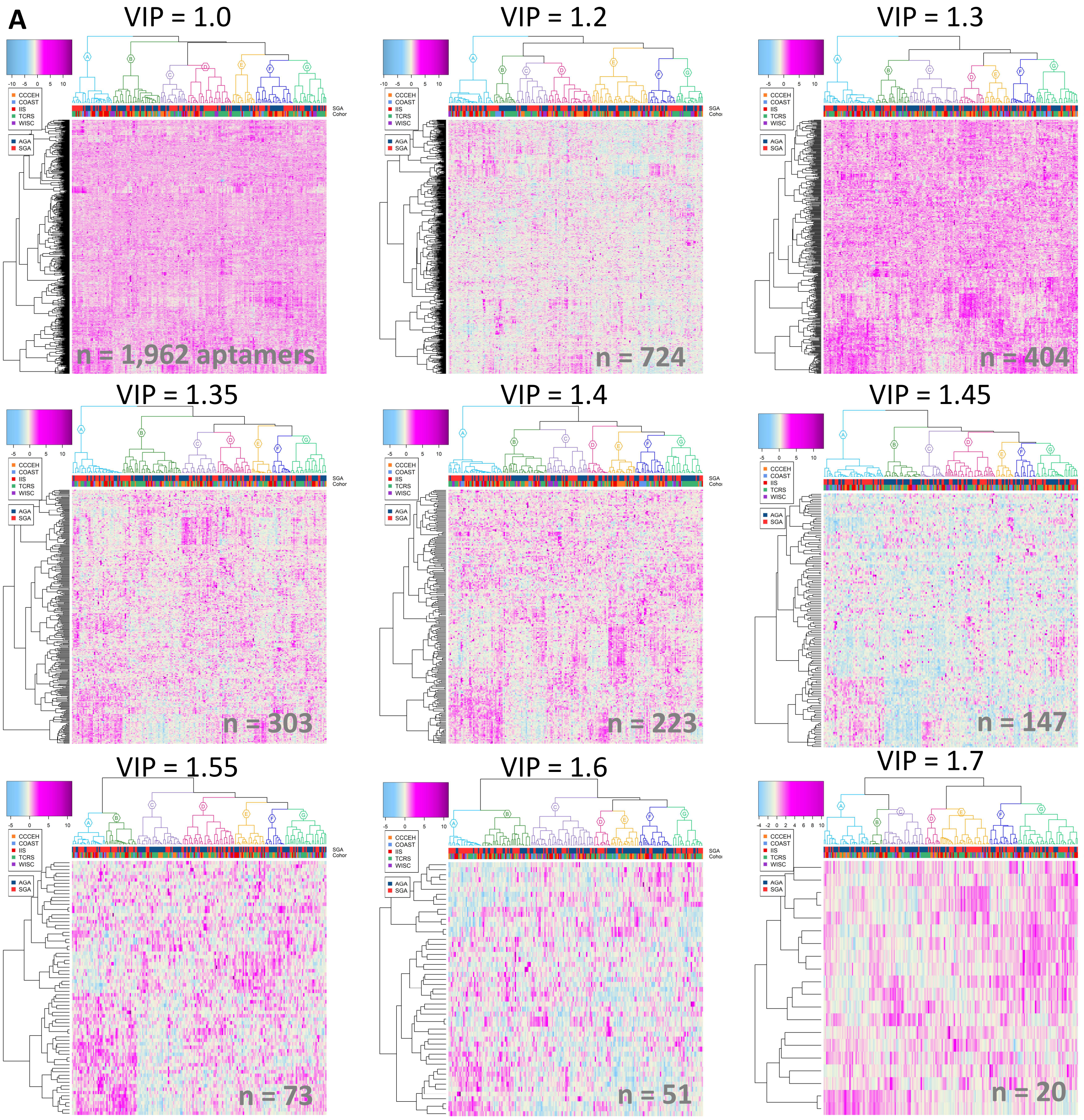

B

1.0

	AGA	SGA	% SGA
A	12	21	63.64%
B	28	11	28.21%
C	9	13	59.09%
D	14	22	61.11%
E	16	3	15.79%
F	14	17	54.84%
G	15	12	44.44%

1.2

	AGA	SGA	% SGA
A	13	24	64.86%
B	14	4	22.22%
C	12	14	53.85%
D	17	19	52.78%
E	33	12	26.67%
F	8	15	65.22%
G	11	11	50%

1.3

	AGA	SGA	% SGA
A	11	31	73.81%
B	16	12	42.86%
C	31	9	22.5%
D	12	9	42.86%
E	8	13	61.9%
F	9	12	57.14%
G	21	13	38.24%

1.35

	AGA	SGA	% SGA
A	11	30	73.17%
B	31	17	35.42%
C	15	14	48.28%
D	13	15	53.57%
E	10	7	41.18%
F	8	7	46.67%
G	20	9	31.03%

1.4

	AGA	SGA	% SGA
A	11	33	75%
B	25	11	30.56%
C	19	14	42.42%
D	10	7	41.18%
E	13	9	40.91%
F	12	12	50%
G	18	13	41.94%

1.45

	AGA	SGA	% SGA
A	13	36	73.47%
B	26	4	13.33%
C	11	9	45%
D	20	21	51.22%
E	8	7	46.67%
F	11	7	38.89%
G	19	15	44.12%

1.55

	AGA	SGA	% SGA
A	7	20	74.07%
B	7	19	73.08%
C	28	9	24.32%
D	24	18	42.86%
E	12	13	52%
F	10	6	37.5%
G	20	14	41.18%

1.6

	AGA	SGA	% SGA
A	4	23	85.19%
B	18	22	55%
C	33	18	35.29%
D	6	8	57.14%
E	20	3	13.04%
F	20	7	25.93%
G	7	18	72%

1.7

	AGA	SGA	% SGA
A	25	12	32.43%
B	9	2	18.18%
C	24	10	29.41%
D	7	6	46.15%
E	13	27	67.5%
F	9	14	60.87%
G	21	28	57.14%

Supplementary method figure 8. Consensus clustering results with alternative VIP values. **A**) Heatmaps demonstrating the consensus clustering outcomes with different VIP values (heatmap parameters are the same as those in Figure 1A in the main text). Grey text indicates the number of aptamers (identified in ≥ 3 cohorts) for that VIP threshold. **B**) Tables showing the number of AGA and SGA subjects in each cluster, and the percentage of SGA subjects, for each VIP threshold.

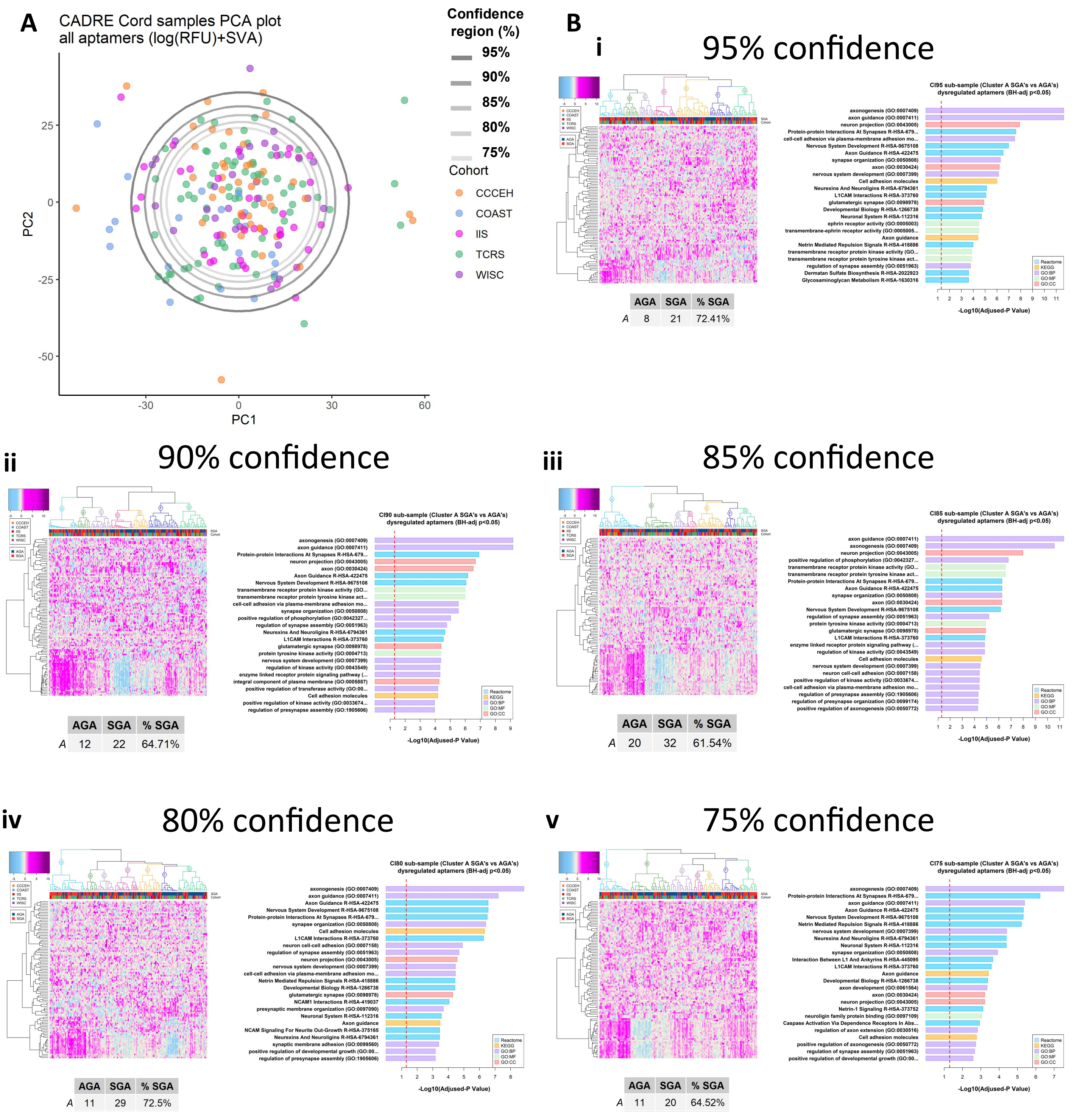

Supplementary method figure 9. Sensitivity analysis of more homogenous sample subsets. **A**) Plot of the first (x-axis) and second (y-axis) principal components with grey ellipses illustrating confidence regions of 95%, 90%, 85%, 80%, and 75%. **B**) Consensus cluster heatmap, tabulated number/percentage of SGA samples, and vertical bar plot of significantly enriched pathways from genes encoding dysregulated aptamers between Cluster A SGAs and all AGA (limma analysis) for each confidence region.

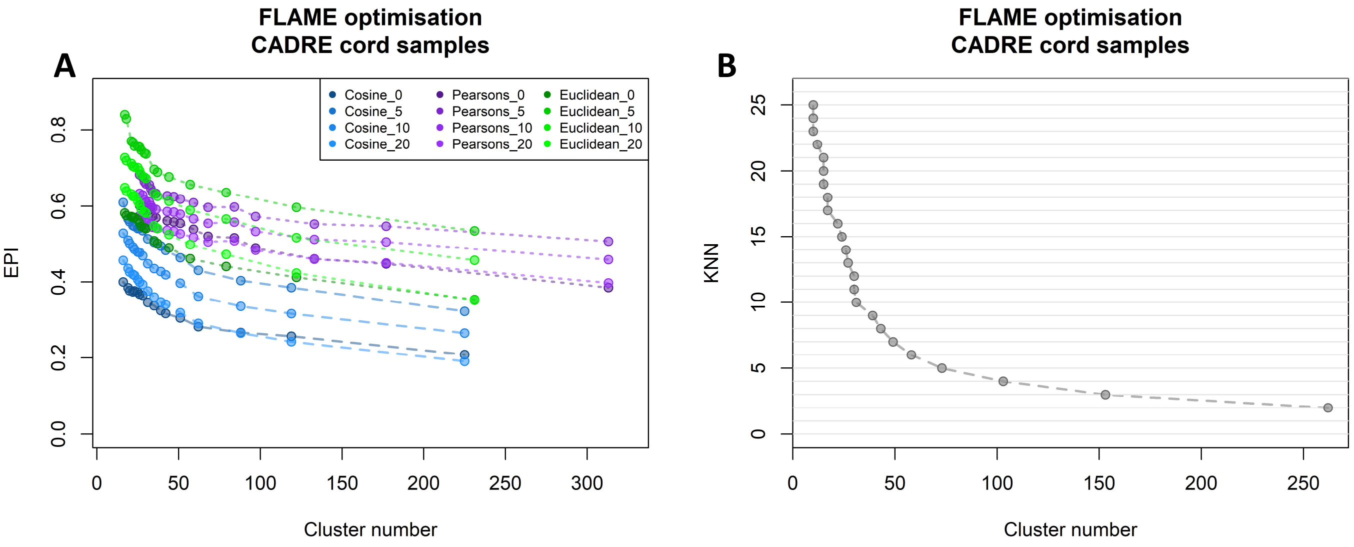

Supplementary method figure 10. Output plots from FLAME optimization. **A**) Plot of the number of clusters (x-axis) versus the optimization partition index (EPI, y-axis). Each line represents a correlation/distance metric used for network construction and FLAME threshold (e.g., Cosine_0 represents the network constructed from a cosine matrix with a 0% threshold). Lines are colored according to the correlation/distance metric, and lower values of the partition index indicate better performance. **B**) Elbow plot showing the relationship between the number of clusters (x-axis) and KNN (y-axis). Optimal values of KNN are where the plot begins to bend and level out.

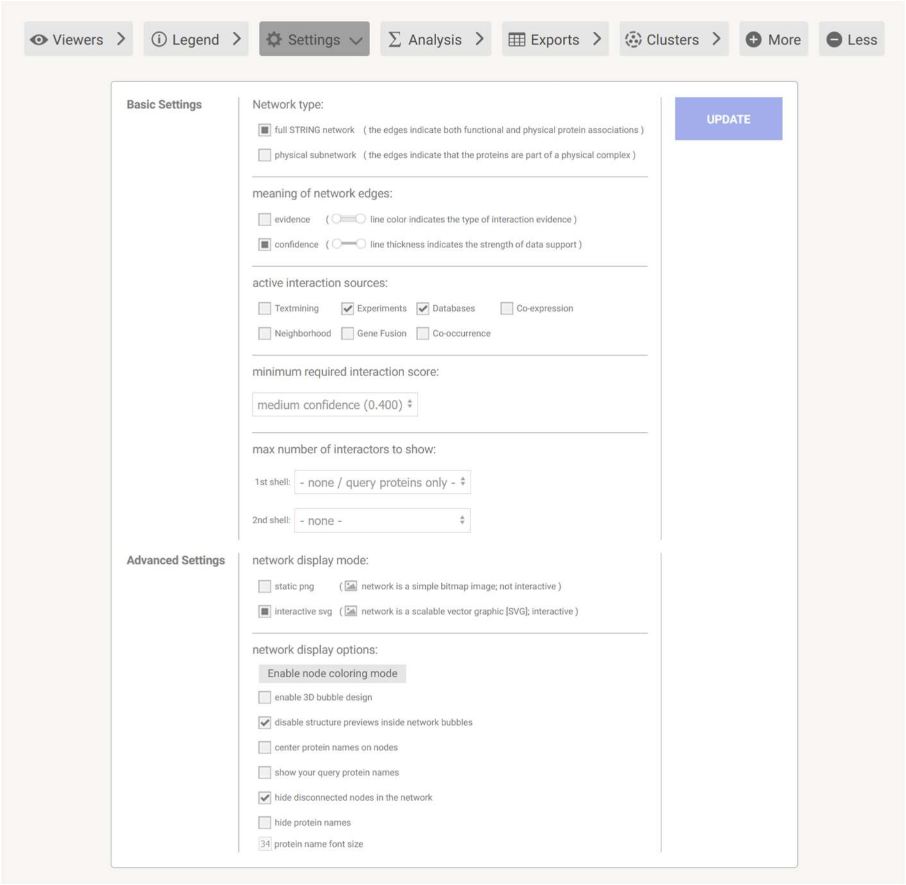

Supplementary method figure 11. Screenshot of the input setting parameters used to construct PPI network with STRING (Version 12.0).

All available aptamer models

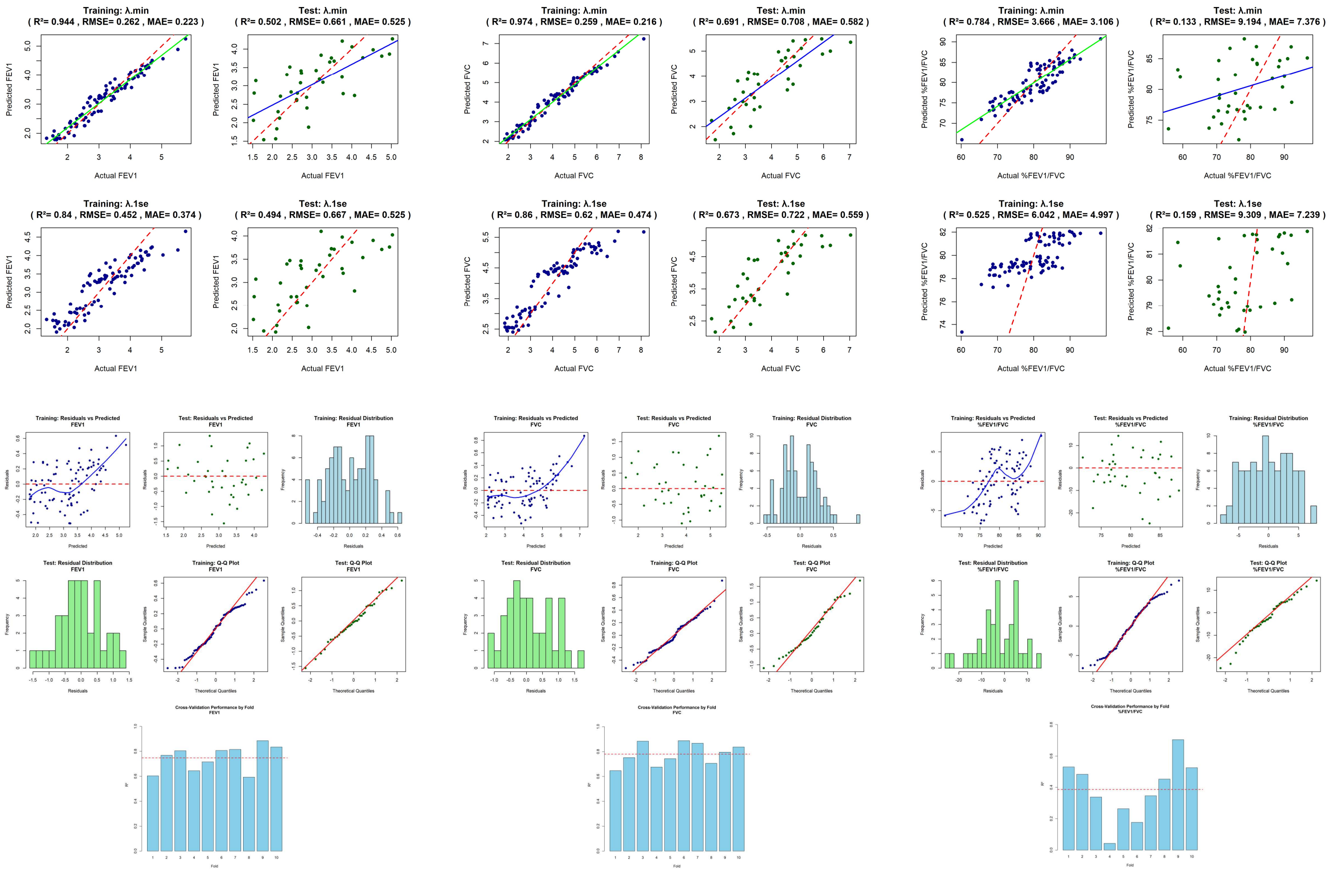

Restricted aptamer models

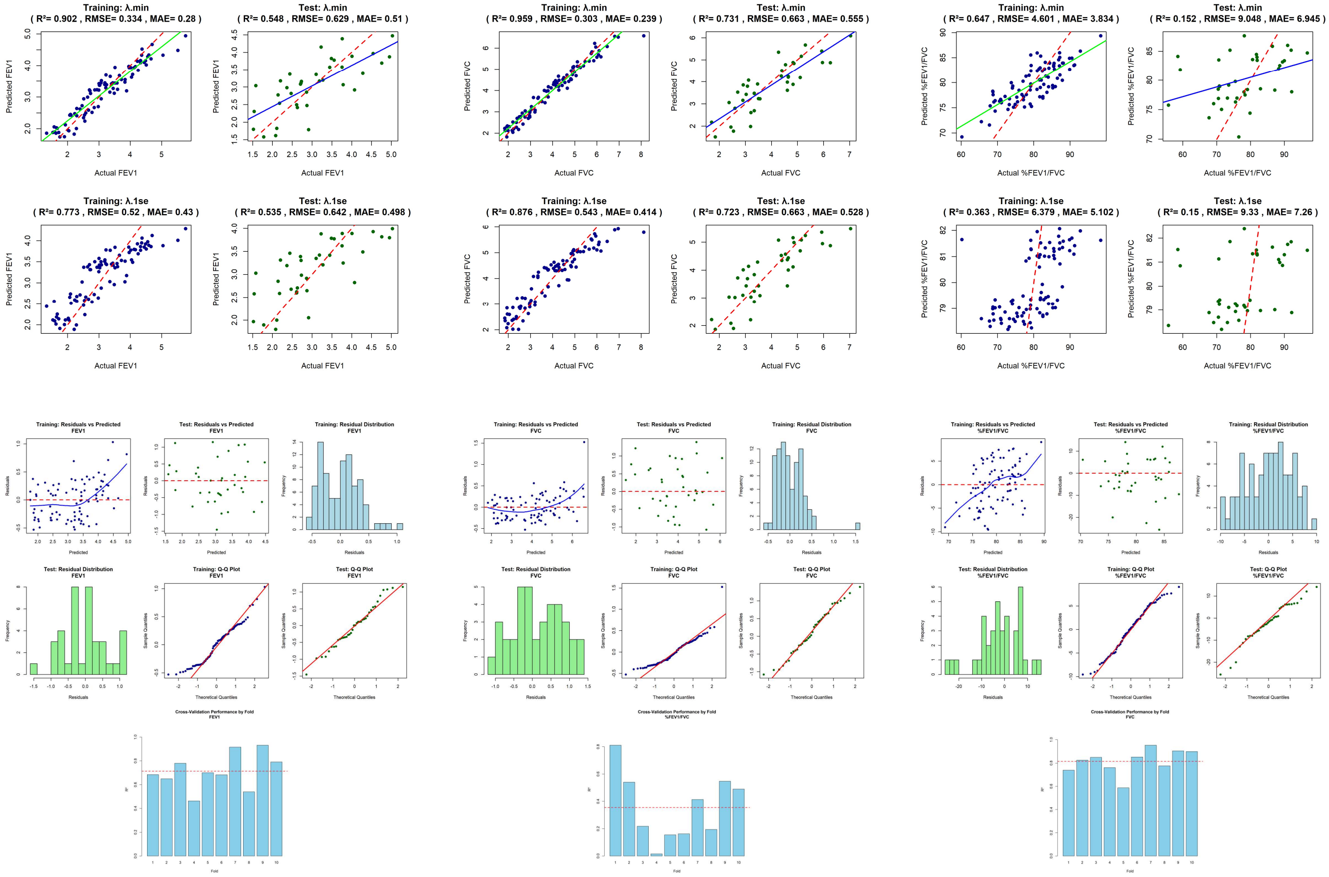

Supplementary method figure 12 (previous page). Elastic net model output plots for models with all aptamers (top) and restricted aptamers (n=221, bottom), arranged to show plots for FEV_1 (left), FVC (middle), and $\%FEV_1/FVC$ (right). In each case, the first four plots show the prediction of the lung function measurement from the training and test sets using the 'minimum' and '1se' λ values, the middle six plots show common analysis plots of model residuals, and the lowest plot shows the R^2 values for each cross-validation fold.
