# Supplementary material for: Multicohort analysis unveils axon guidance pathways linking small for gestational age to spirometric restriction
Source: Nat Commun. 2026 May 2;17:5952. doi: 10.1038/s41467-026-72490-w (PMC13342533; doi:10.1038/s41467-026-72490-w)
Supplement: Supplementary file 2 — Reporting Summary [file 41467_2026_72490_MOESM2_ESM.pdf]

nature portfolio

Corresponding author(s): James F Read

Last updated by author(s): Mar 3, 2026

Reporting Summary

Nature Portfolio wishes to improve the reproducibility of the work that we publish. This form provides structure for consistency and transparency in reporting. For further information on Nature Portfolio policies, see our Editorial Policies and the Editorial Policy Checklist.

Statistics

For all statistical analyses, confirm that the following items are present in the figure legend, table legend, main text, or Methods section.

n/a	Confirmed	
[ ]	[x]	The exact sample size (*n*) for each experimental group/condition, given as a discrete number and unit of measurement
[ ]	[x]	A statement on whether measurements were taken from distinct samples or whether the same sample was measured repeatedly
[ ]	[x]	The statistical test(s) used AND whether they are one- or two-sided<br>*Only common tests should be described solely by name; describe more complex techniques in the Methods section.*
[ ]	[x]	A description of all covariates tested
[ ]	[x]	A description of any assumptions or corrections, such as tests of normality and adjustment for multiple comparisons
[ ]	[x]	A full description of the statistical parameters including central tendency (e.g. means) or other basic estimates (e.g. regression coefficient) AND variation (e.g. standard deviation) or associated estimates of uncertainty (e.g. confidence intervals)
[ ]	[x]	For null hypothesis testing, the test statistic (e.g. *F*, *t*, *r*) with confidence intervals, effect sizes, degrees of freedom and *P* value noted<br>*Give P values as exact values whenever suitable.*
[x]	[ ]	For Bayesian analysis, information on the choice of priors and Markov chain Monte Carlo settings
[ ]	[x]	For hierarchical and complex designs, identification of the appropriate level for tests and full reporting of outcomes
[ ]	[ ]	Estimates of effect sizes (e.g. Cohen's *d*, Pearson's *r*), indicating how they were calculated

Our web collection on statistics for biologists contains articles on many of the points above.

Software and code

Policy information about availability of computer code

Data collection	The cohorts used in this study are part of the CADRE (formerly CREW) consortium of birth cohorts that investigate early life risk factors for respiratory disease, particularly asthma. Data were collected by the original cohorts using a variety of methods, including enterprise and proprietary software systems as well as paper-based forms. Data collection for CREW involved the creation of the CREW Asthma eLab database, a version of the Asthma eLab initially developed by investigators at the University of Manchester for the STELAR birth cohort consortium. This serves as a serves as a secure database, a forum for investigator interaction, and a platform for analyses and manuscript development. Refer to the following publication:<br>Gern J et al. The Children's Respiratory and Environmental Workgroup (CREW) birth cohort consortium: design, methods, and study population. Respir Res. 2019 Jun 10;20(1):115<br><br>Data collected directly by the CADRE Consortium was collected and managed using REDCap electronic data capture tools hosted at the University of Wisconsin-Madison. Data sets were produced using IBM Netezza SQL (v11.2) and PostgreSQL (PostgreSQL 14.20 on x86_64_pc_linux-gnu, compiled by gcc (GCC) 13.2.0, 64-bit) as well as Python (v3.10.8).
Data analysis	No custom code was generated for this study, and all software/packages used were open source.<br>All data QC and analysis was conducted in the R (version 4.3.2) environment. Linear Models for Microarray Analysis (limma) was used for differential protein analysis. The Enrichr database (accessed via the EnrichR R package) was used for pathways enrichment analysis. The sva R package was used for surrogate variable analysis. The FactoMineR package was used for PCA. Partial Least Squares (PLS) classification was used for feature selection via the opls R package. Sparse Partial Least Squares – Discriminatory Analysis (sPLS-DA) was applied with the mixOmics R package. The ConsensusClusterPlus R package was used for consensus clustering. Fuzzy clustering by Local Approximation of MEmbership (FLAME) was employed for module detection. STRING (Version 12.0) software was used to construct PPI networks. The glmnet R package was used for elastic net models. The NHGRI-EBI Catalog of human Genome-Wide Association Studies was used to identify gene

variants linked to pulmonary traits from previously published GWAS studies. The Seurat (version 5.0.3) R package was primarily used for single cell transcriptomics analysis. Code used for analyses is available on Dryad (https://doi.org/10.5061/dryad.5mkkwh7h3) [upon publication].

For manuscripts utilizing custom algorithms or software that are central to the research but not yet described in published literature, software must be made available to editors and reviewers. We strongly encourage code deposition in a community repository (e.g. GitHub). See the Nature Portfolio guidelines for submitting code & software for further information.

Data

Policy information about availability of data

All manuscripts must include a data availability statement. This statement should provide the following information, where applicable:

- Accession codes, unique identifiers, or web links for publicly available datasets
- A description of any restrictions on data availability
- For clinical datasets or third party data, please ensure that the statement adheres to our policy

Proteomic data used for analysis is available on Github (github.com/jamesfread/CADRE_manuscript) and scRNA-Seq data generated from the sheep model is available on Dryad (https://doi.org/10.5061/dryad.5mkkwh7h3). There are no restrictions on data availability. Proteomic data was generated with the SomaScan platform from SomaLogic (Boulder, CO, USA). scRNA-Seq data was generated with the 10x Genomics (Pleasanton, CA, USA) Universal 3' Gene Expression workflow. GWAS data was queried from the NHGRI-EBI Catalog of human Genome-Wide Association Studies.

Research involving human participants, their data, or biological material

Policy information about studies with human participants or human data. See also policy information about sex, gender (identity/presentation), and sexual orientation and race, ethnicity and racism.

Reporting on sex and gender	The term sex (biological attribute) was used in this study, and gender (shapes by social and cultural circumstances) was not used. Sex was defined as the sex assigned at birth. 106/207 (51.21%) of participants providing cord blood samples and 66/126 (52.38%) of participants providing later life blood samples were assigned female at birth in this study, representing an approximately even split of male/female subjects.<br>Assessment of the data with principal component analysis demonstrated that minimal variation in the data could be attributed to sex in this study. Regardless, sex-based analyses were performed and reported throughout this manuscript, including demographic analysis/tables, principal component analysis, sparse Partial Least Squares discriminatory analysis (multivariate classification), enrichment analysis (Fisher's test) among experimental (sub)groups, and adjusted as a covariate in multivariate models.
Reporting on race, ethnicity, or other socially relevant groupings	This study used included parental self-reported ethnicity, and these included Caucasia/White, African American, Hispanic, Asian, or Other. The cohorts used in this study were collected from geographically diverse United States populations, and as such each cohort comprised a different ethnicity profile corresponding to the recruitment area. For example, The WISC cohort was recruited in rural Wisconsin and included ~95% Caucasian/white participants, whereas the WHEALS cohort was recruited in metropolitan Detroit, Michigan and included ~63% African American participants. The decision to generate the data from these different populations was made to ensure generalizability of the findings. Confounding variables were controlled for by using surrogate variables or the term (e.g., 'cohort') as a covariate, as appropriate for the analysis. Surrogate variate analysis identifies latent sources of unwanted variation present in the data that are unrelated to variables of interest (e.g., SGA status), which can be adjusted for while retaining the variation of interest.
Population characteristics	This study assessed differences between individuals born Small for Gestational Age (SGA) and those born Average for Gestational Age (AGA). The data generated from cord blood samples collected at birth (n = 207) came from 99 SGA births (47.83%) and 108 AGA births (52.17%). The data generated from samples collected in later life (n = 126) included 46 (36.51%) SGA and 80 (63.49%) AGA births. The later life samples were collected from participants at either 8 years (19/126 (15.08%)), 11-13 years (32/126 (25.4%)), and 36 years (75/126 (59.52%), from the IIS, WHEALS, and TCRS cohorts, respectively.
Recruitment	This study employed prospective birth cohort that were established to investigate early life risk factors for respiratory disease, particularly asthma. Expecting parents were recruited to take part in the respective studies, and relvent information was collected from their child from birth. Written informed consent or parent/guardian permission was obtained along with child assent as appropriate for participation in specific cohorts and the CREW protocol
Ethics oversight	The local institutional review board at each participating site approved the study protocol. Written informed consent or parent/guardian permission was obtained along with child assent as appropriate for participation in specific cohorts and the CREW protocol. All participating institutions approved the data sharing procedures specified in the CREW and/or CADRE consortium agreements. Animal protocols were approved by the Institutional Animal Care and Use Committee at the University of Arizona. All experiment followed the guidelines set by the US National Research Council's "Guide for The Care and Use of Laboratory Animals" and the US Public Health Service's "Policy on Humane Care and Use of Laboratory Animals."

Note that full information on the approval of the study protocol must also be provided in the manuscript.

Field-specific reporting

Please select the one below that is the best fit for your research. If you are not sure, read the appropriate sections before making your selection.

- [x] Life sciences
- [ ] Behavioural & social sciences
- [ ] Ecological, evolutionary & environmental sciences

For a reference copy of the document with all sections, see nature.com/documents/nr-reporting-summary-flat.pdf

Life sciences study design

All studies must disclose on these points even when the disclosure is negative.

Sample size	Subjects from the CADRE cohorts with available cord blood (n=207) or later life peripheral blood (n=126) samples were selected as SGA with birthweight lower than the 10th percentile or AGA with birthweight between the 10th and 90th percentile. All individuals assessed in this study (SGA and AGA) were born at term (i.e., no participants had preterm births). The cohorts employed in this study were each collected for a specific purpose and have facilitated many independent investigations for decades, many of which used blood sample aliquots. Therefore, the subjects available for this study were those with remaining sample aliquots. For this reason, sample size calculations were not conducted prior to data generation. However, the core data for this study was generated with the SomaScan proteomics platform, that measures >7,000 proteins, resulting in a large, deeply profiled dataset.
Data exclusions	No Individuals were explicitly excluded from the study based on exclusion criteria. As mentioned above, the subjects available for this study were those with remaining sample aliquots for several, well-characterized birth cohorts. The final samples were selected to maximize sample number whilst maintaining an approximately even split of SGA/AGA and female/male subjects for each cohort. Following proteomic data generation, the later life (childhood) data for the COAST and WISC cohorts were not used in analysis, due to not having spirometry data recorded (COAST) and few samples (n=3, WISC).
Replication	The main experimental finding from this study was the link between SGA status and greater abundance of axon guidance (AG) related proteins in cord blood samples. The study presents an integrated analysis of all cohorts, however this finding was also observed in each cohort independently, demonstrating replication across geographically-diverse US populations. While the AG/SGA association was replicated in each cohort (n=5), the strength of the association varied. For example, AG proteins were the most dominant feature associated with SGA in the TCRS and COAST cohorts, whereas AG proteins were present, but immune-related features were more prominent in the CCCEH and IIS cohorts. Further replication of this finding in independent, globally-diverse populations has not yet been conducted, although this is a key future direction for this work.
Randomization	The samples used in this study were curated from longitudinal birth cohorts, and the groups analyzed were based on charateristics determined at birth (i.e., SGA status) and lung function test metrics in later life. As such, the participants were not randomly assigned groups. For data generation, the sample order was randomized and checked for even distribution with respect to SGA status and sex to ensure there was no inadvertent grouping by SGA/sex in the sequence order.
Blinding	Blinding was not relevant to this study design as it employed several observational birth cohorts and the experimental groups were assigned based on observed birth characteristics/clinical measurements.

Reporting for specific materials, systems and methods

We require information from authors about some types of materials, experimental systems and methods used in many studies. Here, indicate whether each material, system or method listed is relevant to your study. If you are not sure if a list item applies to your research, read the appropriate section before selecting a response.

Materials & experimental systems

n/a	Involved in the study	
☒	☐	Antibodies
☒	☐	Eukaryotic cell lines
☒	☐	Palaeontology and archaeology
☐	☒	Animals and other organisms
☒	☐	Clinical data
☒	☐	Dual use research of concern
☒	☐	Plants

Methods

n/a	Involved in the study	
☒	☐	ChIP-seq
☒	☐	Flow cytometry
☒	☐	MRI-based neuroimaging

Animals and other research organisms

Policy information about studies involving animals; ARRIVE guidelines recommended for reporting animal research, and Sex and Gender in Research

Laboratory animals	Ovis aries (sheep), Columbia-Rambouillet breed, were used in this study. Columbia-Rambouillet crossbred ewes with singleton pregnancies were purchased from the University of Arizona Sheep Unit. The ewes were two to four years of age with unknown parity. Singleton fetuses were determined by ultrasonography prior to randomized group assignment.
Wild animals	No wild animals were used in this study.
Reporting on sex	Both male and female fetal lambs were studied. However, due to the paucity of animal numbers, the statistical model was not powered to evaluate sex differences. Therefore, sex was excluded from the analysis. Fetal sex was assigned at necropsy by visual inspection of external genitalia.
Field-collected samples	No field-collected samples were used in this study.

Ethics oversight	Animal protocols were approved by the Institutional Animal Care and Use Committee at the University of Arizona. All experiment followed the guidelines set by the US National Research Council's "Guide for The Care and Use of Laboratory Animals" and the US Public Health Service's "Policy on Humane Care and Use of Laboratory Animals". The reporting of this model abides by the ARRIVE guidelines for reporting of in vivo work. Oversight was provided by the University of Arizona IRB for this sheep model.

Note that full information on the approval of the study protocol must also be provided in the manuscript.

Plants

Seed stocks	*Report on the source of all seed stocks or other plant material used. If applicable, state the seed stock centre and catalogue number. If plant specimens were collected from the field, describe the collection location, date and sampling procedures.*
Novel plant genotypes	*Describe the methods by which all novel plant genotypes were produced. This includes those generated by transgenic approaches, gene editing, chemical/radiation-based mutagenesis and hybridization. For transgenic lines, describe the transformation method, the number of independent lines analyzed and the generation upon which experiments were performed. For gene-edited lines, describe the editor used, the endogenous sequence targeted for editing, the targeting guide RNA sequence (if applicable) and how the editor was applied.*
Authentication	*Describe any authentication procedures for each seed stock used or novel genotype generated. Describe any experiments used to assess the effect of a mutation and, where applicable, how potential secondary effects (e.g. second site T-DNA insertions, mosiacism, off-target gene editing) were examined.*
