# Supplementary material for: Multicohort analysis unveils axon guidance pathways linking small for gestational age to spirometric restriction
Source: Nat Commun. 2026 May 2;17:5952. doi: 10.1038/s41467-026-72490-w (PMC13342533; doi:10.1038/s41467-026-72490-w)
Supplement: Supplementary file 3 — Transparent Peer Review file [file 41467_2026_72490_MOESM3_ESM.pdf]

nature portfolio

Peer Review File

Multicohort analysis unveils axon guidance pathways linking small for gestational age to spirometric restriction.

Corresponding Author: Dr James Read

This file contains all reviewer reports in order by version, followed by all author rebuttals in order by version.

Version 0:

Reviewer comments:

Reviewer #1

(Remarks to the Author)
This is a well-written and ambitious multicohort study that integrates proteomics, GWAS, and scRNA-seq data to explore molecular mechanisms linking small for gestational age (SGA) status at birth to long-term pulmonary outcomes. The authors present compelling evidence that a subgroup of SGA individuals exhibits an axon guidance-related proteomic signature at birth, which is later associated with spirometric restriction in adolescence and adulthood. The use of diverse human cohorts, orthogonal validation in GWAS datasets, and functional confirmation via a fetal sheep model adds significant translational depth. Overall, this work is a valuable contribution to the understanding of developmental origins of chronic respiratory disease.

Major Concerns:
1.Limited Signature Preservation Across Cohorts: One of the central limitations of the study is the poor preservation of SGA-associated proteomic signatures between cohorts. While the authors address this through “majority voting” PLS feature preselection (Figure 1 should indicate how many aptamers those were) and consensus clustering, the variability of differential expression and pathway enrichment analyses across cohorts (e.g., axon guidance in TCRS and COAST vs. immune-related pathways in CCCEH and IIS) raises concerns about the generalizability of the identified molecular endotypes. It would strengthen the manuscript if the authors could include to test a sparse multivariable classification model on batch-(cohort)-corrected data to demonstrate whether SGA cases can globally be differentiated from AGA. It seems that the TCRS cohort is the most different from the others (from PCA analysis), and it is also the oldest cohort. Possible reasons for this should be further elucidated in the limitations section. Do the other cohorts (excluding the TCRS cohort) yield a common set of features (aptamers)?

2.Link between the two proteomic datasets: The study uses two large proteomic datasets. Is there overlap between the individuals in the birth cohort and the childhood/adulthood cohorts? If so, the authors should include a correlation analysis of aptamer measurements between the two time points. If not, the analysis should still include the exact overlap in differentially abundant aptamers between the two time points. Finally, the authors could attempt to predict lung function parameters from the identified aptamers (e.g., childhood/adulthood timepoints).

3.Analysis of the proteomic datasets for the childhood/adulthood cohorts:

-The individual (per aptamer) linear models to find associations with lung function parameters seems inappropriate for such a high-dimensional dataset. Multivariable modeling should be used to ensure statistical robustness.

-The authors postulate an axon guidance-related subtype for SGA from the proteomic dataset at birth. However, in the analysis of the second phase (childhood/adulthood) of the study, this newly identified subtype split is not included anymore. Should not logically also roughly one third of the later in life cohort be related to axon guidance proteins, while the other two thirds are heterogeneous/not associated with axon guidance proteins? How do the authors explain that in the later in life cohort, axon guidance aptamers are now differentially abundant across all samples? It would be worth analyzing the later-in-life phase in parallel to the after-birth phase, also applying the same clustering to the later-in-life samples, and attempting to reproduce a similar split in Group 1 and Group 2 SGA.

Minor Suggestions:

•Please state in the main text with confounding variables were corrected for using SVA .
•In the protein networks, please highlight proteins and pathways mentioned in the text.
•In Suppl. Figure 8, please normalize the y-axis to the same range across plots for better comparability.
•Line 118: Please state which supervised clustering technique was used.

(Remarks on code availability)
The code has been made available and is appropriate.

Reviewer #2

(Remarks to the Author)
This manuscript presents a well-executed and conceptually innovative analysis linking small for gestational age (SGA) birth status to later-life spirometric restriction via dysregulation of axon guidance pathways. The study further supports this pathway by integrating genome-wide association studies (GWAS) related to lung function and by corroborating molecular signatures in a fetal sheep model using single-cell transcriptomics. The robust, multi-cohort design and the inclusion of orthogonal validation are major strengths. By integrating proteomic, genetic, and animal model data, the authors propose a mechanistic framework for understanding how early-life molecular signatures may underpin multiorgan morbidity, with a focus on pulmonary function.
RESULTS:
- The study includes 207 cord blood proteomic samples and 139 later-life peripheral blood samples across five well-characterized U.S. birth cohorts. The sample size is fairly small for proteomics cohort analysis, but adequate given the inclusion of later-life peripheral blood samples.
- The distinct molecular endotype (Group 1 SGA), present in ~1/3 of SGA subjects, is identified via consensus clustering and characterized by upregulation of axon guidance-related proteins. This subgroup was reproducibly detected across diverse cohorts, enhancing confidence in the biological relevance.
- The association between the molecular profile and the spirometric restriction later in life is compelling. It is supported by longitudinal proteomic data and GWAS-based evidence linking axon guidance gene loci to FEV_1 and FVC.
- The use of a sheep model of fetal growth restriction offers orthogonal support.
SIGNIFICANCE:
THis study addresses a knowledge gap, why /how SGA is correlated to longterm risk for respiratory issues and broad organ involvement. While previous studies have reported associations between SGA and chronic disease, they did not identify a pathway. The focus on axon guidance pathways is novel and extends the conversation beyond the neurological context. They may functionas mediators of respiratory and potentially multiorgan outcomes in SGA individuals. Prior studies have identified the clinical risks of SGA birth (e.g., Voraphani et al., Lancet Respir Med 2022), but the molecular underpinnings remain largely unexplored. Therefore, this is a meaningful step forward in developmental biology and may direct future research in pediatric pulmonology and molecular epidemiology.
CONCLUSIONS:
The core conclusions are well supported by the data and the clustering methods reveal a reproducible proteomic signature for Group 1 SGA infants. This subgroup is linked to pulmonary function measures across multiple cohorts. The GWAS enrichment analysis substantiates the genetic relevance of the pathways, while the animal model offers an independent layer of biological plausibility. That said, the link from axon guidance pathway alteration in the perinatal period to adult lung function is largely correlative at this stage. The manuscript appropriately acknowledges that there is a need for functional studies to establish causality more directly.
DATA ANALYSIS:
Data analysis approach is generally strong and relies on contemporary statistical methods.
METHODOLOGY:
Manuscript meets the expected standards for multi-omic epidemiologic research. The cohort size is acceptable and provides sufficient statistical power. The animal model and GWAS components are thoughtfully designed. The analytic workflow is clearly described, and cluster stability is well assessed using CDF plots and consensus matrices.
REPRODUCIBILITY:
The authors have committed to open science by making code and data available.
CONCLUSION:
Overall, this is a solid and interesting manuscript that provides new clues about SGA and lung risk. The introduction of a novel molecular axis reporting the relevance of axon guidance pathway dysregulation in the pathogenesis of SGA-related lung function impairment. The integration of multi-cohort human data, genomics, and animal models is methodologically strong and conceptually original. The study is well-suited for publication pending minor revisions.
I suggest that addressing the following issues would strengthen the manuscript and clarify interpretation for readers.

MAJOR ISSUES:
1.Characterization of Group 2 SGA:The characterization of Group 2 SGA as unclear is a recognized limitation. Expanding on what distinguishes Group 2 biologically and clinically, and discussing possible alternative mechanisms or outcomes, would improve the manuscript's depth.
2. Clarification of Longitudinal Linkage: The longitudinal classification limitation is important. Since Group 1/Group 2 designations exist only in the cord blood cohort and few subjects have paired later-life data, conclusions about individual-level longitudinal trajectories should be clearly qualified as group-level associations. This limitation is common in such cohort studies but needs explicit discussion.
3. Data Harmonization Methods: Describing harmonization of cohort data (handling of batch effects and differences such as plasma vs serum) is essential for reproducibility and transparency, especially in multi-cohort omics studies. Description of

how clinical or demographic data were harmonized across cohorts (variable definitions, coding consistency, or data harmonization protocols for: Birthweight/SGA classification, Maternal or perinatal variables, Environmental exposures, etc. Use of centralized data curation or harmonization standards like CDISC, OMOP, or similar.
4. Integration of Sheep Model Data: The sheep model's small sample size (n=2 per group) means its findings should be framed as exploratory and supportive rather than confirmatory. Acknowledging the limited inference from this model would balance enthusiasm with caution.

MINOR ISSUES:
1.Terminology Consistency: Consistency in terminology (eg.: "endotype," SGA/AGA, spirometric notations like %FEV1/FVC) helps avoid confusion.
2.Confounders: clarify how potential confounders (e.g. perinatal exposures or maternal factors) were accounted for in each birth cohort.
3.Generalizability Discussion: potential applicability or differences in other populations (non-U.S.) would increase generalizability awareness.
4. Please provide more details on randomization and blinding in sample selection.

(Remarks on code availability)
Yes, the code provides a sufficient README file.

Reviewer #3

(Remarks to the Author)
Read et al, on behalf of the CADRE investigators, reports a series of bioinformatic approaches identifying axon guidance proteins as a novel pathway that is perturbed in a subset of SGA newborns, and proceed through a series of bioinformatic approaches to build the argument that this pathway likely contributes to impaired lung function in individuals who were born SGA. The initial identification of the axon guidance pathway was based on proteomics analysis of cord blood from term-born SGA and AGA infants across five asthma and allergy birth cohorts from the US. The authors then demonstrate that expression of this protein cluster remains perturbed in children and adults born SGA, in association with spirometric restriction, and that key genes in the cluster are associated with spirometry data across population GWAS studies. Finally, they use single cell RNA-Seq analysis to validate that the axon guidance pathway is expressed in multiple fetal cell types during late gestation in sheep, with multiple genes in this pathway differentially expressed in control fetuses and fetuses from an experimental model of severe fetal growth restriction. These multiple strands of evidence are brought together to demonstrate likely functional importance of this pathway by mapping four axon guidance-related genes that are identified via all three approaches, that this, they are (i) are part of the gene cluster dysregulated in SGA infants, and (ii) identified in GWAS as predictors of lung function and (iii) expressed during development of the sheep fetal lung.

This is a highly original line of enquiry and study. Others have shown associations between fetal growth and postnatal lung function, but this is the first identification of the axon guidance pathway as a potential underlying mechanism.

The approach is novel and comprehensive but some limitations should be acknowledged. Firstly, because identification of the differentially expressed pathway was made in cord blood of term-born samples, the altered expression may not be causal. For example, could altered pathway expression be a compensatory response, or downstream of altered lung branching that has occurred earlier in gestation. It is not entirely clear from the data in the sheep model (Figure 5) whether the same pathway and gene set is dysregulated by experimental FGR., rather than the sheep aspect only demonstrating expression in the fetal lung during development.

It is not correct (abstract, introduction, discussion) to state that underlying mechanisms for associations between SGA and later disease are unknown. Certainly, not all mechanisms are understood, but this fails to acknowledge a large body of literature demonstrating mechanisms linking SGA and later disease, e.g. reduced nephron number and elevated postnatal blood pressure.

It is intriguing that only about a third of the term-born SGA infants exhibit altered expression of this gene cluster. The lower birth weight and maternal weight gain in this group might suggest more severe SGA. Do you have any data for fetal growth curves (e.g. repeated ultrasound measures) or placental phenotype that might help to define this group more specifically? Can you predict which SGA infants would be in this cluster, based on phenotypic markers, in order to target interventions based on this pathway to that subgroup?

The strength of evidence and uncertainty in the final conclusions that are drawn is appropriately captured in the final paragraph.

Minor suggestions:

• Line 65: Suggest insertion of "at" before "term"
• Lines 67-68: To justify this argument more completely, it would be good to include evidence for the strength of relationship between spirometry and CVD, as you have done between SGA and spirometry.
• The rationale for this study at lines 72-76 does not seem to justify the present study where you are measuring expression of pathways AFTER the events discussed, so cannot argue causality, i.e. that the axon guidance pathways would underlie the changes in anabolic hormone abundance.

• Results, lines 94-95: Could you please clarify whether these are the SAME individuals as those in which cord blood studies were done? It would be extremely powerful to be able to demonstrate longitudinal persistence of the proteomic dysregulation.
• Line 98: lower not reduced – comparison between groups rather than change over time.
• Line 224: Although this is stated in methods, it would be helpful to the reader to provide the timing of sample collection (in days and as a % of term) for these sheep studies.
• Lines 255-258: It may be over-interpretation to argue that altered expression of these proteins plays a role in impaired lung growth in SGA, since the protein abundance is measured AFTER the majority of lung growth has occurred, and is in peripheral blood, not in the lung itself.
• Discussion paragraph starting at line 288: Have you considered the possibility that axon guidance protein expression may alter lung function, not only morphogenesis? For example, altered nervous system connectivity/function within the lung might modulate airway tone/constriction and hence spirometry outcomes. These may not necessarily be epigenetic mechanisms – for example if innervation of airways is impaired during development then this would persist.
• Line 310: Suggest inserting “only” before “a small number”
• Findings of laminin as a key candidate for SGA-impacts on lung function is intriguing given evidence for thickening of the basement membrane in children who go on to develop asthma (Payne et al 2003 PMID: 12502479), and the increased risk of asthma in low birth weight individuals (Hassan et al 2021 PMID: 34167585).
• Methods, study subjects: It is evidence from Table 1 that you have selected a term-born cohort, which is a strength by removing impacts of prematurity. Could you please state this selection criteria within this methods section so that it is obvious to the reader?
• Figure 1 – can you avoid changing the names/terminology for the SGA sub-group with altered proteomic patterns – referred to as both Group 1 SGA and Cluster A SGA within Figure 1 legend. This subgroup is also named AG-SGA elsewhere in the paper – it will help the reader follow this complex study if the names are consistent throughout.
• Figure 1Ai. The overlap of the legend with the hierarchical diagram implies that the two are associated. Can you move these so that the legend is not overlaid on the tree diagram?
• Figure 2: could you add a dotted line around the section of Figure 2 Aii that is recoloured in the box/inset, for clarity? Line 571, should this be “red” not “read”? Why not provide breakdown in panel C for the Module 1 aptamers as well?
• Figure 3: this is very hard to read in places due to the extremely small font size. This could be increased in places such as axis labels. P-value legends on panels D are impossible to read!
• The much smaller impact of SGA on lung function parameters when corrected for height etc (Fig 3 A vs Fig 3 B) suggests that much of the impact of SGA on lung function is a reflection of overall impacts on growth. Is this interpretation reasonable?
• Figure 4: Consider adding x axis labels on panels Ai and ii, and a title label on Aii as you have done for Ai (GWAS hits associated with…)
• Figure 5: Is the p-value for Panel E for the comparison between FGR and control? Could you clarify please? Throughout legend – higher/lower rather than decreased/increased to reflect study design – not longitudinal. It would be helpful to state the gestational age at sample collection in the legend. White text on red box in panel A is very hard to read.

(Remarks on code availability)

Reviewer #4

(Remarks to the Author)
I co-reviewed this manuscript with one of the reviewers who provided the listed reports. This is part of the Nature Communications initiative to facilitate training in peer review and to provide appropriate recognition for Early Career Researchers who co-review manuscripts.

(Remarks on code availability)

Reviewer #5

(Remarks to the Author)
Comments to the Authors
Overall Assessment
This is an ambitious and innovative study that combines multicohort proteomics, genetic enrichment analyses, and a fetal sheep model to propose axon guidance pathways as a mechanistic link between restricted fetal growth and reduced lung function in later life. The breadth of evidence is impressive, and the supplementary material provides additional methodological detail. At the same time, several key issues regarding methodological robustness, cohort heterogeneity, and interpretation require attention
Major Comments
1. Cohort heterogeneity
The cohorts differ substantially in demographics, recruitment strategies, and racial/ethnic composition, as shown in Supplementary Tables 1–2. Supplementary Figures 1 and Extended Methods Figure 2 demonstrate that cohort of origin is the dominant source of variation in proteomic data. Although surrogate variable analysis was applied, the extent to which this adjustment fully removes confounding remains unclear. Additional sensitivity analyses in more homogeneous subgroups would strengthen confidence in the robustness of the findings.
2. Definition and stability of SGA subgroups

The identification of “Group 1 SGA” relies on a variable importance threshold (VIP > 1.5 in ≥3 cohorts) that appears somewhat arbitrary. Please provide evidence that the results are robust to alternative thresholds.
Supplementary Table 3 indicates that only one-third of SGA infants fall into Group 1. The stability of this subgrouping across resampling should be quantified, for example using bootstrap or consensus stability measures.
3. Peripheral blood associations
Later-life associations between proteins and spirometry outcomes (Supplementary Table 4, Figures 10–11) are based on small sample sizes and wide age ranges. Given the potential for age-related confounding in protein expression, please provide more direct acknowledgment of the limitations and, if possible, quantify statistical power.
4. GWAS enrichment
Supplementary Figure 12 shows overlap between axon guidance genes and pulmonary function GWAS loci, but the proportion is modest (~5%). Please conduct a formal enrichment analysis to determine whether the overlap is greater than expected by chance.
5. Sheep model
The fetal growth restriction sheep model adds valuable biological support but is severely underpowered (n=2 per group). Results should be presented as preliminary and exploratory. Consider addressing whether sex differences were explored, as these are known to influence outcomes in SGA.
6. Multiorgan interpretation
The scRNA-seq data provide interesting insights across brain, heart, and lung. However, the strongest empirical evidence relates to lung outcomes. Claims regarding multiorgan morbidity should be moderated or supported with additional evidence.

Minor Comments
1. The manuscript is dense, and some sections of the results could be simplified for clarity. Consider shifting detailed pathway outputs to the supplementary material and emphasizing the most consistent findings in the main text.
2. Sample sizes should be reported directly in all figures, including Supplementary Figure 10.
3. The terms “Group 1” and “Group 2 SGA” are functional but not intuitive. Consider using more descriptive labels (e.g., “axon guidance–associated SGA”).
4. Some supplementary analyses (e.g., Supplementary Figure 15) present nominal p-values. Please ensure that multiple testing corrections are applied consistently, or clearly distinguish between corrected and uncorrected results.
5. The rationale for selecting seven clusters in consensus clustering (Extended Methods Figure 6) should be summarized in the main text.
6. The merging of serum and plasma samples across cohorts should be explicitly justified.
7. Please provide a more explicit statement regarding management of potential conflicts of interest given several authors’ industry affiliations.
Recommendation: This study is promising, but substantial revisions are needed to strengthen the methodological robustness and to temper interpretation of findings. Addressing these concerns will substantially improve the impact and credibility of the work.

(Remarks on code availability)

Version 1:

Reviewer comments:

Reviewer #1

(Remarks to the Author)
We thank the authors for their thorough and thoughtful responses. All comments have been adequately addressed, and the revised manuscript is significantly improved. We have no further substantive concerns.

(Remarks on code availability)

Reviewer #2

(Remarks to the Author)
The authors have engaged carefully with the first-round reviews and have addressed the main methodological and interpretational issues raised in previous comments. The additional analyses and the expanded methods and limitations sections make the work notably more transparent and robust, and the remaining points are largely minor clarifications of wording and internal cross-referencing. In particular, the clarification that nonAG-SGA is indistinguishable from AGA on the basis of the available proteomic and clinical data, the explicit restriction of longitudinal claims to group-level associations, and the clearer framing of the sheep model as exploratory all improve the balance of the manuscript.

1. Significance and originality
In the revised version, the authors more clearly delineate the limitations around nonAG-SGA and longitudinal inference, which appropriately constrains but does not diminish the central conceptual advance.

2. Technical soundness and rigor

The authors now also clearly acknowledge the limited sample size in the later-life cohorts and, in particular, the very small sheep model (n=2 per group), and consistently present the animal data as exploratory and preliminary.

3. Clarity and quality of presentation
Although the manuscript is still information-dense, the revisions have improved readability through more consistent terminology (AG-SGA vs nonAG-SGA, “endotype”, spirometric notation) and clearer figure labelling and layout. The Methods and Extended Methods sections now give a much clearer account of cohort harmonization, the use of SVA, clustering choices and power considerations, and sample sizes are reported more systematically in figures. A short sentence in the main text explicitly pointing to the serum–plasma comparability analysis in the Extended Methods would further reassure readers about the decision to merge these samples.

4. Reproducibility and data/methods transparency
The authors have considerably strengthened the description of data harmonization, including the CREW/CADRE processes, the iterative development of harmonized variables and verification by cohort data managers. The extended methodological description now covers model specification, clustering parameters, VIP-threshold robustness, power simulations, GWAS enrichment testing, randomization of sample order and the rationale for combining serum and plasma. Code and data availability appear to be in line with current standards for omics studies, including provision of resources via a public repository, contingent on final curation at acceptance.

(Remarks on code availability)

Reviewer #3

(Remarks to the Author)
Thank you for the detailed responses to comments from all reviewers. My only remaining major suggestion is that the temporal relationships between proposed mechanisms (lung remodelling) and sample collection (cord blood or postnatal blood) still need to be noted. Although a comment has been added (lines 373-4) to acknowledge associations rather than causality, the fact that proteomic analysis was conducted on samples collected AFTER completion of the proposed underlying mechanisms should be acknowledged explicitly.

(Remarks on code availability)

Reviewer #4

(Remarks to the Author)
I co-reviewed this manuscript with one of the reviewers who provided the listed reports. This is part of the Nature Communications initiative to facilitate training in peer review and to provide appropriate recognition for Early Career Researchers who co-review manuscripts.

(Remarks on code availability)

Reviewer #5

(Remarks to the Author)
Thank you for providing the revised manuscript and the rebuttal letter. The manuscript has been substantially improved, and the major concerns raised during the initial review have been addressed effectively.

In particular, the added multivariable analyses, clearer treatment of inter-cohort variability, explicit discussion of longitudinal limitations, and more restrained interpretation of causality significantly strengthen the work. The definition and presentation of the axon-guidance–associated SGA endotype are now consistent and well justified, and the positioning of the sheep model as exploratory is appropriate.

I do not have additional major comments.

(Remarks on code availability)

Open Access This Peer Review File is licensed under a Creative Commons Attribution 4.0 International License, which permits use, sharing, adaptation, distribution and reproduction in any medium or format, as long as you give appropriate credit to the original author(s) and the source, provide a link to the Creative Commons license, and indicate if changes were made.
In cases where reviewers are anonymous, credit should be given to 'Anonymous Referee' and the source.
The images or other third party material in this Peer Review File are included in the article's Creative Commons license, unless indicated otherwise in a credit line to the material. If material is not included in the article's Creative Commons license and your intended use is not permitted by statutory regulation or exceeds the permitted use, you will need to obtain permission directly from the copyright holder.
To view a copy of this license, visit https://creativecommons.org/licenses/by/4.0/

We thank the reviewers for their thoughtful and constructive evaluation of our manuscript. We are encouraged by the reviewers appreciation of the strengths of the work, including the originality of the study design, the integration of proteomic, genomic, and experimental sheep model evidence, and the novel conceptual framework investigated. We also note the general enthusiasm regarding our manuscripts overall significance and suitability for publication.

At the same time, we appreciate the important methodological and interpretational concerns raised by the reviewers. We have made substantial revisions to address the reviewers' concerns and suggestions, which include several additional analyses and expansion of the limitations section, that we believe have strengthened the manuscript. Below is a point-by-point response to each reviewer's major and minor comments. We thank the reviewers again for strengthening the contribution and impact of this work.

Point-by-point response

[Reviewer comments in blue]

Reviewer #1

Major comments

1) Limited Signature Preservation Across Cohorts: One of the central limitations of the study is the poor preservation of SGA-associated proteomic signatures between cohorts. While the authors address this through "majority voting" PLS feature preselection (Figure 1 should indicate how many aptamers those were) and consensus clustering, the variability of differential expression and pathway enrichment analyses across cohorts (e.g., axon guidance in TCRS and COAST vs. immune-related pathways in CCCEH and IIS) raises concerns about the generalizability of the identified molecular endotypes. It would strengthen the manuscript if the authors could include to test a sparse multivariable classification model on batch-(cohort)-corrected data to demonstrate whether SGA cases can globally be differentiated from AGA. It seems that the TCRS cohort is the most different from the others (from PCA analysis), and it is also the oldest cohort. Possible reasons for this should be further elucidated in the limitations section. Do the other cohorts (excluding the TCRS cohort) yield a common set of features (aptamers)?

   Thank you for the suggestions. We have added the number of aptamers (n=102) identified form the PLS selection to the Figure 1 legend (line 599). To demonstrate that SGA can be differentiated from AGA globally, we applied sparse Partial Least Squares discriminatory analysis (sPLA-DA) as a multivariate classification model using the mixOmics R package. This approach successfully stratified SGA from AGA samples without noticeable clustering by Cohort or Sex (See below). 5-fold cross validation (permutated 200 times) of the model exhibited an AUC-ROC of 0.874. These results

have been added to Supplementary figure 2, with corresponding text added to the Results section (lines 114-115) and extended methods (page 40).

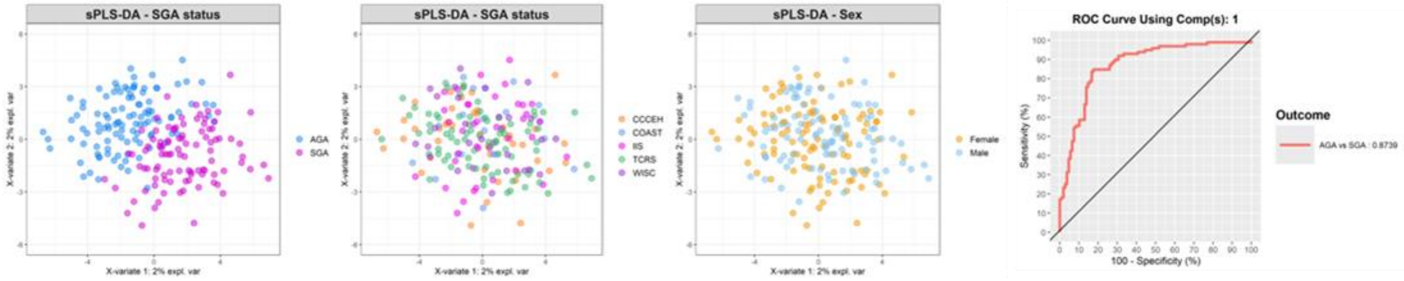

Additionally, we have added the following text (lines 374-380) to the limitations section to further highlight the inter-cohort variation: "*Further, the cohorts used in this study were collected across several decades employing different collection protocols, undoubtably contributing to the substantial variation in proteomic profiles observed between cohorts (particularly TCRS). Whilst the influence of this variation could be effectively removed with surrogate variables, purpose-designed studies to confirm and extend these findings would benefit from comprehensive consideration of all anticipated sources of unwanted variation in their design*"

2) Link between the two proteomic datasets: The study uses two large proteomic datasets. Is there overlap between the individuals in the birth cohort and the childhood/adulthood cohorts? If so, the authors should include a correlation analysis of aptamer measurements between the two time points. If not, the analysis should still include the exact overlap in differentially abundant aptamers between the two time points. Finally, the authors could attempt to predict lung function parameters from the identified aptamers (e.g., childhood/adulthood timepoints).

There were 12 individuals who had samples available at both the cord blood and later life collection timepoints. The cohorts employed in this study were each collected for a specific purpose and have facilitated many independent investigations for decades, many of which used blood sample aliquots. Therefore, the subjects available for the present multi-cohort study were those with remaining sample aliquots.

As suggested, correlation analysis between the 12 individuals with samples available at both timepoints showed aptamer expression is strongly correlated (Spearman's rho = 0.904, $p < 2.2\times10^{-16}$). We have added this analysis to Supplementary figure 10, with corresponding text added to the results section (lines 195-198). Additionally, we note that the cord blood and later life peripheral blood proteomic profiles were generated with the same SomaScan aptamer panel, and so the constituent aptamers were entirely comparable between timepoints.

As suggested, we employed an elastic net regression model to predict lung function

parameters of aptamer expression from child/adulthood timepoints, restricted to aptamers identified in the cord blood analysis. This analysis demonstrated moderate-to-good prediction of lung function measurements and has been added to Supplementary figure 11, and is discussed further in the following, related comment.

3) A) Analysis of the proteomic datasets for the childhood/adulthood cohorts: The individual (per aptamer) linear models to find associations with lung function parameters seems inappropriate for such a high-dimensional dataset. Multivariable modeling should be used to ensure statistical robustness.

We thank the reviewer for the suggestion, and agree that using a multivariate approach will ensure robustness of the findings. In the manuscript, we have retained the univariate (linear model) results, as they facilitate our aim to identify the individual aptamers (tested separately). However, we have also added an additional multivariate approach (elastic net regression), as suggested. The overall interpretation of the multivariate approach aligns with that of the univariate one, demonstrating moderate-to-good prediction of FEV_1 and FVC, but not the FEV_1 percentage of FVC (e.g., R^2 values of 0.502, 0.691, and 0.133, respectively, on the test set (the held-out samples not used in model training). As mentioned above, the multivariate approach was also applied to aptamers from the cord blood-derived axon guidance signature, with similar results.

The analysis has been added to Supplementary figure 11, with corresponding text added to the results section (lines 216-224) and extensively detailed in the extended methods section (page 48-49).

B) The authors postulate an axon guidance-related subtype for SGA from the proteomic dataset at birth. However, in the analysis of the second phase (childhood/adulthood) of the study, this newly identified subtype split is not included anymore. Should not logically also roughly one third of the later in life cohort be related to axon guidance proteins, while the other two thirds are heterogeneous/not associated with axon guidance proteins? How do the authors explain that in the later in life cohort, axon guidance aptamers are now differentially abundant across all samples? It would be worth analyzing the later-in-life phase in parallel to the after-birth phase, also applying the same clustering to the later-in-life samples, and attempting to reproduce a similar split in Group 1 and Group 2 SGA

As mentioned in response to comment #2 above, only 12 of the individuals with childhood/adulthood samples (from the total 126 individuals) also had a cord blood sample available from the birth cohort. This is largely due to sample aliquots being used in previous studies from these cohorts. The 12 individuals with linked data have the following characteristics: 8 were from the TCRS & 4 were from the IIS cohorts, 8 were female & 4 were male, 11 were born SGA & 1 was born AGA, 3 were AG-SGA, 8 were nonAG-SGA, and 1 was AGA designated from their cord samples. We have added a

table displaying these characteristics for the 12 individuals to the extended methods section (page 32).

The small number of individuals with paired samples between birth and childhood/adulthood (and only one being AGA) means it was not possible to meaningfully analyze differences between collection timepoints in a paired manner, nor faithfully apply a retrospective assignment of Group1/Group 2 (AG-/nonAG-SGA) status (and identify the anticipated 30%/70% split). Thus, it was not possible to assess the childhood/adult samples in parallel. Rather, we assessed whether axon guidance-related proteins were also associated with lung function in later life in (largely) different individuals from the similar cohort contexts.

Presumably then, the associations found are either driven by the ~30% that would have been AG-SGA (group 1) at birth, or conceivably that axon guidance proteins are broadly associated with lung function measurement generally, and those with the AG-SGA endotype exhibit dysregulation of the proteins from early life.

We have added the following sentence to the limitations paragraph of the discussion section (lines 385-389) to further clarify these points: "*Also, only 12 individuals had samples available in both the birth and childhood/adult timepoints, and as such it was not possible to meaningfully analyze the data longitudinally in a paired manner, nor was it possible to faithfully designate AG-SGA or nonAG-SGA endotype status to individuals with only childhood/adult samples*".

Minor comments

1) Please state in the main text with confounding variables were corrected for using SVA
   We have added text in the results section stating SVA was used for correction for latent unwanted variation (line 108).
2) In the protein networks, please highlight proteins and pathways mentioned in the text.
   We have added dashed red circles around the cliques of interest mentioned in the main text in Figure 2D and Supplementary figure 6C, as suggested.
3) In Suppl. Figure 8, please normalize the y-axis to the same range across plots for better comparability.
   We have adjusted the y-axis for these plots in Supplementary figure 8A to display the same range for all cohorts, as suggested.
4) Line 118: Please state which supervised clustering technique was used.
   We have added that partial least squares was the supervised feature selection technique used in the results section (line 120)

Reviewer #2

Major comments

1) Characterization of Group 2 SGA:The characterization of Group 2 SGA as unclear is a recognized limitation. Expanding on what distinguishes Group 2 biologically and clinically, and discussing possible alternative mechanisms or outcomes, would improve the manuscript's depth.

   We found no significant difference in aptamer expression between nonAG-SGA (Group 2) status and AGA, and likewise observed no significant difference in available clinical variables. From this we conclude that the nonAG-SGA group are indistinguishable from the AGA group from our data.

   We have added text to the results section (lines 147-150) to state this clearly: "*No aptamers recorded an FDR-adjusted p value < 0.05 for the comparison between nonAG-SGA versus AGA, indicating that this subgroup of SGA individuals may be indistinguishable from AGA individuals based on cord blood protein measurements.*"

   We have also expanded the text in the limitations paragraph of the discussion (lines 380-385) for further clarity and point out future directions that may uncover the factors behind nonAG-SGA status, including "*Additionally, our study did not detect a consistent molecular pattern for nonAG-SGA status that can distinguish them from AGA status, and no substantial differences in the recorded clinical variables compared to AGA. Future studies investigating maternal genetics with comprehensive clinical variables recorded during pregnancy (such as nutrition, ultrasound biometric, placental function, and lifestyle factors) may elucidate the potential genetic and environmental factors precipitating nonAG-SGA status*"

   Additionally, although not related directly to nonAG-SGA, we have added additional text to the results (lines 143-147) and discussion (line 361-363) to comment on the downregulation of placenta/pregnancy-specific proteins observed in AG-SGA (Group 1 SGAs).

2) Clarification of Longitudinal Linkage: The longitudinal classification limitation is important. Since Group 1/Group 2 designations exist only in the cord blood cohort and few subjects have paired later-life data, conclusions about individual-level longitudinal trajectories should be clearly qualified as group-level associations. This limitation is common in such cohort studies but needs explicit discussion.

   We agree that this should be stated more explicitly in the manuscript. We have added the following additional text:

Limitations section of discussion (lines 385-389): “*Also, only 12 individuals had samples available in both the birth and childhood/adult timepoints, and as such it was not possible to meaningfully analyze the data longitudinally in a paired manner, nor was it possible to faithfully designate AG-SGA or nonAG-SGA endotype status to individuals with only childhood/adult samples*”

Methods section (lines 411-414): “*Few individuals had blood samples available from birth and childhood/adulthood timepoints, therefore it was not possible to conduct longitudinal analysis between paired cord blood and later life peripheral blood samples*”

Also, we have added additional text and a table to the Extended methods section (page 32) to detail the characteristics of the 12 samples that have pair samples at birth and in later life (related to major comment 3B from Reviewer 1, above)

3) Data Harmonization Methods: Describing harmonization of cohort data (handling of batch effects and differences such as plasma vs serum) is essential for reproducibility and transparency, especially in multi-cohort omics studies. Description of how clinical or demographic data were harmonized across cohorts (variable definitions, coding consistency, or data harmonization protocols for: Birthweight/SGA classification, Maternal or perinatal variables, Environmental exposures, etc. Use of centralized data curation or harmonization standards like CDISC, OMOP, or similar.

We have added the following text to the Extended methods section (page 32) to clarify the data harmonization.

The Children’s Respiratory and Environmental Workgroup (CREW) consortium within the ECHO program(1) was established in 2016. In 2023, CREW transitioned into the Children’s Allergy and Asthma Data Repository (CADRE) and continued to process data. Clinical data from 5 of the 12 longitudinal birth cohorts was used. The cohorts collected similar information from participant surveys using differently worded questions at various ages for different recall and follow-up periods, depending on each cohort’s protocol. Investigators and the data coordinating team at the University of Wisconsin developed harmonized variable definitions consistent across all cohorts for parental atopy using an iterative process. The data coordinating team then created the final harmonized parental atopy variables, which the cohort data managers verified. For all other variables, investigators developed harmonized definitions consistent across all cohorts, and then cohort data managers created and verified the final harmonized variables.

4) Integration of Sheep Model Data: The sheep model's small sample size (n=2 per group) means its findings should be framed as exploratory and supportive rather than confirmatory. Acknowledging the limited inference from this model would balance enthusiasm with caution.

   We thank the reviewer for the comment and agree that the sheep model was exploratory, and thus all findings should be treated as preliminary.

   We have added the following text to the Results section (lines 259-260) to temper the interpretation from the sheep model findings: "*We note that these findings were generated from a small exploratory study model, and thus we treat these results as preliminary.*"

   We have also added text to the discussion section relating to the sheep model (starting line 326) to frame the findings as exploratory/preliminary.

<u>Minor comments</u>

1) Terminology Consistency: Consistency in terminology (eg.: "endotype," SGA/AGA, spirometric notations like %FEV1/FVC) helps avoid confusion.

   We have made several changes to clarify the terminology used throughout the manuscript, as suggested. We have renamed Group 1 and Group 2 SGA status as AG-SGA and nonAG-SGA to be more specific throughout the manuscript. We have changed 'subgroup' to 'endotype' where appropriate as a specific term for a disease/condition manifestation that arises from separate molecular processes ('subgroup' still used in the context of hierarchical clustering). We have used %FEV1/FVC (the percentage of FEV1 of the total FVC) preferentially throughout, with the exception of analysis using the Global Lung Function Initiative (GLI) reference equations that use the FEV1/FVC ratio rather than percentage.

2) Confounders: clarify how potential confounders (e.g. perinatal exposures or maternal factors) were accounted for in each birth cohort.

The cohort used in this study were independently collected as prospective birth cohorts for, in general, allergic and respiratory disease (atopy, asthma, wheeze, etc), rather than for investigation of the pregnancy period *per se*. As such, many potential confounders (and potentially causal factors of SGA) were not recorded. This motivated our use of surrogate variate analysis, which can identify and protect the variation associated with a variable of interest (e.g., SGA status), while accounting for unwanted variation present in the data, whether its cause is known (e.g., cohort, sex) or not.

Even with comprehensive recording of all potential confounders, a data-driven approach to address unwanted variation in the data may be advisable as it can capture partial (nested) confounding. For example in this study, the relationship between cohort and ethnicity/race, e.g., TCRS is predominantly, but not exclusively, white Caucasian, whereas the CCCEH cohort (recruited from inner New York City) is exclusively African American or Hispanic.

The section describing surrogate variate analysis in the Extended methods section (from page 37) details this approach and includes examples before and after SVA adjustment, which shows effective removal of variation associated with known (and presumably unknown) variables with unwanted influence (PCA example with cohort shown below).

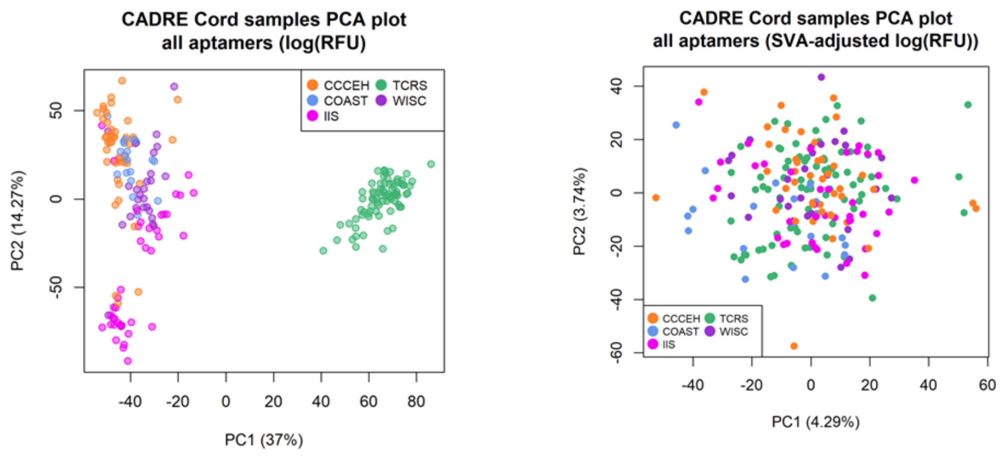

3) Generalizability Discussion: potential applicability or differences in other populations (non-U.S.) would increase generalizability awareness.

We have added the following sentence to the limitations section (line 389-391):

Finally, the study was performed in children born in the United States, and the applicability of our findings to populations with much higher incidence of SGA will require confirmatory studies.

4) Please provide more details on randomization and blinding in sample selection

The selection of individuals from each cohort was dictated by those that had remaining sample aliquots. Of these, care was taken to ensure roughly even representation with regard to SGA status and sex for each cohort.

For data generation, the TCRS cohort proteomic profiles were generated initially (with cord blood and adult peripheral blood profiles generated in separate runs). The remaining samples from all other cohorts were subsequently generated (again with cord blood and later life peripheral blood samples in separate runs). The reason for this was that the TCRS cohort was the initial focus of the study, with additional cohort introduced to broaden the generalizability of the findings.

The sample order was randomized and checked for even distribution with respect to SGA status and sex to ensure there was no inadvertent grouping by SGA/sex in the sequence order. The samples were then sent for data proteomic sequencing (SomaScan) by SomaLogic. We've added text to the Extended methods (page 33) to state this.

Reviewer #3

Major comments

1) Firstly, because identification of the differentially expressed pathway was made in cord blood of term-born samples, the altered expression may not be causal. For example, could altered pathway expression be a compensatory response, or downstream of altered lung branching that has occurred earlier in gestation. It is not entirely clear from the data in the sheep model (Figure 5) whether the same pathway and gene set is dysregulated by experimental FGR., rather than the sheep aspect only demonstrating expression in the fetal lung during development.

   We added a disclaimer regarding causality in the limitations sections (line 373-374).

2) It is not correct (abstract, introduction, discussion) to state that underlying mechanisms for associations between SGA and later disease are unknown. Certainly, not all mechanisms are understood, but this fails to acknowledge a large body of literature demonstrating mechanisms linking SGA and later disease, e.g. reduced nephron number and elevated postnatal blood pressure.

   We edited the abstract and the introduction to acknowledge that there are already mechanisms explaining the association.

3) Do you have any data for fetal growth curves (e.g. repeated ultrasound measures) or placental phenotype that might help to define this group more specifically? Can you predict which SGA infants would be in this cluster, based on phenotypic markers, in order to target interventions based on this pathway to that subgroup?

   Unfortunately, these data (fetal growth curves/placenta phenotype) were not collected as part of the prospective birth cohorts used in this study, which are focused on the individuals from birth, rather than the mother/infant during pregnancy.
   We have mentioned in the discussion that future studies involving maternal genetics and pregnancy factors, including ultrasound biometrics and placental function (lines 382-385) (related to major comment 1 from Reviewer #2).

<u>Minor comments</u>

1) Line 65: Suggest insertion of “at” before “term”

   Change made.

2) Lines 67-68: To justify this argument more completely, it would be good to include evidence for the strength of relationship between spirometry and CVD, as you have done between SGA and spirometry.

   We added specific data on risk for CV disease, with additional reference, in the introduction (line 61).

3) The rationale for this study at lines 72-76 does not seem to justify the present study where you are measuring expression of pathways AFTER the events discussed, so cannot argue causality, i.e. that the axon guidance pathways would underlie the changes in anabolic hormone abundance.

   We added a disclaimer regarding causality in the limitations sections (line 373-374).

4) Results, lines 94-95: Could you please clarify whether these are the SAME individuals as those in which cord blood studies were done? It would be extremely powerful to be able to demonstrate longitudinal persistence of the proteomic dysregulation.

   In general, these were *not* the same individuals. Only 12 individuals had sample available at birth and at a childhood/adult timepoint. We have made several additions to the manuscript to clarify this point (please see comments 2 and 3B from Reviewer #1, and comment 2 from Reviewer #2).

5) Line 98: lower not reduced – comparison between groups rather than change over time.

Change made.

6) Line 224: Although this is stated in methods, it would be helpful to the reader to provide the timing of sample collection (in days and as a % of term) for these sheep studies.

We have added the gestational ages for elevated temperature and sample collection to the Results section (line 245) and added the percentage of term to the methods section (line 497). We have also made the diagram in Figure 5A more legible and added additional text to the Extended methods section (page 50).

7) Lines 255-258: It may be over-interpretation to argue that altered expression of these proteins plays a role in impaired lung growth in SGA, since the protein abundance is measured AFTER the majority of lung growth has occurred, and is in peripheral blood, not in the lung itself.

We added a disclaimer regarding causality in the limitations sections (line 373-374).

8) Discussion paragraph starting at line 288: Have you considered the possibility that axon guidance protein expression may alter lung function, not only morphogenesis? For example, altered nervous system connectivity/function within the lung might modulate airway tone/constriction and hence spirometry outcomes. These may not necessarily be epigenetic mechanisms – for example if innervation of airways is impaired during development then this would persist.

We considered this possibility but thought that it is not a potential alternative explanation for our findings because what is affected in SGA is the aerated size of the lung (i.e., FVC), whereas indices of airway smooth muscle tone and airflow (which are controlled by adrenergic and muscarinic innervation), e.g., FEV_1/FVC ratio remained largely untouched.

9) Line 310: Suggest inserting “only” before “a small number”

Change made.

10) Findings of laminin as a key candidate for SGA-impacts on lung function is intriguing given evidence for thickening of the basement membrane in children who go on to develop asthma (Payne et al 2003 PMID: 12502479), and the increased risk of asthma in low birth weight individuals (Hassan et al 2021 PMID: 34167585).

We added a sentence to the discussion (lines 350-352) acknowledging this mechanism, with suggested references.

11) Methods, study subjects: It is evidence from Table 1 that you have selected a term-born cohort, which is a strength by removing impacts of prematurity. Could you please state this selection criteria within this methods section so that it is obvious to the reader?

We have added that “All subjects assessed in this study were born at term” in the methods section of the main text (lines 415-416), and have added additional text to the Extended Methods (page 32) to clarify this point.

12) Figure 1 – can you avoid changing the names/terminology for the SGA sub-group with altered proteomic patterns – referred to as both Group 1 SGA and Cluster A SGA within Figure 1 legend. This subgroup is also named AG-SGA elsewhere in the paper – it will help the reader follow this complex study if the names are consistent throughout.

We have change “Group 1” to “AG-SGA” and “Group 2” to “nonAG-SGA” to be consistent throughout the manuscript (related to minor comment 3 from Reviewer #5).

13) Figure 1Ai. The overlap of the legend with the hierarchical diagram implies that the two are associated. Can you move these so that the legend is not overlaid on the tree diagram?

We have adjusted the legend so it doesn’t overlap the dendrogram.

14) Figure 2: could you add a dotted line around the section of Figure 2 Aii that is recoloured in the box/inset, for clarity? Line 571, should this be “red” not “read”? Why not provide breakdown in panel C for the Module 1 aptamers as well?

Thanks for the suggestions. We have added a dashed line around the regions of interest, and corrected the typo. The panel C plots for Module 1 (related to collogen) was shifted to a supplementary figure as it was not part of the main focus on axon guidance in the text, and to increase space/clarity in the main figure.

15) Figure 3: this is very hard to read in places due to the extremely small font size. This could be increased in places such as axis labels. P-value legends on panels D are impossible to read!

We have increased the size of the labels in Figure 3A&B, and have made the p value notation in Figure 3D more legible.

16) The much smaller impact of SGA on lung function parameters when corrected for height etc (Fig 3 A vs Fig 3 B) suggests that much of the impact of SGA on lung function is a reflection of overall impacts on growth. Is this interpretation reasonable?

We agree that the effect of SGA on unadjusted lung function reflects the known strong

global effect of SGA on growth, but the persistence of a significant association after adjustment for height suggests that there is a specific effect of SGA on lung growth as well.

17) Figure 4: Consider adding x axis labels on panels Ai and ii, and a title label on Aii as you have done for Ai (GWAS hits associated with…)

We have added/corrected these plot titles and labels, as suggested.

18) Figure 5: Is the p-value for Panel E for the comparison between FGR and control? Could you clarify please? Throughout legend – higher/lower rather than decreased/increased to reflect study design – not longitudinal. It would be helpful to state the gestational age at sample collection in the legend. White text on red box in panel A is very hard to read.

The p value shown in Figure 5E is the (-log10) adjusted-p value from the pathways analysis. The plot shows the adjusted-p values of 15 selected axon guidance pathways from the pathways enrichment analysis of the Differentially Expressed Genes (DEG) identified between FGR and control.
We have changed increased/decreased to higher/lower, added gestation age at sample collection (130-135 dGA) in the legend, and reconfigured the diagram in Figure 5A for better readability.

Reviewer #4

Reviewer #4 stated they co-reviewed with another reviewer as part of a Nature Communications initiative.

Reviewer #5

Major comments

1) Cohort heterogeneity. The cohorts differ substantially in demographics, recruitment strategies, and racial/ethnic composition, as shown in Supplementary Tables 1–2. Supplementary Figures 1 and Extended Methods Figure 2 demonstrate that cohort of origin is the dominant source of variation in proteomic data. Although surrogate variable analysis was applied, the extent to which this adjustment fully removes confounding remains unclear. Additional sensitivity analyses in more homogeneous subgroups would strengthen confidence in the robustness of the findings.

   We applied an additional sensitivity analysis to confirm that the findings from the cord blood protein profiles were robust to more homogenous sample groups and were not driven by a small number of outlying samples. The number of input samples for consensus clustering were restricted to more homogeneous subsets according to decreasing confidence regions (95%, 90%, 85%, 80%, and 75%) of the first two principal components. Consensus clustering on samples retained from each confidence region identified a cluster which was enriched for SGA samples and exhibited axon guidance related pathways from genes encoding dysregulated proteins between these SGA samples and all AGA samples, aligning with the findings using the full dataset. This analysis has been included in the Extended methods section (pages 44-45) of the revised manuscript.

2) Definition and stability of SGA subgroups. The identification of “Group 1 SGA” relies on a variable importance threshold (VIP > 1.5 in ≥3 cohorts) that appears somewhat arbitrary. Please provide evidence that the results are robust to alternative thresholds. Supplementary Table 3 indicates that only one-third of SGA infants fall into Group 1. The stability of this subgrouping across resampling should be quantified, for example using bootstrap or consensus stability measures.

   We tested alternative VIP thresholds during analysis of this project, but didn’t include this in the original manuscript. We used VIP thresholds of 1.0, 1.2, 1.3, 1.35, 1.4, 1.45, 1.5, 1.55, 1.6, and 1.7, and assessed the consensus clustering outcomes. With the exception of VIP=1.0 (too many aptamers included, n=1,962) and VIP=1.7 (too few aptamers, n=20), the clustering outcomes were comparable to VIP=1.5, exhibiting a distinct ‘Cluster A’ made up of a majority of SGA subjects (range 64.86%-85.19%).
   We have now added this analysis to the extended methods section (pages 42-43) of the re-submitted manuscript to rectify the omission, and have added text to the Methods section (lines 466-467) stating that testing of alternative VIP thresholds demonstrated similar consensus clustering outcomes.

   We also assessed the use of aptamers that were identified in any (≥1) and all (5) cohorts, and found that the number of included aptamers was either too lenient or stringent, respectively. We concluded that using aptamers identified in ≥3 cohorts (i.e.,

the majority) in this dataset balanced stringency with sensitivity to provide informative consensus clustering results.

With regard to consensus stability, the method used (ConsensusClusterPlus in R) produces stability measures for the analysis, which we have included in the extended methods section (page 42). In particular, a consensus matrix heatmap (extended methods figure 6.1C) shows the pairwise proportion of a given subject co-clustering with all other subjects for a given cluster from the 1,000 resamples, ranging from 0 (never clustered together) to 1 (always clustered together). We have altered the figure legend to more explicitly state that this is a measure of cluster stability across the 1,000 resamples.

3) Later-life associations between proteins and spirometry outcomes (Supplementary Table 4, Figures 10–11) are based on small sample sizes and wide age ranges. Given the potential for age-related confounding in protein expression, please provide more direct acknowledgment of the limitations and, if possible, quantify statistical power.

We have added the following text to the corresponding section in the Results (lines 224-225): “We note that the age of subjects from the three cohorts does not capture the full continuum between childhood and adulthood, and this is a limitation of the analysis”.

Additionally, in reply to comment 3 by Reviewer #1, to demonstrate the robustness of the results, we repeated this analysis with a multivariate approach (Elastic net regression), which produced complementary findings.

With regard to statistical power, whilst there is little consensus of the most appropriate power calculation for omics data, a simulation-based approach is often recommended. To estimate the statistical power for the later life associations between proteins and lung function measurements, we ran 2,000 simulations of normalized protein measurements (assuming normal distribution and unit variance) for 126 samples (the number available in the real dataset) with varying effect sizes. The power to detect differences were as follows:

Small effect size (0.025) - 19.85%
Small-to-moderate effect size (0.05) – 63.2%
Moderate-to-large effect size (0.075) – 87.8%
Large effect size (0.1) – 96.25%

This suggest that the sample size (n=126) is relative well powered to detect difference with moderate/large effect sizes, but is perhaps underpowered for smaller effect sizes. We have added this interpretation to the extended methods (page 47).

4) GWAS enrichment. Supplementary Figure 12 shows overlap between axon guidance genes and pulmonary function GWAS loci, but the proportion is modest (~5%). Please conduct a formal enrichment analysis to determine whether the overlap is greater than expected by chance.

   Thank you for the suggestion, we have added a formal enrichment analysis (Fisher's test) to the revised manuscript. The test showed that the overlap was greater than expected by chance given the total number of SNPs ($p = 1.325 \times 10^{-14}$, OR = 25.59, 95%CI = 12.26-51.42). We have updated the results section (lines 236-237) and Supplementary figure 12 to show this analysis.

5) Sheep model. The fetal growth restriction sheep model adds valuable biological support but is severely underpowered (n=2 per group). Results should be presented as preliminary and exploratory. Consider addressing whether sex differences were explored, as these are known to influence outcomes in SGA.

   As mentioned in the reply to comment 4 from Reviewer #2, we have added additional text to the Results (line 259-260) and discussion (starting line 326) section to frame these findings as preliminary/exploratory. The small number of donors for this analysis ensured we could not meaningfully explore sex differences. We have added a sentence in the Extended methods (page 54) to state this.

6) Multiorgan interpretation. The scRNA-seq data provide interesting insights across brain, heart, and lung. However, the strongest empirical evidence relates to lung outcomes. Claims regarding multiorgan morbidity should be moderated or supported with additional evidence.

   We have moderated the text to temper the interpretation of the multiorgan implication. Specifically, we have added "although larger studies are required to determine the extent to which this may be related to altered development of different organs and multiorgan comorbidity" to the Discussion (lines 336-337) and changed the final, concluding sentence in the discussion to reflect the need for future studies to determine the extent to which these results may be associated with multiorgan comorbidity (lines 397-399).

<u>Minor comments</u>

1) The manuscript is dense, and some sections of the results could be simplified for clarity. Consider shifting detailed pathway outputs to the supplementary material and emphasizing the most consistent findings in the main text.

   Thank you for the suggestion. We have removed several of the pathways analysis plots, instead referring to the Data supplement which contains the analysis outputs in full. We have also made several changes throughout the main and supplementary figures, as

suggested by other reviewers, to increase clarity.

2) Sample sizes should be reported directly in all figures, including Supplementary Figure 10.

We have added the sample sizes in plots/legends to all relevant main and supplementary figures.

3) The terms “Group 1” and “Group 2 SGA” are functional but not intuitive. Consider using more descriptive labels (e.g., “axon guidance–associated SGA”).

Thank you for the suggestion, we have changed “Group 1” to Axon guidance-associated SGA (AG-SGA) and “Group 2” to nonAG-associated SGA (nonAG-SGA) to be consistent throughout the manuscript.

4) Some supplementary analyses (e.g., Supplementary Figure 15) present nominal p-values. Please ensure that multiple testing corrections are applied consistently, or clearly distinguish between corrected and uncorrected results.

We have made several changes throughout the figure legends to state the p value adjustment method, or whether a nominal p value is presented, to clarify this.

5) The rationale for selecting seven clusters in consensus clustering (Extended Methods Figure 6) should be summarized in the main text.

We have added text summarizing the metrics assessed (CDF and consensus matrix) to determine the optimal number of clusters to the Methods section of the main text (lines 473-474).

6) The merging of serum and plasma samples across cohorts should be explicitly justified.

We calculated the correlation of mean expression for all aptamers/proteins between data generated from serum and plasma samples to assess the relative variation that this may have contributed. This showed that the data generated from serum and plasma samples was strongly (positively) correlated (non-parametric Spearman’s test; rho = 0.891, $p < 2.2 \times 10^{-16}$). Any differences in protein comparison would presumably be more sensitive to the cohort of origin (serum sample from the TCRS cohort), which was adjusted for with surrogate variables. Plots of this correlation are shown below (with mean expression (left) and ranked mean expression (right)), and these have been included in the Extended methods (page 33) with an explicit justification.

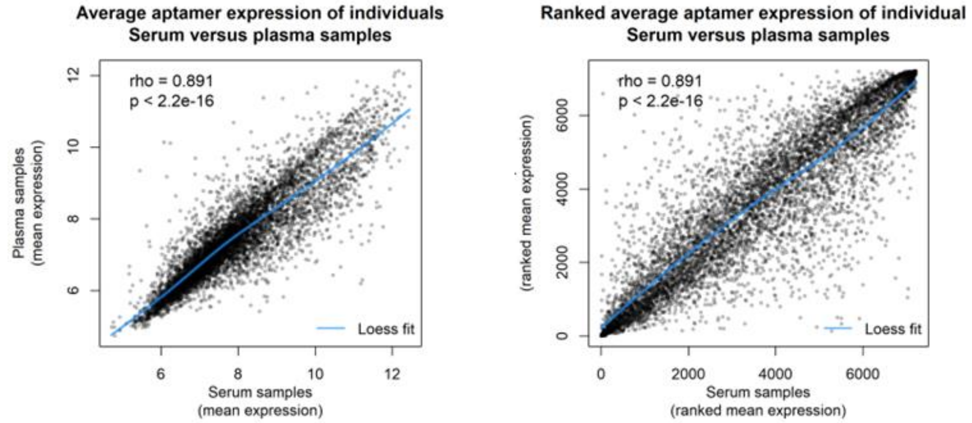

7) Please provide a more explicit statement regarding management of potential conflicts of interest given several authors' industry affiliations.

We reached out to the relevant authors, who have all confirmed that their university/institute has COI protocols in place and are all under active COI management plans relevant to their industry affiliations. Additional text has been added to the Author declarations/Conflict of Interest section to explicitly state this (lines 549-551).

In general, all university/institutes associated with this manuscript are based in the United States and are required to comply with the US Federal conflict of interest polices (e.g., from the Office of Science and Technology Policy) through various government branches (e.g., the National Institute of Health). A summary of the authors responses is provided below.

The University of Arizona supplies up-to-date information and protocols on Outside Interests, and actively manages conflict of interest and conflict of commitment managements plans, through the Office for Responsible Outside Interests. The University of Wisconsin (UW) has a Conflict of Interest Program that reviews and recommends management steps for any conflicts that are relevant to academic duties for any UW investigator that discloses a COI. Mount Sinai has a COI management system in place, and each individual is responsible for submitting their potential COI's on a regular basis. Harvard University has specific protocols in place to manage potential COIs, such as the Mass General Brigham's Policy for Interactions with Industry and Other Outside Entities. The Henry Ford Health's COI Policy establishes the guidelines for individuals to follow when considering external relationships, and the approval, review and reporting process. All investigators periodically have to certify their status regarding the Federal COI guidelines through the CITI program.

REVIEWERS' COMMENTS

Study author's comments in blue.

Reviewer #1 (Remarks to the Author):

We thank the authors for their thorough and thoughtful responses. All comments have been adequately addressed, and the revised manuscript is significantly improved. We have no further substantive concerns.

No further comments.

Reviewer #2 (Remarks to the Author):

The authors have engaged carefully with the first-round reviews and have addressed the main methodological and interpretational issues raised in previous comments. The additional analyses and the expanded methods and limitations sections make the work notably more transparent and robust, and the remaining points are largely minor clarifications of wording and internal cross-referencing. In particular, the clarification that nonAG-SGA is indistinguishable from AGA on the basis of the available proteomic and clinical data, the explicit restriction of longitudinal claims to group-level associations, and the clearer framing of the sheep model as exploratory all improve the balance of the manuscript.

1. Significance and originality
In the revised version, the authors more clearly delineate the limitations around nonAG-SGA and longitudinal inference, which appropriately constrains but does not diminish the central conceptual advance.

No further comments.

2. Technical soundness and rigor
The authors now also clearly acknowledge the limited sample size in the later-life cohorts and, in particular, the very small sheep model (n=2 per group), and consistently present the animal data as exploratory and preliminary.

No further comments.

3. Clarity and quality of presentation
Although the manuscript is still information-dense, the revisions have improved

readability through more consistent terminology (AG-SGA vs nonAG-SGA, “endotype”, spirometric notation) and clearer figure labelling and layout. The Methods and Extended Methods sections now give a much clearer account of cohort harmonization, the use of SVA, clustering choices and power considerations, and sample sizes are reported more systematically in figures. A short sentence in the main text explicitly pointing to the serum–plasma comparability analysis in the Extended Methods would further reassure readers about the decision to merge these samples.

For the final manuscript for publication, the entire Extended methods will be moved to the main text Methods section, in line with the Journals guidelines/request. Therefore, this point will be included in the main text.

4. Reproducibility and data/methods transparency
The authors have considerably strengthened the description of data harmonization, including the CREW/CADRE processes, the iterative development of harmonized variables and verification by cohort data managers. The extended methodological description now covers model specification, clustering parameters, VIP-threshold robustness, power simulations, GWAS enrichment testing, randomization of sample order and the rationale for combining serum and plasma. Code and data availability appear to be in line with current standards for omics studies, including provision of resources via a public repository, contingent on final curation at acceptance.

No further comments.

Reviewer #3 (Remarks to the Author):

Thank you for the detailed responses to comments from all reviewers. My only remaining major suggestion is that the temporal relationships between proposed mechanisms (lung remodelling) and sample collection (cord blood or postnatal blood) still need to be noted. Although a comment has been added (lines 373-4) to acknowledge associations rather than causality, the fact that proteomic analysis was conducted on samples collected AFTER completion of the proposed underlying mechanisms should be acknowledged explicitly.

We agree with this observation, with a caveat: we believe that we assessed these proteins not after the process *per se*, but at the end of it, while it still was occurring *in-utero.* We cannot exclude the possibility that what we're seeing at the end of the process may be different to what was occurring at the beginning of it.

For this reason, we have added an additional sentence to the discussion stating:

“Further, we generated data at the end of pregnancy and as such did not capture the putative mechanisms during earlier time points *in utero*

Reviewer #4 (Remarks to the Author):

I co-reviewed this manuscript with one of the reviewers who provided the listed reports. This is part of the Nature Communications initiative to facilitate training in peer review and to provide appropriate recognition for Early Career Researchers who co-review manuscripts.

No further comments.

Reviewer #5 (Remarks to the Author):

Thank you for providing the revised manuscript and the rebuttal letter. The manuscript has been substantially improved, and the major concerns raised during the initial review have been addressed effectively.

In particular, the added multivariable analyses, clearer treatment of inter-cohort variability, explicit discussion of longitudinal limitations, and more restrained interpretation of causality significantly strengthen the work. The definition and presentation of the axon-guidance–associated SGA endotype are now consistent and well justified, and the positioning of the sheep model as exploratory is appropriate.

I do not have additional major comments.

No further comments.
